# Patterns and temporal trends of comorbidity among adult patients with incident cardiovascular disease in the UK between 2000 and 2014: A population-based cohort study

**DOI:** 10.1371/journal.pmed.1002513

**Published:** 2018-03-06

**Authors:** Jenny Tran, Robyn Norton, Nathalie Conrad, Fatemeh Rahimian, Dexter Canoy, Milad Nazarzadeh, Kazem Rahimi

**Affiliations:** 1 George Institute for Global Health, University of Oxford, Oxford, United Kingdom; 2 Deep Medicine, Oxford Martin School, Oxford, United Kingdom; 3 George Institute for Global Health, University of New South Wales, Sydney, New South Wales, Australia; 4 Collaboration Center of Meta-Analysis Research, Torbat Heydariyeh University of Medical Sciences, Torbat Heydariyeh, Iran; 5 Oxford University Hospitals NHS Foundation Trust, Oxford, United Kingdom; National University of Singapore, SINGAPORE

## Abstract

**Background:**

Multimorbidity in people with cardiovascular disease (CVD) is common, but large-scale contemporary reports of patterns and trends in patients with incident CVD are limited. We investigated the burden of comorbidities in patients with incident CVD, how it changed between 2000 and 2014, and how it varied by age, sex, and socioeconomic status (SES).

**Methods and findings:**

We used the UK Clinical Practice Research Datalink with linkage to Hospital Episode Statistics, a population-based dataset from 674 UK general practices covering approximately 7% of the current UK population. We estimated crude and age/sex-standardised (to the 2013 European Standard Population) prevalence and 95% confidence intervals for 56 major comorbidities in individuals with incident non-fatal CVD. We further assessed temporal trends and patterns by age, sex, and SES groups, between 2000 and 2014. Among a total of 4,198,039 people aged 16 to 113 years, 229,205 incident cases of non-fatal CVD, defined as first diagnosis of ischaemic heart disease, stroke, or transient ischaemic attack, were identified. Although the age/sex-standardised incidence of CVD decreased by 34% between 2000 to 2014, the proportion of CVD patients with higher numbers of comorbidities increased. The prevalence of having 5 or more comorbidities increased 4-fold, rising from 6.3% (95% CI 5.6%–17.0%) in 2000 to 24.3% (22.1%–34.8%) in 2014 in age/sex-standardised models. The most common comorbidities in age/sex-standardised models were hypertension (28.9% [95% CI 27.7%–31.4%]), depression (23.0% [21.3%–26.0%]), arthritis (20.9% [19.5%–23.5%]), asthma (17.7% [15.8%–20.8%]), and anxiety (15.0% [13.7%–17.6%]). Cardiometabolic conditions and arthritis were highly prevalent among patients aged over 40 years, and mental illnesses were highly prevalent in patients aged 30–59 years. The age-standardised prevalence of having 5 or more comorbidities was 19.1% (95% CI 17.2%–22.7%) in women and 12.5% (12.0%–13.9%) in men, and women had twice the age-standardised prevalence of depression (31.1% [28.3%–35.5%] versus 15.0% [14.3%–16.5%]) and anxiety (19.6% [17.6%–23.3%] versus 10.4% [9.8%–11.8%]). The prevalence of depression was 46% higher in the most deprived fifth of SES compared with the least deprived fifth (age/sex-standardised prevalence of 38.4% [31.2%–62.0%] versus 26.3% [23.1%–34.5%], respectively). This is a descriptive study of routine electronic health records in the UK, which might underestimate the true prevalence of diseases.

**Conclusions:**

The burden of multimorbidity and comorbidity in patients with incident non-fatal CVD increased between 2000 and 2014. On average, older patients, women, and socioeconomically deprived groups had higher numbers of comorbidities, but the type of comorbidities varied by age and sex. Cardiometabolic conditions contributed substantially to the burden, but 4 out of the 10 top comorbidities were non-cardiometabolic. The current single-disease paradigm in CVD management needs to broaden and incorporate the large and increasing burden of comorbidities.

## Abstract 2

Using data from a primary care database in the UK, Kazem Rahimi and colleagues examine the patterns and temporal trends of comorbidity among patients with incident cardiovascular disease between 2000 and 2014

## Author summary

**Why was this study done?:**

- Multimorbidity is common and has been shown to relate to a range of determinants including age, sex, and socioeconomic status in the general population.
- However, in patients with cardiovascular disease (CVD), reliable, large-scale, contemporary evidence on patterns and trends of multimorbidity is limited.
- Indeed, prior research has mainly investigated comorbidities in patients with prevalent CVD, which might be subject to time-dependent biases.

**What did the researchers do and find?:**

- We describe the prevalence of 56 clinically important and common comorbidities in 229,205 patients with newly diagnosed non-fatal CVD from 4.2 million patient electronic health records in the United Kingdom between 2000 and 2014.
- We show that even in age-standardised models, the proportion of patients with 5 or more conditions has increased by 4 times between 2000 and 2014.
- Although cardiometabolic conditions contributed substantially to this burden, 4 out of the 10 top comorbidities were non-cardiometabolic.
- On average, older patients, women, and socially deprived groups had higher numbers of comorbidities, but the type of comorbidities varied by age and sex.

**What do these findings mean?:**

- This comprehensive analysis of patterns and temporal trends in comorbidities shows that in parallel to the decreasing CVD incidence, the profile of patients is changing rapidly.
- The findings suggest that the clinical care of patients with incident CVD is becoming more complex and very different from what has been typically assessed in trial populations.
- Overall, they challenge the current single-disease paradigm in CVD management and call for incorporation of the large and increasing burden of comorbidities in research and care planning.

## Introduction

Comorbidities, defined as the presence of additional diseases with reference to an index disease [1], are associated with functional impairment and poor prognosis for the individuals affected. They also have important implications for health policy and healthcare provision as they can lead to higher hospital admission rates, longer in-hospital stays, more complications, and, consequently, higher resource utilisation [2,3].

Over the past few decades, an unprecedented decline in cardiovascular disease (CVD) incidence and mortality has been observed [4]. However, with increasing life expectancy and higher survival rates, there is an expectation that individuals with incident CVD will have multimorbidity, commonly defined as the presence of 2 or more conditions in the same individual [5], that will affect their well-being, healthcare preferences, and treatment options.

Despite the importance of this issue, to our knowledge, no previous study has investigated the patterns and temporal trends in comorbidity among incident CVD patients, and reports of the presence of comorbidities among patients with prevalent CVD have been typically confined to investigation of a few chronic conditions [6,7], limited to specific subgroups [8,9], or based on information from hospital admissions, which may miss reports on important outpatient diagnoses [10,11].

We, therefore, used a large longitudinal database of linked electronic health records to investigate the burden of comorbidity in patients with incident non-fatal CVD, defined as a diagnosis of ischaemic heart disease (IHD), stroke, or transient ischaemic attack (TIA). We extracted information for 56 major comorbid chronic conditions in these patients and investigated how their prevalence has changed over the past 15 years and how they varied by age, sex, and socioeconomic status (SES). We further compared crude and age/sex-standardised prevalence in order to gain a better understanding of how any observed differences in comorbidity patterns might be due to population ageing versus other factors.

## Methods

### Data source

The study was conducted using linked electronic health records from the UK Clinical Practice Research Datalink (CPRD) study from its inception in 1 January 1985 to 30 September 2015 (https://www.cprd.com/home/). The CPRD database contains pseudo-anonymised patient data from 674 general practices in the UK, covering approximately 7% of the current UK population, and it is broadly representative of the UK population by age, sex, and ethnicity [12]. It links primary care records with discharge diagnoses from Hospital Episode Statistics (HES) and mortality data from national death registries (Office for National Statistics), with a coding system equivalent to the World Health Organization 10th revision of the International Statistical Classification of Diseases (ICD-10) [13]. The dataset is one of the most comprehensive prospective primary care databases, the validity of which has previously been reviewed elsewhere [14,15]. Scientific approval for this study was given by the CPRD Independent Scientific Advisory Committee (protocol number 16_049R), and no additional informed consent was required as there was no individual patient involvement [16].

### Study population

We identified patients who had a first diagnosis of incident non-fatal CVD defined as any report of IHD (including acute myocardial infarction and angina), stroke, or TIA recorded between 1 January 2000 and 31 December 2014 in CPRD or HES, so long as the patient had at least 12 months of registration at the practice, the diagnosis was made after the first 12 months of their current registration period [17], the practice was deemed to be contributing ‘up-to-standard’ data, the patient was 16 years old or older, the patient’s individual record was marked by CPRD to be ‘acceptable’ quality data for research purposes, and the patient’s CPRD record had linkage to HES. Prevalent CVD cases were excluded by excluding patients for whom a CVD diagnosis was recorded before the patient was eligible for inclusion in the study. We restricted incident CVD events to those that were non-fatal because we were interested in the comorbidity burden in patients who continue to live after an incident CVD event.

### Selection and definition of comorbidities

We selected 56 comorbidities that are considered clinically significant and are most prevalent in the UK (Table 1). The comorbidities were selected based on 3 sources: (1) the Quality and Outcomes Framework (QOF), an incentive scheme for general practitioners in the UK [18], (2) the Charlson comorbidity index, the most commonly used comorbidity index, originally designed to predict inpatient hospital mortality [19], and (3) the multiple chronic conditions list of the US Department of Health and Human Services Initiative on Multiple Chronic Conditions [20]. For each comorbidity, a list of diagnostic codes from hospital (ICD-10) and primary care (Read) coding schemes was used to identify diagnoses. The codes were compiled from online code repositories, including Cardiovascular Disease Research Using Linked Bespoke Studies and Electronic Health Records (CALIBER) [21], and medical dictionary keyword searches (S1 Table). Diagnosis of a prevalent comorbidity was defined as the recording of a diagnostic code for that comorbidity before the date of CVD incidence. We did not include diagnoses after the date of diagnosis of CVD.

**Table 1:** Comorbidities selected.

Group	Comorbidity	Included conditions
Cardiometabolic	Cardiac arrhythmia	Cardiac arrhythmias and dysrhythmias, including atrial fibrillation and flutter, paroxysmal tachycardia, atrioventricular blocks, and other conduction disorders
	Chronic kidney disease	Chronic kidney disease stage 3 or more, or dependence on transplant or dialysis
	Diabetes mellitus	Includes type I and II, and diabetes-specific sequelae
	Heart failure	
	Hyperlipidaemia	
	Hypertension	Includes hypertensive-specific sequelae
	Obesity	
	Peripheral arterial disease	Includes aortic aneurysm and dissection, embolism and thrombosis, and unspecified peripheral vascular diseases
Mental health	Adjustment disorder	Includes pathological reactions to severe stress
	Affective disorder	Unspecified, persistent, or other affective disorders
	Anxiety	Anxiety and phobic disorders
	Bipolar disorder	
	Depression	Depression and depressive mood disorders
	Eating disorder	Eating disorders, including bulimia and anorexia nervosa
	Psychoses	Psychotic and delusional disorders
	Schizophrenia	
	Substance abuse	Mental and behavioural disorders due to substances including alcohol, opioids, and cannabinoids
Respiratory	Asthma	
	Chronic obstructive pulmonary disease	
	Lung cancer	
	Other respiratory cancer	Respiratory organ cancer excluding lung cancer, e.g., laryngeal cancer, pleural cancer, and tracheal cancer
Musculoskeletal	Arthritis	Chronic arthritis, including osteoarthritis, secondary arthritides (e.g., arthropathy in Crohn disease and psoriatic arthritis), and other unspecified arthritides and sequelae; excluding acute arthritis (e.g., septic arthritis) and arthritides covered elsewhere: gout, rheumatoid arthritis, and connective-tissue-related arthritis
	Gout	
	Osteoporosis	
	Rheumatoid arthritis	
Neurological	Dementia	Includes Alzheimer disease, vascular dementia, and unspecified dementia
	Epilepsy	
	Hemiplegia	
	Learning disability	Disorders of psychological development, mental retardation, and childhood autism
Cancers	Bladder cancer	
	Breast cancer	
	Cervical cancer	
	Colon cancer	
	Ear, nose, and throat cancer	
	Leukaemia	
	Liver cancer	
	Lymphoma	
	Metastatic cancer	
	Oesophageal cancer	
	Ovarian cancer	
	Pancreatic cancer	
	Prostate cancer	
	Rectal cancer	
	Renal cancer	
	Skin cancer	
	Stomach cancer	
	Other female reproductive cancer	Female reproductive organ cancer excluding breast, ovarian, and cervical cancer, e.g., endometrial cancer, uterine cancer, and cancer of the vagina
	Other gastrointestinal cancer	Gastrointestinal cancer excluding oesophageal, stomach/gastric, colon, rectal, pancreatic, and liver cancer, e.g., cancer of the bile ducts, anal cancer, and peritoneal cancer
	Other male reproductive cancer	Male reproductive organ cancer excluding prostate cancer, e.g., testicular cancer
	Other urological cancer	Urological organ cancer excluding renal/kidney and bladder cancer, e.g., cancer of the ureter and cancer of the urethra
	Unspecified cancer	Cancer with unspecified anatomical region or origin
	Other cancer	Cancer not otherwise covered by all other cancers covered in the list of 56 comorbidities, e.g., brain cancer, cancer of the eye, and cancer of the endocrine glands
Other	Connective tissue disease	Systemic connective tissue disorders including systemic lupus erythematosus, systemic sclerosis, and polymyositis
	HIV/AIDS	
	Liver disease	Includes cirrhosis, portal hypertension, and hepatic failure; excludes liver cancer
	Peptic ulcer disease	Includes gastric and duodenal ulcers

Cancers exclude benign cancers. A comprehensive definition of each condition based on ICD-10 diagnostic codes is provided in S1 Table.

### Statistical analyses

Baseline characteristics (systolic blood pressure [SBP] and diastolic blood pressure [DBP], smoking status, and body mass index [BMI]) for patients with incident CVD were taken as the most recent measurements taken before diagnosis of CVD. BMI was stratified into ‘underweight’ (<18.5 kg/m^2^), ‘normal’ (18.5–24.9 kg/m^2^), ‘overweight’ (25–29.9 kg/m^2^), and ‘obese’ (≥30 kg/m^2^). We also extracted data on age, sex, SES (refer to below), and ethnicity. Results are presented as frequencies and percentages for categorical data, and means and standard deviations for continuous data, stratified by sex and SES (using fifths of the Index of Multiple Deprivation [IMD]) for all CVD patients. The key variables age, sex, and SES had complete data.

SES was categorised into fifths using the IMD 2015 split by even quintiles; this categorisation is widely used in the UK in healthcare research [22,23]. The IMD is a measure of SES that uses small area boundaries covering approximately 1,500 people to calculate a deprivation score that covers 7 broad dimensions of deprivation: income, employment, education, health, crime, housing, and living environment [24].

Annual incidence rates for IHD, cerebrovascular disease (stroke/TIA), and overall CVD were calculated by dividing the number of incident cases by the number of patient-years at risk in the cohort for the year of interest. Time at risk for each patient was restricted to start from the latest of the following: the start of the year, date of current registration with the practice, or the practice’s up-to-standard date, and was specified to finish at the earliest of the following: the end of the year, date of death, date of transfer out of practice, or the practice’s last date of data collection.

Prevalence for each comorbidity was calculated by dividing the number of incident CVD cases with that comorbidity by the number of all patients diagnosed with CVD meeting the study inclusion criteria. Annual prevalence included all cases with a diagnosis recorded by 1 July of the year of interest. The number of comorbidities per patient was reported in categories of 0, 1, 2, 3, 4, and >5. Additionally, we classified patients as multimorbid versus not by defining multimorbidity as the presence of 1 or more comorbidities (in addition to incident CVD) [5]. Where appropriate, age- and/or sex-standardised incidence rates and proportions were computed by applying direct age standardisation to the 2013 European Standard Population [25] using 10-year age bands up to 90+ years old, and averaging the sex-specific age-standardised values. Prevalences for sex-specific conditions—breast cancer, cervical cancer, ovarian cancer, other female reproductive cancers, prostate cancer, and other male reproductive cancers—were not sex-standardised.

Prevalences were calculated for patient groups stratified by age group (10-year age groups: <20, 20–29, 30–39, 40–49, 50–59, 60–69, 70–79, 80–89, ≥90 years), sex, SES group (fifths of deprivation index), and year. Annual prevalences were plotted and fitted using locally weighted non-parametric smoothing if necessary.

The estimated prevalence proportions are presented as percentages with 95% confidence intervals. Confidence intervals for crude prevalence were calculated using the Wilson score method for binomial proportions, which is required to make reliable comparisons between different groups. Stratification may give small numbers of events in some patient groups; therefore, the normal approximation method of calculating CIs may give unusual CIs in lower or upper bounds (such as negative values) [26]. Confidence intervals for multinomial proportions for crude prevalence were calculated using the Sison and Glaz method [27]. The chi-squared test was used to test for association between 2 proportions for categorical variables, and the 2-sample *t* test for difference between means of categorical variables.

All analyses were prospectively specified in the study protocol (S1 Text), and all statistical analyses were performed using R version 3.3.2 [28]. Reporting of this study was done in accordance with the REporting of studies Conducted using Observational Routinely collected health Data (RECORD) guidelines (S2 Text) [29].

## Results

### Baseline characteristics

From a cohort of 4,198,039 patients with 29,110,997 person-years at risk, 229,205 patients with incident non-fatal CVD were identified. The mean age at the diagnosis was 71 years (range 16 to 113 years), with the majority of patients aged between 50 and 89 years (see S1 Fig for age distribution at the time of diagnosis).

Baseline characteristics of patients overall and by sex and SES are presented in Table 2. Nearly half of the patients (48%) with incident non-fatal CVD were women, who were, on average, 6 years older than men with incident non-fatal CVD (74 versus 68 years, *p <* 0.001). Women were less likely to be current smokers (19.2% versus 25.0%, *p <* 0.001) and less likely to be overweight (31.8% versus 40.9%, *p <* 0.001) compared to men. Compared to the least deprived fifth, patients in the most deprived fifth were more likely to be obese (34.3% versus 25.5%, *p <* 0.001) and current smokers (34.0% versus 14.8%, *p <* 0.001). Mean (SD) SBP and DBP was 140 (SD 22) mm Hg and 80 (SD 12) mm Hg, respectively, and blood pressure varied little between sexes and across SES categories. Patients with incident cerebrovascular disease, compared to incident IHD, were older (73.7 versus 68.7 years old), were more likely to be female (55.5% versus 43.8%), and had higher blood pressure (mean SBP 142.2 mm Hg versus 139.8 mm Hg).

**Table 2:** Baseline characteristics of patients with CVD at time of diagnosis.

Characteristics	All CVD	CVD subtype		Sex		SES	
		Ischaemic heart disease	Cerebrovascular disease	Women	Men	Least deprived fifth	Most deprived fifth
**Number of patients**	229,205	140,828	88,377	110,803	118,402	48,801	37,964
**Age (years), mean (SD)**	70.6 (13.8)	68.7 (13.4)	73.7 (14.0)	73.8 (13.6)	67.6 (13.3)	71.5 (13.3)	68.4 (14.5)
**Women, percent (*n*)**	48.3 (110,803)	43.8 (61,733)	55.5 (43,428)	100 (110,803)	—	46.8 (22,854)	49.8 (18,906)
**White ethnicity, percent (*n*)**	97.0 (113,840) [111,833]	96.7 (70,412) [67,990]	97.5 (43,428) [43,843}	97.3 (54,507) [54,803]	96.7 (59,333) [57,30]	97.8 (24,071) [24,194]	95.5 (19,834) [17,198]
**Fifths of deprivation index, percent (*n*)**							
Q1 (least deprived)	21.3 (48,801)	21.0 (29,504)	21.8 (19,297)	20.6 (22,852)	21.9 (25,949)	100 (48,801)	—
Q2	22.2 (50,786)	22.0 (31,044)	22.3 (19,742)	21.9 (24,278)	22.4 (26,508)	—	—
Q3	21.2 (48,647)	21.1 (29,648)	21.5 (18,999)	21.3 (23,570)	21.2 (25,077)	—	—
Q4	18.6 (42,672)	18.8 (26,491)	18.3 (16,181)	19.0 (21,034)	18.3 (21,638)	—	—
Q5 (most deprived)	16.6 (37,964)	17.0 (23,949)	15.9 (14,015)	17.1 (18,906)	16.1 (19,058)	—	100 (37,964)
**SBP (mm Hg), mean (SD)**	140.7 (22) [7,189]	139.8 (21.4) [4,097]	142.2 (22.8) [3,092]	141.6 (22.9) [2,912]	139.9 (21.2) [4,270]	141.2 (20.9) [1,257]	139.8 (22.8) [1,498]
**DBP (mm Hg), mean (SD)**	79.6 (12.2) [7,141]	79.4 (12.1) [4,068]	79.9 (12.4) [3,073]	79.0 (12.1) [2,890]	80.2 (12.3) [4,251]	79.8 (12.1) [1,252]	79.4 (12.8) [1,482]
**Body mass index (kg/m**^**2**^**), percent (*n*)**							
Underweight	3.4 (3,756)	2.6 (1,847)	4.9 (1,909)	5.1 (2,702)	1.8 (1,054)	3.2 (717)	3.8 (747)
Normal	30.4 (33, 834)	27.1 (19,542)	36.5 (14,292)	33.1 (17,670)	27.9 (16,164)	32.8 (7,382)	28.6 (5,643)
Overweight	36.6 (40,667)	37.6 (27,058)	34.7 (13,609)	31.8 (16,976)	40.9 (23,691)	38.6 (8,687)	33.3 (6,575)
Obese	29.6 (32,957) [117,991]	32.7 (23,574) [68,807]	23.9 (9,383) [49,184]	30.0 (15,989) [57,466]	29.3 (16,968) [60,525]	25.5 (5,741) [26,274]	34.3 (6,781) [18,218]
**Current smokers, percent (*n*)**	22.3 (33,246) [80,051]	22.7 (21,394) [46,458]	21.6 (11,852) [33,593]	19.2 (13,459) [40,743]	25.0 (19,787) [39,308]	14.8 (4,583) [17,808]	34.0 (8,745) [12,251]

The category percentages refers to complete cases. Numbers in square brackets refer to the number of patients with missing data for the relevant characteristic. SES refers to the Index of Multiple Deprivation 2015 cut by quintiles, where Q1 is the least deprived fifth and Q5 is the most deprived fifth of the population. BMI categories are ‘underweight’ (<18.5 kg/m^2^), ‘normal’ (18.5–24.9 kg/m^2^), ‘overweight’ (25–29.9 kg/m^2^), and ‘obese’ (≥30 kg/m^2^).

CVD, cardiovascular disease; DBP, diastolic blood pressure; SES, socioeconomic status; SBP, systolic blood pressure.

### Temporal trends in incidence of CVD

**Fig 1:**
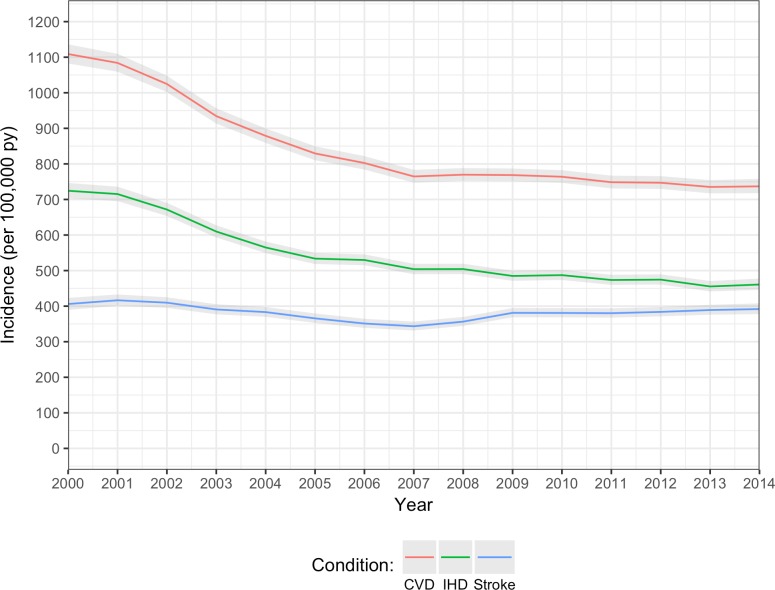
Annual age/sex-standardised incidence of CVD, IHD, and stroke/TIA. Overall CVD (red line); IHD (including acute myocardial infarction and angina; green line); stroke/TIA (blue line). CVD, cardiovascular disease; IHD, ischaemic heart disease; TIA, transient ischaemic attack.

In 2000, age/sex-standardised incidence of non-fatal CVD was 1,109 (95% CI 1,082–1,136) per 100,000 person-years, but fell to 737 (718–754) per 100,000 person-years in 2014, a relative reduction of 34% over this 15-year period (Fig 1). A similar pattern was seen for incidence of IHD and stroke/TIA, although the reduction in incidence rate for stroke/TIA appeared to be less steep than that for IHD. Mean age at diagnosis of non-fatal CVD remained stable between 70 and 71 years between 2000 and 2014 (70.7 years in 2000, 70.5 years in 2014), and the 6-year age difference between men and women was maintained (S2 Table). Men had a higher incidence of overall CVD (and IHD and stroke/TIA) than women, and the sex difference remained relatively constant over time (S2 Fig).

### Prevalence of multimorbidity and number of comorbidities

In patients with incident CVD, the crude and age/sex-standardised prevalence of multimorbidity (defined as 1 or more comorbidities in addition to the index CVD condition) were 91.0% (95% CI 90.9%–91.1%) and 81.1% (78.7%–83.8%), respectively. For having 5 or more conditions, the crude and age/sex-standardised prevalences were 25.3% (25.1%–25.5%) and 15.0% (14.3%–16.1%), respectively (S3 Table).

**Fig 2:**
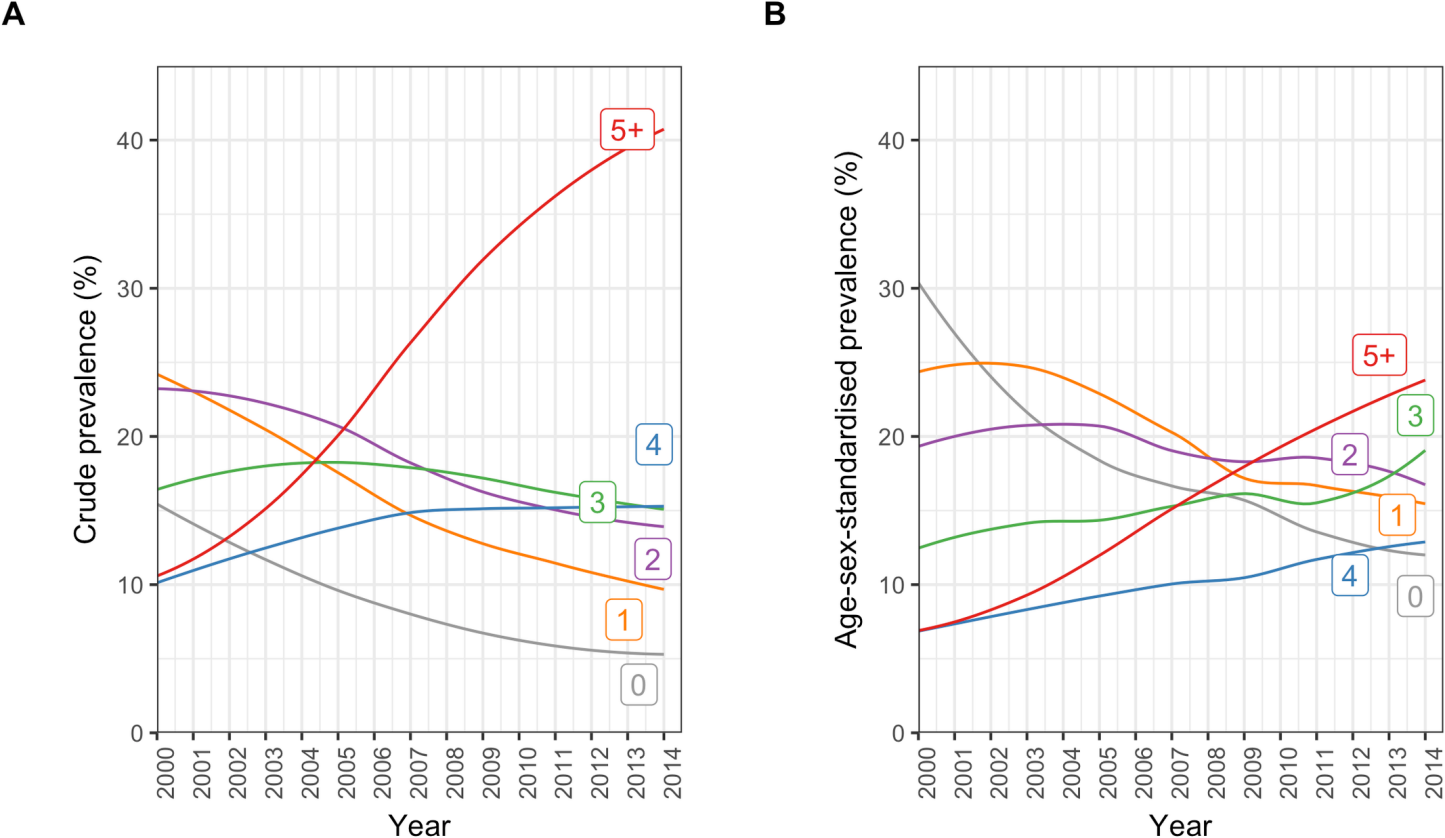
Annual crude and age/sex-standardised prevalence of number of comorbidities in incident cardiovascular disease patients. (A) Crude prevalence. (B) Age/sex-standardised prevalence. Number labels for each line refer to the number of comorbidities.

There was an increasing number of comorbidities in incident CVD patients from 2000 to 2014 (Fig 2; S3 Table). Crude prevalence of having 5 or more comorbidities increased by 2.9-fold from 10.6% (95% CI 9.7%–11.4%) in 2000 to 40.9% (39.9%–41.8%) in 2014. Similarly, in age/sex-standardised analysis, there was a 2.8-fold increase, from 6.3% (95% CI 5.6%–17.0%) in 2000 to 24.3% (22.1%–34.8%) in 2014. Within the group of patients with 5 or more comorbidities, crude and age/sex-standardised prevalence of number of comorbidities increased up to the maximum observed value of 17 comorbidities in any 1 patient.

**Fig 3:**
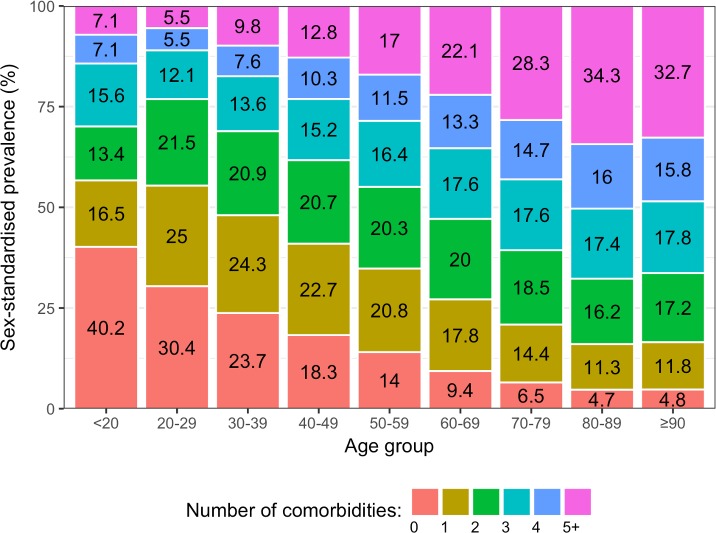
Sex-standardised prevalence of number of conditions in patients with incident cardiovascular disease by age group (years).

Among incident CVD patients, older age groups tended to have higher proportions of multiple comorbidities than younger age groups (Fig 3). In people who were ≥90 years, the sex-standardised prevalence of having 5 or more comorbidities was 6.9 times the prevalence of having no comorbidities (32.7% [95% CI 31.7%–34.0%] and 4.8% [3.6%–5.9%], respectively), whilst this pattern was reversed in people who were <20 years, where the sex-standardised prevalence of having no comorbidities was 5.6 times the prevalence of having 5 or more comorbidities (40.2% [13.0%–75.3%] and 7.1% [0.0%–40.5%], respectively). Patterns in crude prevalence were similar to those of sex-standardised prevalence (S3 Fig).

The age-standardised prevalence of number of comorbidities across the whole study period varied by sex (S4 Fig). The age-standardised prevalence of having 4 or more comorbidities was higher in women, suggesting that the older age of women was not the only reason for the higher burden of comorbidity among them. The age-standardised prevalence of having 5 or more comorbidities was 19.1% (95% CI 17.2%–22.7%) in women and 12.5% (12.0%–13.9%) in men.

**Fig 4:**
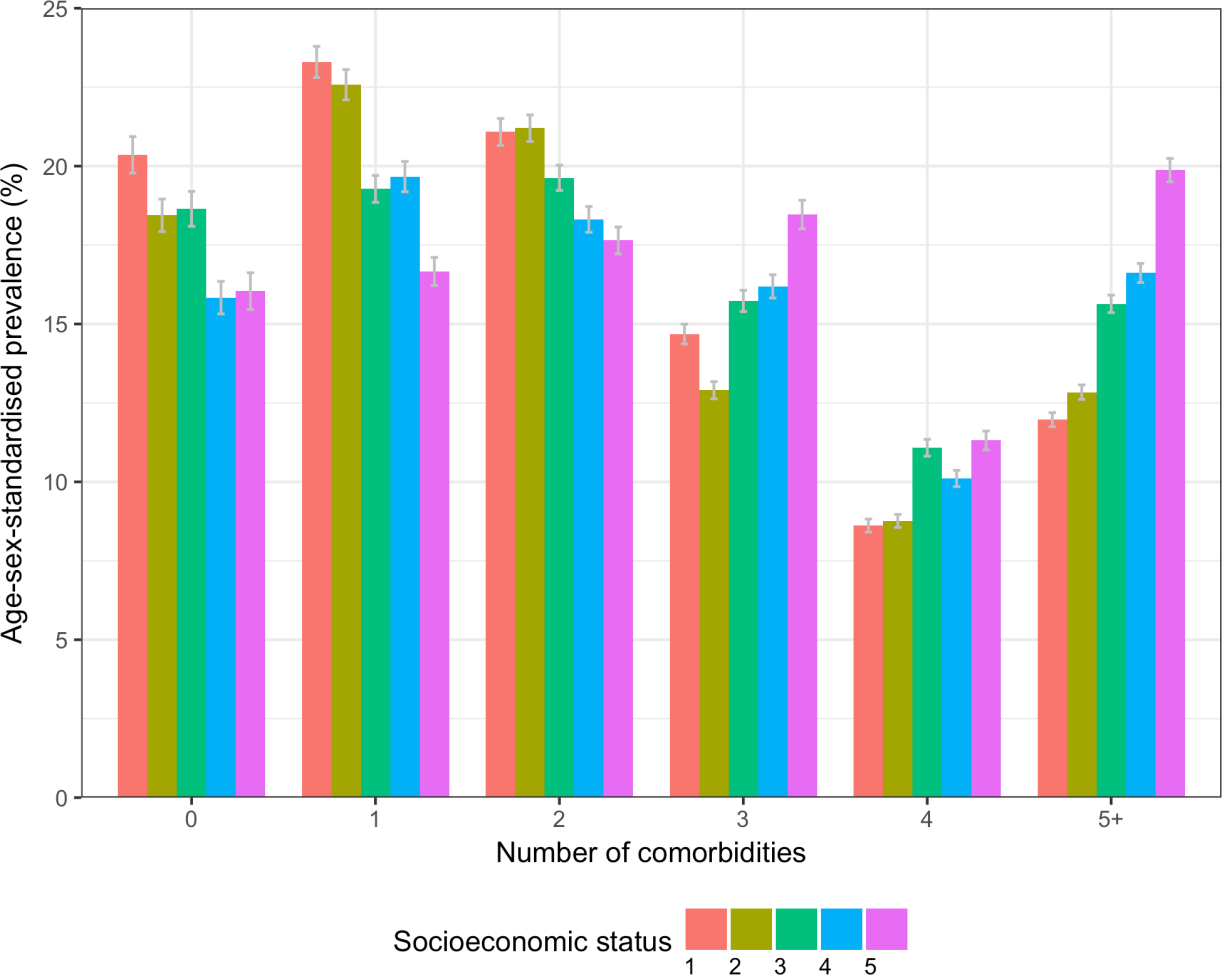
Age/sex-standardised prevalence of number of comorbidities in patients with incident cardiovascular disease by socioeconomic status. Socioeconomic status is split by quintiles of the Index of Multiple Deprivation for 2015, where 1 = the least deprived fifth, and 5 = the most deprived fifth.

Fig 4 shows age/sex-standardised prevalence of number of comorbidities comparing patterns among patients by SES group. There was a general pattern of increasing prevalence of higher numbers of comorbidities with increasing deprivation. Those who were socioeconomically more deprived had a higher prevalence of higher numbers of comorbidities, with the age/sex-standardised prevalence of having 5 or more conditions increasing from 12.0% (95% CI 10.7%–19.1%) in the least deprived fifth to 19.9% (18.7%–40.8%) in the most deprived fifth.

### Prevalence of specific comorbidities

The most prevalent comorbidities among all incident CVD patients between 2000 and 2014 were in the condition categories cardiometabolic, mental illness, and musculoskeletal (Table 3). The most prevalent conditions in age/sex-standardised models were hypertension (28.9% [95% CI 27.7%–31.4%]), depression (23.0% [21.3%–26.0%]), arthritis (20.9% [19.5%–23.5%]), asthma (17.7% [15.8%–20.8%]), and anxiety (15.0% [13.7%–17.6%]) (S4 Table).

**Table 3:** Age/sex-standardised prevalence of comorbidities in patients with incident cardiovascular disease (*n =* 229,205).

Comorbidity	*N*	Age/sex-standardised prevalence, percent (95% CI)
Hypertension	109,365	28.9 (27.7–31.5)
Depression	42,973	23.0 (21.3–26.0)
Arthritis	85,527	20.9 (19.5–23.5)
Asthma	31,103	17.7 (15.8–20.8)
Anxiety	30,255	15.0 (13.7–17.6)
Hyperlipidaemia	35,304	11.3 (10.7–13.6)
Diabetes mellitus	33,307	11.2 (10.1–13.7)
Obesity	22,315	10.8 (10.2–12.9)
Cardiac arrhythmia	27,695	7.1 (6.0–9.6)
Chronic kidney disease	24,314	5.3 (4.9–7.4)
Substance abuse	6,768	5.1 (4.6–7.4)
Chronic obstructive pulmonary disease	20,363	4.5 (4.3–6.6)
Adjustment disorder	5,077	4.4 (2.8–7.5)
Heart failure	19,090	4.1 (3.8–6.3)
Peripheral arterial disease	16,522	3.9 (3.6–6.0)
Epilepsy	5,057	3.6 (3.0–6.0)
Gout	14,255	3.4 (3.2–5.6)
Peptic ulcer disease	13,249	3.4 (3.2–5.5)
Other female reproductive cancer	2,346	3.2 (1.6–7.1)
Osteoporosis	15,655	2.9 (2.5–5.2)

The top 20 comorbidities ranked by age/sex-standardised prevalence are shown.

**Fig 5:**
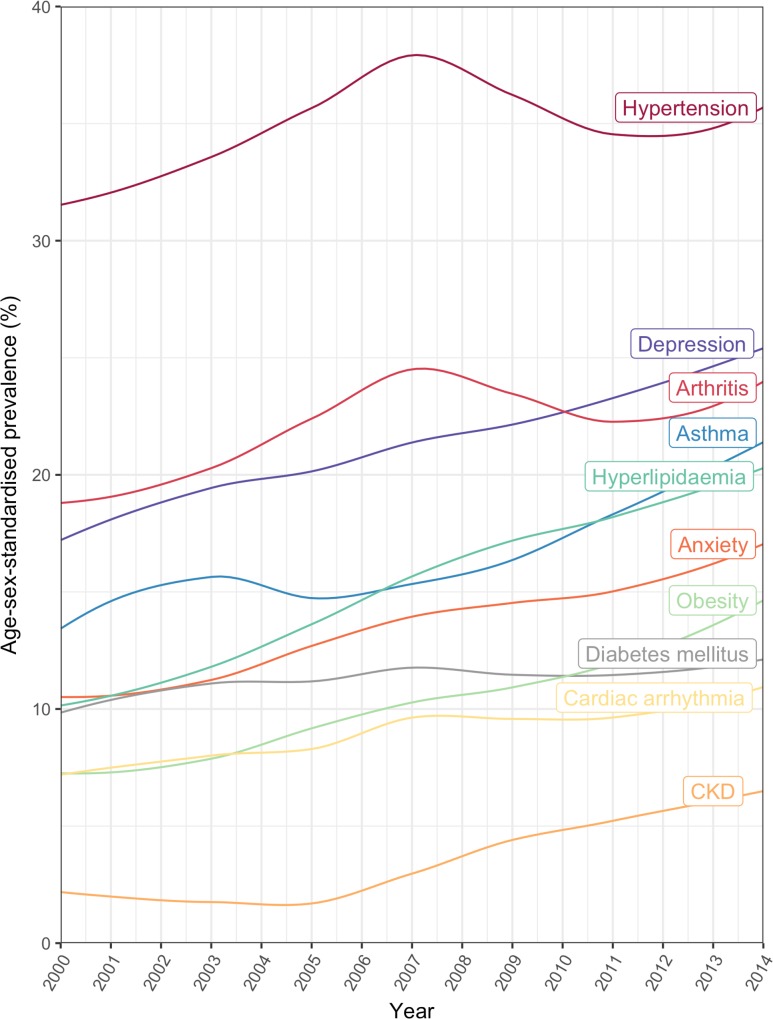
Annual prevalence of the 10 most common comorbidities in patients with incident cardiovascular disease between 2000 and 2014. CKD, chronic kidney disease.

Annual age/sex-standardised prevalence increased over time for most comorbidities (Fig 5; S4 Table). Many of the highly prevalent comorbidities increased from 2000 to 2014 in age/sex-standardised models, including hypertension, which increased by 11.9% from 32.0% (95% CI 26.9%–45.9%) in 2000 to 35.8% (34.4%–40.1%) in 2014, and depression, which increased by 47.1% from 16.6% (13.8%–30.2%) in 2000 to 24.5% (22.7%–29.0%) in 2014. Of the top 10 comorbidities ranked by age/sex-standardised prevalence, the largest increase between 2000 and 2014 was seen in chronic kidney disease (321.0% increase, 1.6% [95% CI 0.5%–15.5%] to 6.6% [5.8%–10.6%]), followed by hyperlipidaemia (97.3% increase, 10.5% [8.4%–23.6%] to 20.6% [19.4%–24.6%]) and obesity (94.6% increase, 7.6% [5.8%–21.1%] to 14.9% [13.4%–19.2%]). For some conditions, such a hypertension and arthritis, a peak in prevalence was seen in 2007. To investigate whether the increase in prevalence of conditions was due to the introduction of QOF, we stratified results by QOF and non-QOF conditions (S5 Fig); the majority of conditions in both groups had generally increasing prevalence over time.

**Fig 6:**
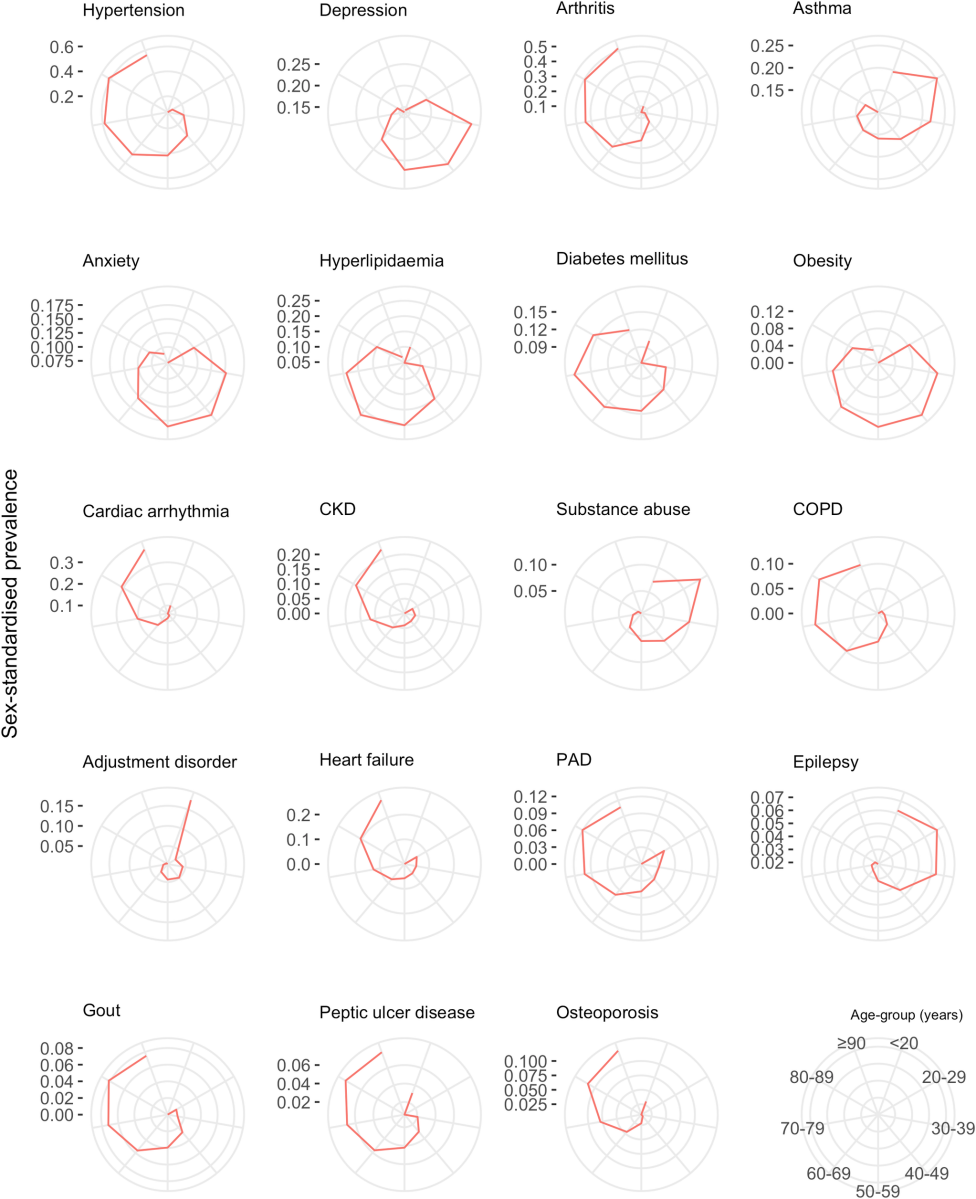
Sex-standardised prevalence of comorbidities in patients with incident cardiovascular disease by age group (years). The spokes of each radial plot represent increasingly older age groups going clockwise from the first spoke after 12 o’clock (legend represented in bottom right of figure). The diameter axes show sex-standardised prevalence, and axis ticks/scale are specifically labelled for each plot. The most prevalent non-sex-specific conditions are shown. CKD, chronic kidney disease; COPD, chronic obstructive pulmonary disease; PAD, peripheral arterial disease.

The age-specific prevalence of comorbidities stratified by 10-year age groups is shown in Fig 6. There was an age-specific pattern of comorbidities, with a high prevalence of cardiometabolic conditions and arthritis in older age groups, and a high prevalence of mental illness in younger age groups (Table 4). Hypertension had the highest prevalence in 80–89 year olds (62.7% [95% CI 62.3%–63.1%]), depression had highest prevalence in 30–39 year olds (27.6% [26.1%–29.2%]), and anxiety had the highest prevalence in 40–49 year olds (17.3% [16.7%–17.9%]). Table 4 shows the top 10 most prevalent comorbidities by sex-standardised prevalence in each age group. Cardiometabolic conditions were highly prevalent in all age groups, but even more so among older age groups. Hypertension was the most prevalent comorbidity in patients ≥40 years and increased with age; the prevalence in 40–49 year olds (31.5% [95% CI 30.8%–32.3%]) was half that of 80–89 year olds (62.7% [62.3%–63.1%]). Arthritis also increased in prevalence with age and was ranked higher in older age groups, rising from ninth in 30–39 year olds (8.0% [95% CI 7.1%–9.0%]) to the second most prevalent comorbidity by age 60–69 years (34.8% [34.4%–35.2%]), and maintaining second place until age ≥90 years (53.5% [52.7%–54.2%]). Mental illnesses were highly ranked in younger age groups, with depression being the most prevalent comorbidity in 30–39 year olds (27.6% [95% CI 26.1%–29.2%]) and reducing in prevalence to rank eighth by age ≥90 years (15.1% [14.6%–15.7%]).

**Table 4:** Age-specific ranking of the top 10 most prevalent comorbidities.

Ranking	Age group (years)																	
	<20		20–29		30–39		40–49		50–59		60–69		70–79		80–89		≥90	
1	Asthma	21.7	Asthma	25.1	Depression	27.6	Hypertension	31.5	Hypertension	41.4	Hypertension	50.9	Hypertension	58.6	Hypertension	62.7	Hypertension	58.3
2	Learning disability	17.4	Depression	19.1	Asthma	20.8	Depression	26.5	Hyperlipidaemia	25.4	Arthritis	34.8	Arthritis	43.8	Arthritis	50.7	Arthritis	53.5
3	Adjustment disorder	13.0	Substance abuse	14.5	Hypertension	20.3	Hyperlipidaemia	21.0	Depression	24.3	Hyperlipidaemia	26.8	Hyperlipidaemia	24.0	Cardiac arrhythmia	30.5	Cardiac arrhythmia	36.9
4	Cardiac arrhythmia	8.7	Anxiety	12.4	Anxiety	17.0	Anxiety	17.3	Arthritis	23.1	Depression	20.5	Cardiac arrhythmia	20.4	Heart failure	20.4	Heart failure	27.4
5	Diabetes mellitus	8.7	Hypertension	11.4	Obesity	13.3	Asthma	16.3	Anxiety	16.7	Diabetes mellitus	16.3	Diabetes mellitus	17.9	Chronic kidney disease	18.9	Chronic kidney disease	22.2
6	Arthritis	8.7	Obesity	8.3	Hyperlipidaemia	11.5	Obesity	14.6	Diabetes mellitus	14.5	Asthma	14.5	Depression	16.8	Depression	16.7	Dementia	18.6
7	Hemiplegia	8.7	Epilepsy	7.1	Substance abuse	10.8	Arthritis	13.2	Asthma	14.4	Anxiety	14.3	Asthma	14.8	Hyperlipidaemia	15.5	Osteoporosis	15.9
8	Epilepsy	8.7	Cardiac arrhythmia	6.5	Diabetes mellitus	10.3	Diabetes mellitus	12.1	Obesity	13.9	Cardiac arrhythmia	13.5	COPD	12.9	Diabetes mellitus	15.4	Depression	15.1
9	Depression	8.7	Diabetes mellitus	6.2	Arthritis	8.0	Substance abuse	8.1	Cardiac arrhythmia	8.7	Obesity	12.8	Heart failure	12.7	Asthma	13.3	Diabetes mellitus	11.4
10	Hyperlipidaemia	8.7	Heart failure	5.9	Cardiac arrhythmia	6.4	Cardiac arrhythmia	7.6	Substance abuse	6.4	COPD	9.7	Anxiety	12.4	COPD	13.0	Anxiety	10.1

Sex-standardised prevalence (%) is listed to the right of each condition listed. Shading is as follows: cardiometabolic conditions, red; respiratory conditions, green; mental health conditions, yellow; neurological conditions, blue; and musculoskeletal conditions, purple.

COPD, chronic obstructive pulmonary disease.

**Fig 7:**
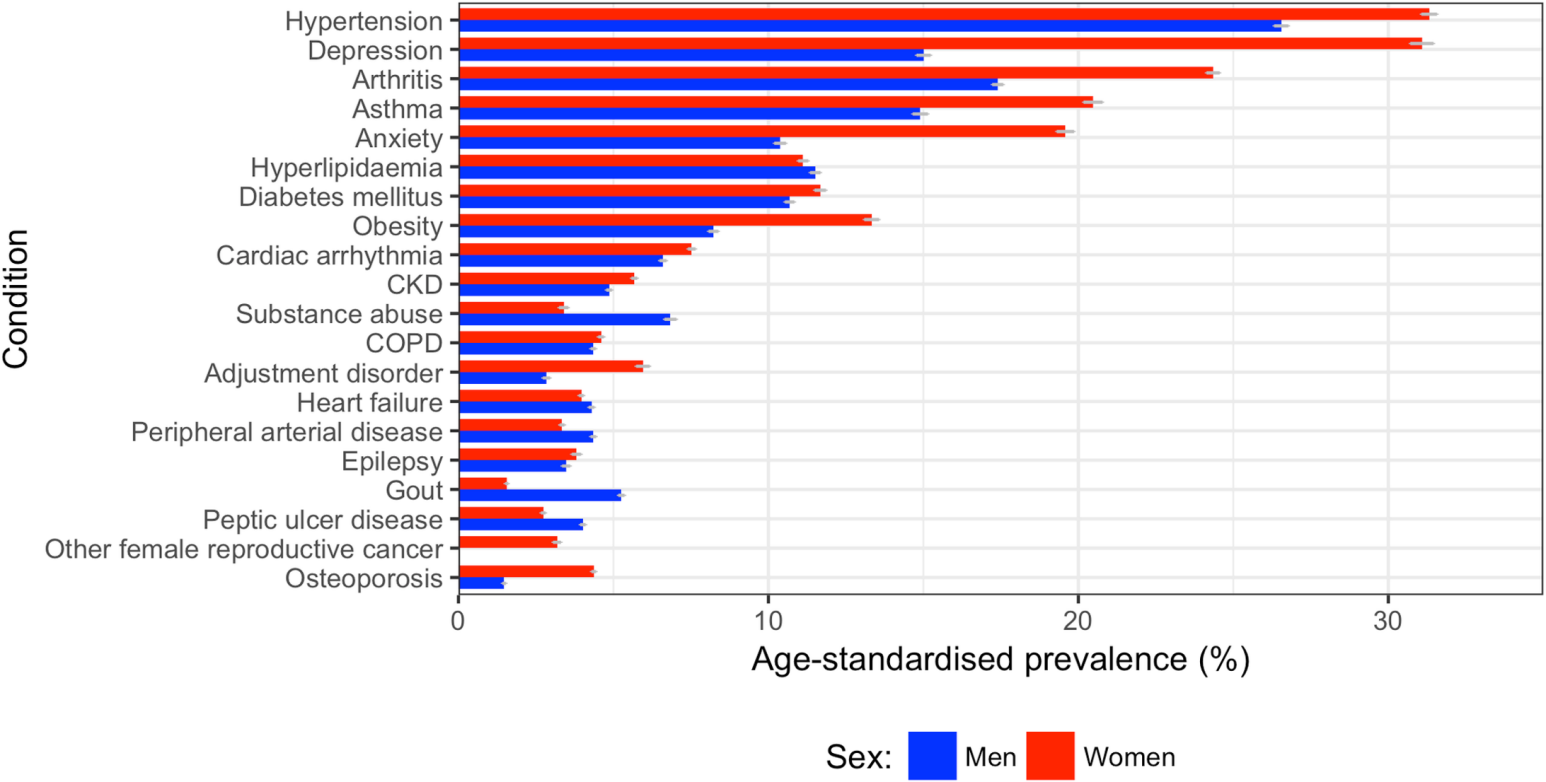
Age-standardised prevalence of comorbidities in women and men. The top 20 comorbidities ranked by sex-specific age-standardised prevalence between 2000 and 2014 are shown for men (blue) and women (red). CKD, chronic kidney disease; COPD, chronic obstructive pulmonary disease; PAD, peripheral arterial disease.

Women had higher age-standardised prevalence of most comorbidities compared to men (Fig 7). The top 5 conditions in women by age-standardised prevalence were hypertension (31.3% [95% CI 29.4%–35.0%]), depression (31.1% [28.3%–35.5%]), arthritis (24.3% [22.5%–28.0%]), asthma (20.5% [18.4%–24.3%]), and anxiety (19.6% [17.6%–23.3%]). In men, the top 5 conditions were hypertension (26.5% [95% CI 26.1%–27.9%]), arthritis (17.4% [16.5%–19.0%]), depression (15.0% [14.3%–16.5%]), asthma (14.9% [13.1%–17.3%]), and hyperlipidaemia (11.5% [10.7%–13.1%]). Out of the top 10 most prevalent comorbidities, ranked by overall age/sex-standardised prevalence for 2000–2014, the largest sex difference was for depression and anxiety: women’s age-standardised prevalence was 2.1 times that of men for depression (31.1% [95% CI 28.3%–35.5%] versus 15.0% [14.3%–16.5%]) and 1.9 times that of men for anxiety (19.6% [17.6%–23.3%] versus 10.4% [9.8%–11.8%]).

For all comorbidities in the top 10, patients in the most deprived fifth of SES had a higher prevalence than those in the least deprived fifth (S6 Fig). The largest difference between the most and least deprived fifths in age/sex-standardised models for the top 10 comorbidities was for depression, with a prevalence 46% higher in the most deprived fifth compared to the least deprived fifth (38.4% [95% CI 31.2%–62.0%] versus 26.3% [23.1%–34.5%]), followed by diabetes, with a prevalence 45% higher in the most deprived fifth compared to the least deprived fifth (22.6% [21.2%–43.4%] versus 15.6% [14.3%–22.7%]). The ranking of the most common comorbidities across the five SES groups was similar; a combination of hypertension, hyperlipidaemia, arthritis, depression, cardiac arrhythmia, and/or anxiety made up the top 5 comorbidities across each fifth of deprivation index.

## Discussion

In a representative population of 4.2 million UK adults, we identified 229,205 incident cases of non-fatal CVD, and measured the prevalence of 56 major comorbidities prior to CVD diagnosis. We observed that while age/sex-standardised incidence of non-fatal CVD decreased by a third between 2000 and 2014, the burden of multimorbidity and comorbidity increased. Although part of the increase was accounted for by an ageing population, even in age/sex-standardised analyses, the prevalence of having 5 or more comorbidities increased by 4 times, from 6.3% to 24.3%, between 2000 and 2014. The patterns of comorbidities differed substantially by age, sex, and SES. On average, older patients, women, and socioeconomically deprived groups had higher numbers of comorbidities, but the type of comorbidities differed by age and sex.

Most of the increased burden of comorbidity was in concordant cardiometabolic comorbidities such as hypertension, diabetes, and obesity, which are known to cluster together in the general population [30]. However, there was also a large burden of comorbidities that were discordant with CVD, such as arthritis, asthma, and mental illness (depression and anxiety), which are not perceived to share pathophysiological pathways [31]. On average, in the whole population of incident CVD cases, 4 out of the 10 top comorbidities were non-cardiometabolic.

The high prevalence of discordant comorbidities, such as arthritis, might be due to co-occurrence of common diseases in older populations. However, this does not explain the high prevalence of conditions such as mental illnesses, which were very common in younger patients. For instance, in those aged 40 to 49 years and 50 to 59 years, depression was ranked the second and third most common comorbidity, respectively, and affected about a quarter of these patients. Indirect comparisons with reports from the general UK population suggest that there is an excess of such conditions in those with incident CVD [32,33]. Consistent with our findings, a recent study using the CPRD showed that both prevalent and newly diagnosed depression was associated with cardiac, cerebrovascular, and peripheral arterial diseases [32]. However, this pattern is not confined to CVD; studies have shown that 25% of patients with a chronic medical condition have comorbid depression, and about double the prevalence or odds of depression compared to those without any chronic conditions [33]. The high prevalence of discordant comorbidities in patients with CVD suggests that the co-occurrence of some of them might not be due to chance alone: perhaps common aetiologies or shared risk factors, such as social stress and inflammation [23,34], could play a role in developing multimorbidity and influencing the specific comorbidity pattern in patients with CVD.

Our study furthers previous reports of the relationship between age and prevalence of multimorbidity [23]. In particular, we show that CVD patients have a very high prevalence of cardiometabolic comorbidity, with the majority of the top 10 comorbidities in all age groups being cardiometabolic conditions, a pattern that was also shown in patients with prevalent CVD in the US Medicare population [8]. The variation in comorbidity pattern across different age groups calls for research into age-specific comorbidity management in CVD, especially for mental illness in those aged 30–59 years, where about one-quarter of CVD patients were affected.

The increase in comorbid burden over time from 2000 to 2014 is partly explained by increased life expectancy and an ageing population [35]. However, in our analysis the relative increase in prevalence of multiple chronic conditions was similar when models were standardised for age. This suggests that age has not been the key driver of the increasing burden. Alternative explanations include changes in lifestyle factors such as smoking, physical inactivity, and diet, which have been identified as contributors to the development of chronic conditions and multimorbidity [36]. Health-system-related factors, such as changes in policies for better diagnosis, improved medical technologies, and more accessible health care, might have also contributed to the increased burden. Other explanations relate to patient factors, such as increased health literacy and health-seeking behaviour, leading to increased subsequent diagnoses.

Women with CVD had higher age-standardised prevalence of higher numbers of comorbidities, and of most individual comorbidities. The sex difference was maintained in most cardiometabolic conditions, despite the higher prevalence of cardiovascular risk factors in men at the time of diagnosis of CVD. There were particularly noticeable sex differences for depression and anxiety, where women had a prevalence about 1.5 times that of men. The sex difference in the prevalence of multimorbidity and physical/mental comorbidity has been identified in the general population [37]. The persistence of this sex difference in CVD patients suggests that although men are more likely to develop CVD, women are more likely to have a higher burden of comorbidities, including discordant comorbidities, that could potentially affect CVD management and outcomes.

Our study reinforces that CVD patients who are more socioeconomically deprived have a higher prevalence of more comorbidities. One-fifth of patients in the most deprived fifth had 5 or more comorbidities, compared to the same fraction of people in the least deprived 80% of the population having 1 comorbidity. The comorbidity pattern did not vary much between categories of deprivation for the most common comorbidities, suggesting that those who are more deprived are diagnosed with more of the most common comorbidities including hypertension, depression, and arthritis. The socioeconomic gradient of multimorbidity has been reported previously, including in a nationally representative primary care population of 1.7 million in Scotland [23], but, to our knowledge, the socioeconomic gradient with multimorbidity has not been examined in patients with CVD.

One strength of our study is the use of a large representative cohort across primary and secondary care in the UK that provides sufficient power to analyse overall prevalence of multimorbidity and individual comorbidities, as well as prevalence in subpopulations by age, sex, and SES. As such, the findings are likely to be of direct relevance to several other developed nations with a similar profile of CVD incidence. Our reported non-fatal CVD incidences over time are consistent with previous studies showing a one-third reduction in CVD over a similar period [38,39]. The longitudinal nature of our data has also allowed analysis of temporal trends, which have not been investigated previously for multimorbidity and comorbidity in CVD patients in the general population [9,40]. We also selected a population with incident diagnosis of CVD. This reduces the risk of diagnosis-time-dependent differences between CVD patients, such as survival time and severity of disease, and enables better comparisons of patients over time and by important features.

A limitation of using electronic health records is the dependence on recorded diagnoses, and hence lack of inclusion of undiagnosed cases or those with no contact with healthcare services, which could lead to underestimation of actual prevalences in the general population. Some of the patterns we see over time could be an artefact of recording bias or incentives, such as the QOF, which was introduced in 2004 [18]. In our study, QOF conditions did not seem to consistently peak in prevalence around 2004, nor did QOF conditions increase after 2004 when compared to non-QOF conditions, which would be expected if QOF incentives were a major driver of increased recorded diagnoses. Furthermore, there has been much investigation into the validity of diagnoses recorded by general practitioners in CPRD for a range of comorbidities, and the average positive predictive value is 89%, with 92% completeness when compared to national registries [41]. In addition, from a policy perspective, diagnoses recorded in health records are likely to be of higher relevance than routine population screening, because the former remain the basis for service planning and payment.

There is no common framework for selecting conditions for defining multimorbidity, and we used 3 different sources to collate a list of clinically relevant and common chronic conditions. This list includes the CVD risk factors hypertension, hyperlipidaemia, and obesity, which a large number of CVD patients develop before incident CVD. Our prevalences over time show that the increasing multimorbidity burden is unlikely to be attributable to these 3 conditions alone; the proportion of people who had 5 or more comorbidities increased 4-fold between 2000 and 2014, and the proportion with hypertension only increased by 11.9% between 2000 and 2014, which was one of the lower percentage increases amongst the top 10 most prevalent comorbidities.

Our descriptive study of 56 prevalent comorbidities in incident CVD is important in informing policy and research to guide clinical care. Multimorbidity has already been shown to associate with higher healthcare use, increased disability and mortality, and lower quality of life [35], and is associated with SES, age, and mental illness [23] in the general population. Our work quantifies the high and substantially increasing number of comorbidities in patients at the time of first CVD diagnosis, and also highlights the spectrum of disorders in the comorbidity burden, with 4 of the top 10 most prevalent conditions being non-cardiometabolic. These findings emphasise the need for more integrated models of care and challenges guidelines to better address the issue of management in the presence of several types of comorbidities. Future research investigating the role of stratified management of patients with different types of comorbidities is needed.

The current paradigm in CVD practice and policy is single-disease focused, where each cardiovascular condition is considered separately, with little reference to other conditions that potentially affect CVD prevention, risk stratification, and treatment. Although this approach may work well for related conditions, such as hypertension, heart failure, and diabetes, it does not account for discordant comorbidities. Some studies have suggested that focusing on specific problems or clusters, instead of clinical management of specific diseases, may be more effective for patients with multimorbidity [42]. Our study shows that there is a large burden of non-cardiometabolic comorbidity in CVD patients, including depression, anxiety, and asthma, which were all in the top 10 comorbidities out of the 56 comorbidities we studied. The burden and complexity of multimorbidity is increasing in CVD, and practice and policy must change towards more comprehensive CVD and multimorbidity care. In particular, studies that assess the impact of interventions in subgroups of patients with comorbidities could inform a more integrated approach to chronic disease management.

In conclusion, we found that the burden of multimorbidity and comorbidity in incident non-fatal CVD increased over time from 2000 to 2014. On average, older patients, women, and socially deprived groups had higher numbers of comorbidities, but the type of comorbidities varied by age and sex. Cardiometabolic conditions contributed substantially to the burden, but 4 out of the 10 top comorbidities were non-cardiometabolic. Our research shows that the high and substantially growing numbers of comorbidities require a reassessment of the current single-disease paradigm in CVD research and practice.

## Supporting information

S1 Fig Frequency of patients with incident CVD between 2000 and 2014 by age group (years). (TIF)

S2 Fig Annual age-standardised incidence of CVD, IHD, and stroke/TIA in women and men. (TIF)

S3 Fig Crude prevalence of numbers of comorbidities by age group (years). (TIF)

S4 Fig Age-standardised prevalence of number of comorbidities in women and men with incident CVD. (TIF)

S5 Fig Comparing QOF and non-QOF conditions in the 10 most common comorbidities by annual prevalence in patients with incident CVD between 2000 and 2014. (TIF)

S6 Fig Age/sex-standardised prevalence of comorbidities by SES group. (TIF)

S1 Table ICD-10 diagnostic codes for CVD and the 56 conditions included as comorbidities and in count of numbers of conditions. (DOCX)

S2 Table Age of male and female CVD patients at time of diagnosis each calendar year. (DOCX)

S3 Table Prevalence of number of conditions. (DOCX)

S4 Table Prevalence of specific comorbidities. (DOCX)

S1 Text Study protocol. (DOCX)

S2 Text RECORD guidelines. (DOCX)

## Abbreviations

BMI: body mass index
CPRD: Clinical Practice Research Datalink
CVD: cardiovascular disease
DBP: diastolic blood pressure
HES: Hospital Episode Statistics
IHD: ischaemic heart disease
IMD: Index of Multiple Deprivation
QOF: Quality and Outcomes Framework
SBP: systolic blood pressure
SES: socioeconomic status
TIA: transient ischaemic attack
