# Supplementary figures and images for: Patterns and temporal trends of comorbidity among adult patients with incident cardiovascular disease in the UK between 2000 and 2014: A population-based cohort study
Source: PLoS Med. 2018 Mar 6;15(3):e1002513. doi: 10.1371/journal.pmed.1002513 (PMC5839540; doi:10.1371/journal.pmed.1002513)

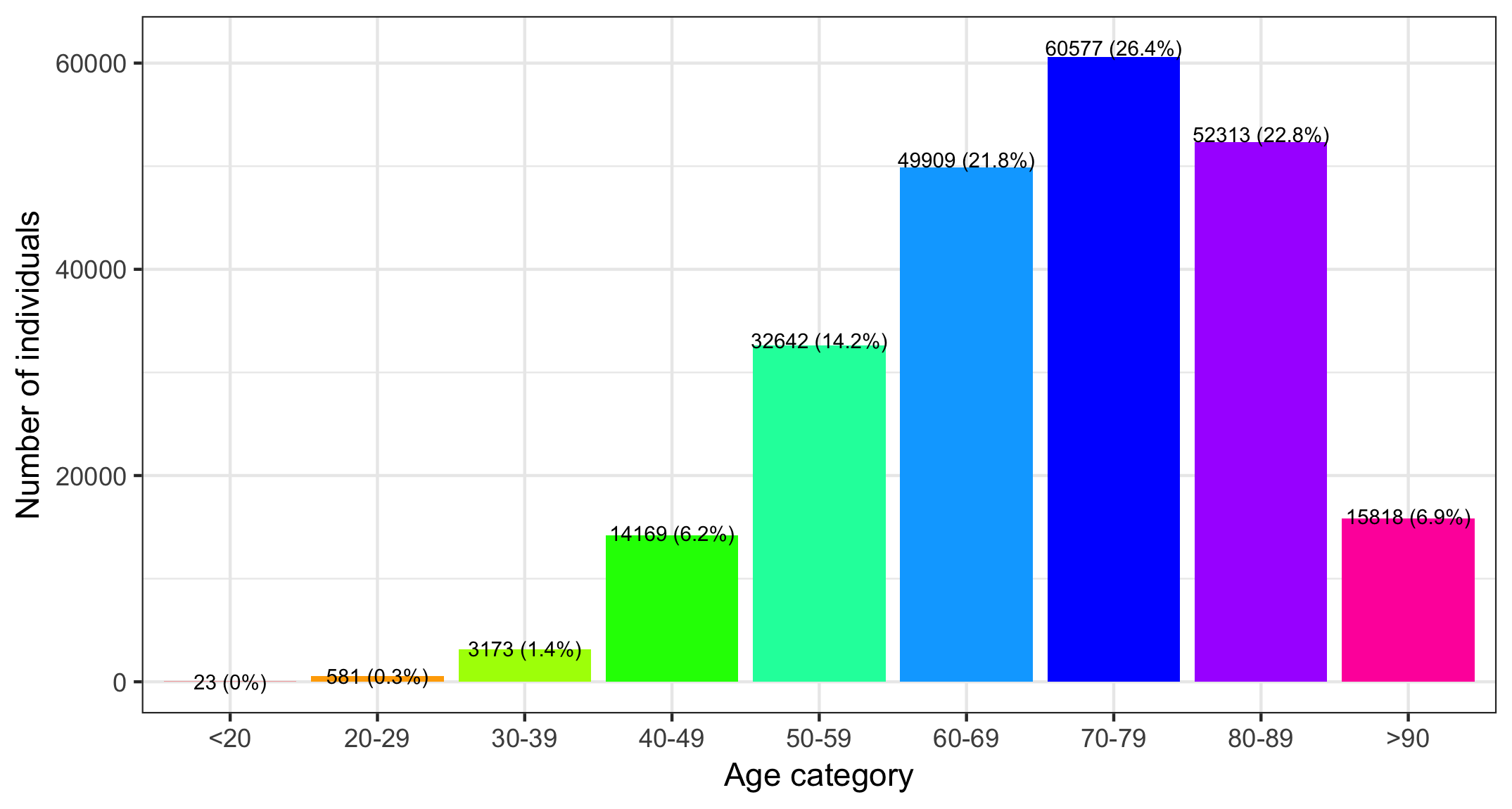

Supplement: S1 Fig — (TIF) [file pmed.1002513.s001.tif]

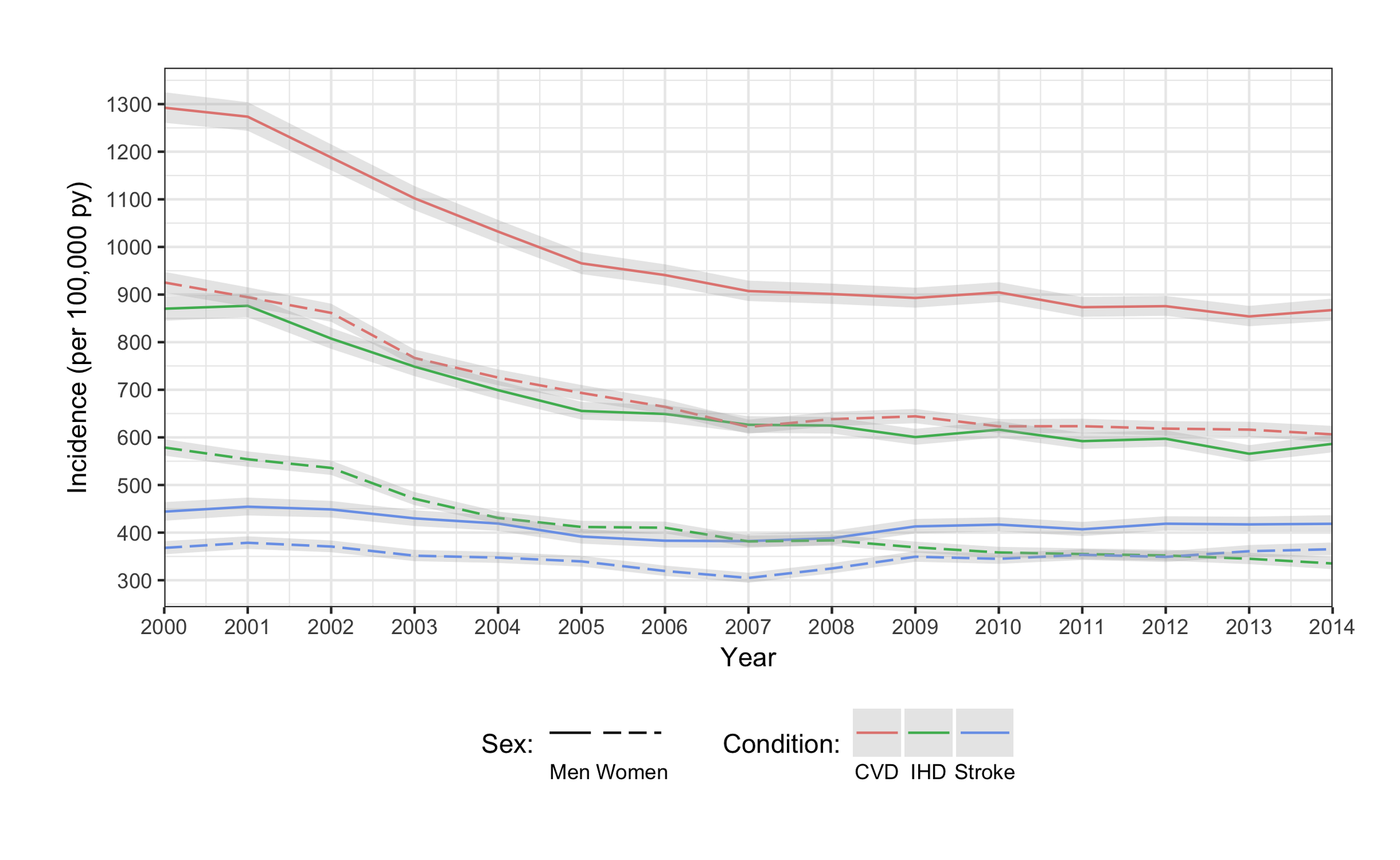

Supplement: S2 Fig — (TIF) [file pmed.1002513.s002.tif]

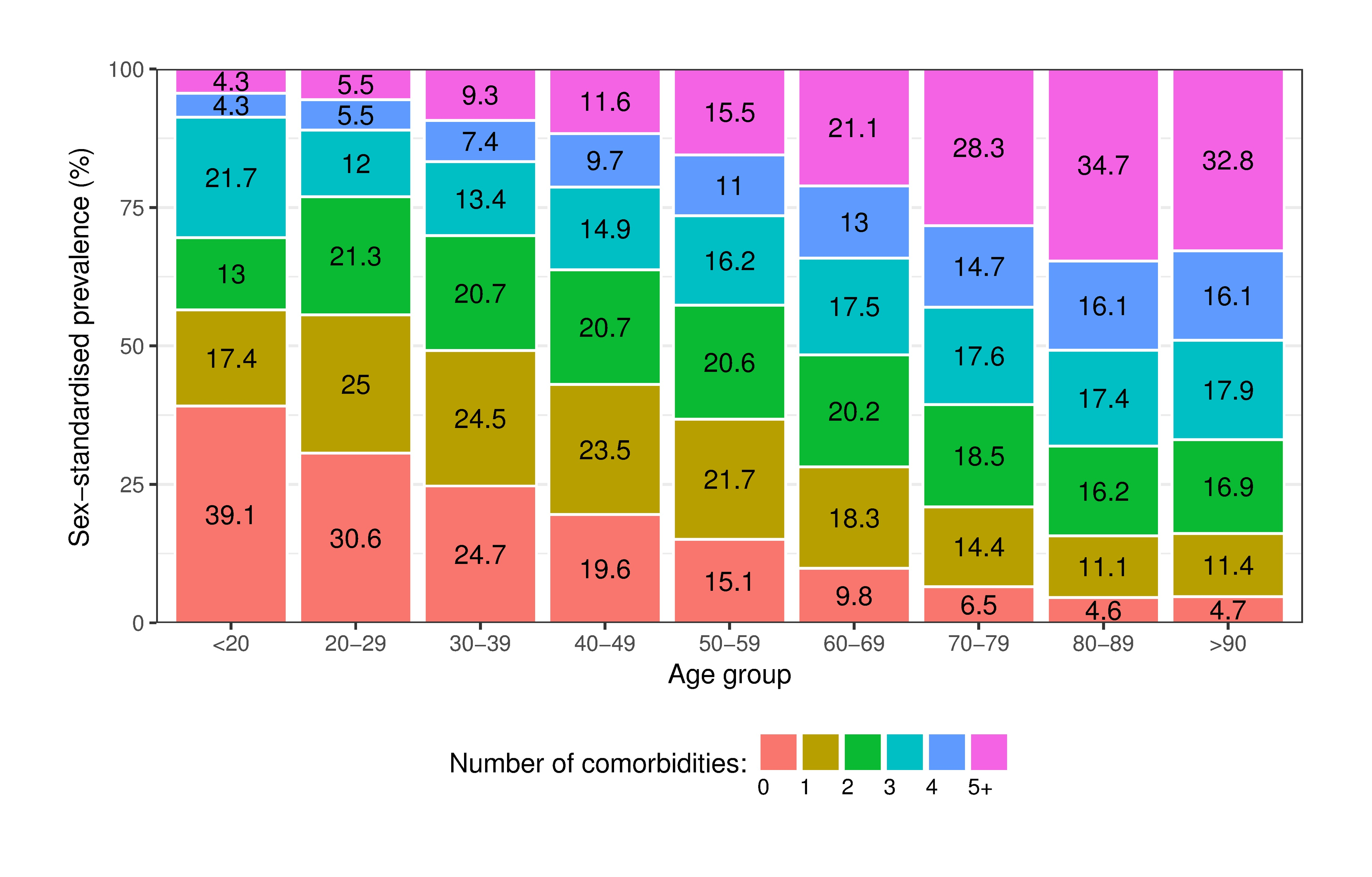

Supplement: S3 Fig — (TIF) [file pmed.1002513.s003.tif]

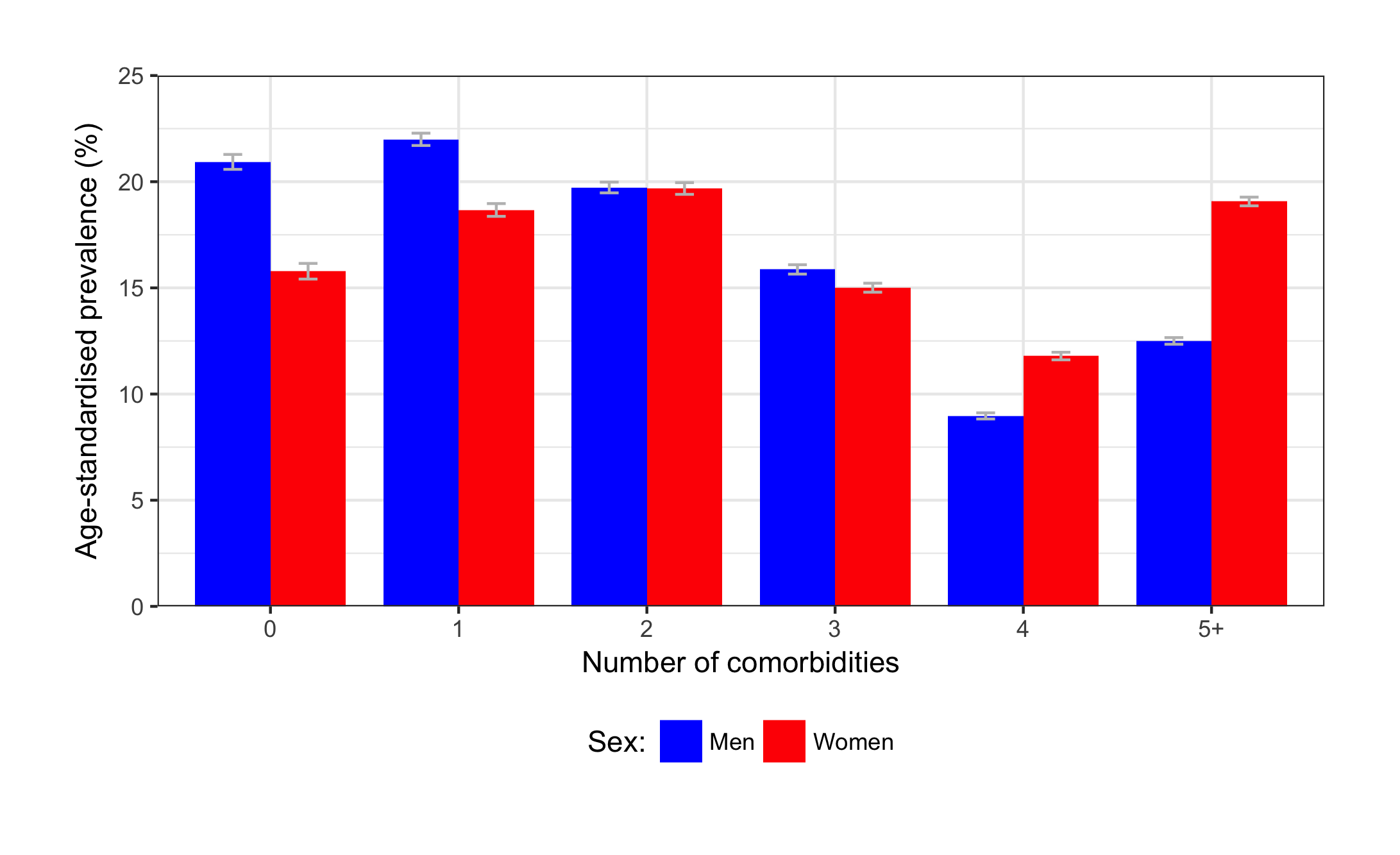

Supplement: S4 Fig — (TIF) [file pmed.1002513.s004.tif]

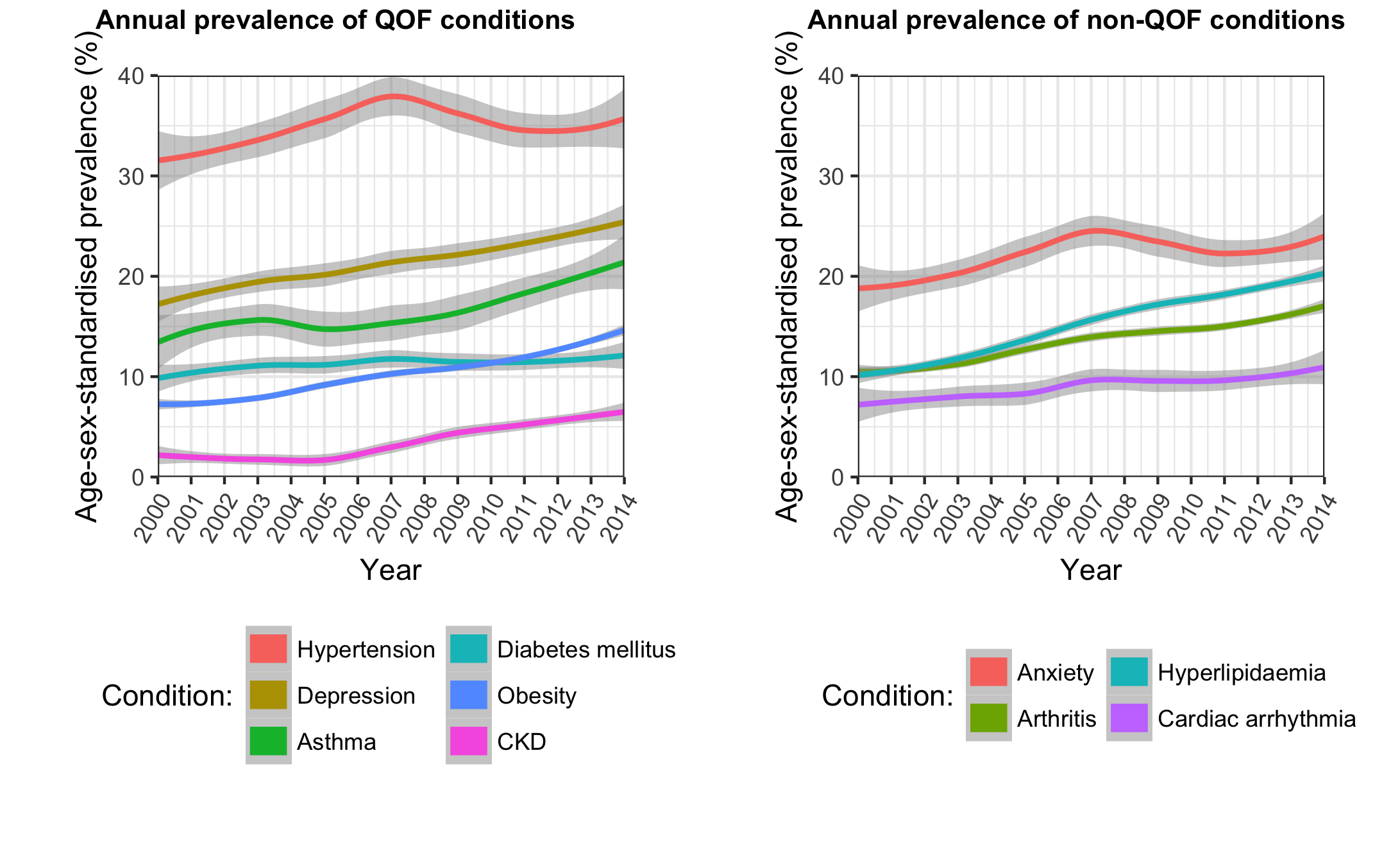

Supplement: S5 Fig — (TIF) [file pmed.1002513.s005.tif]

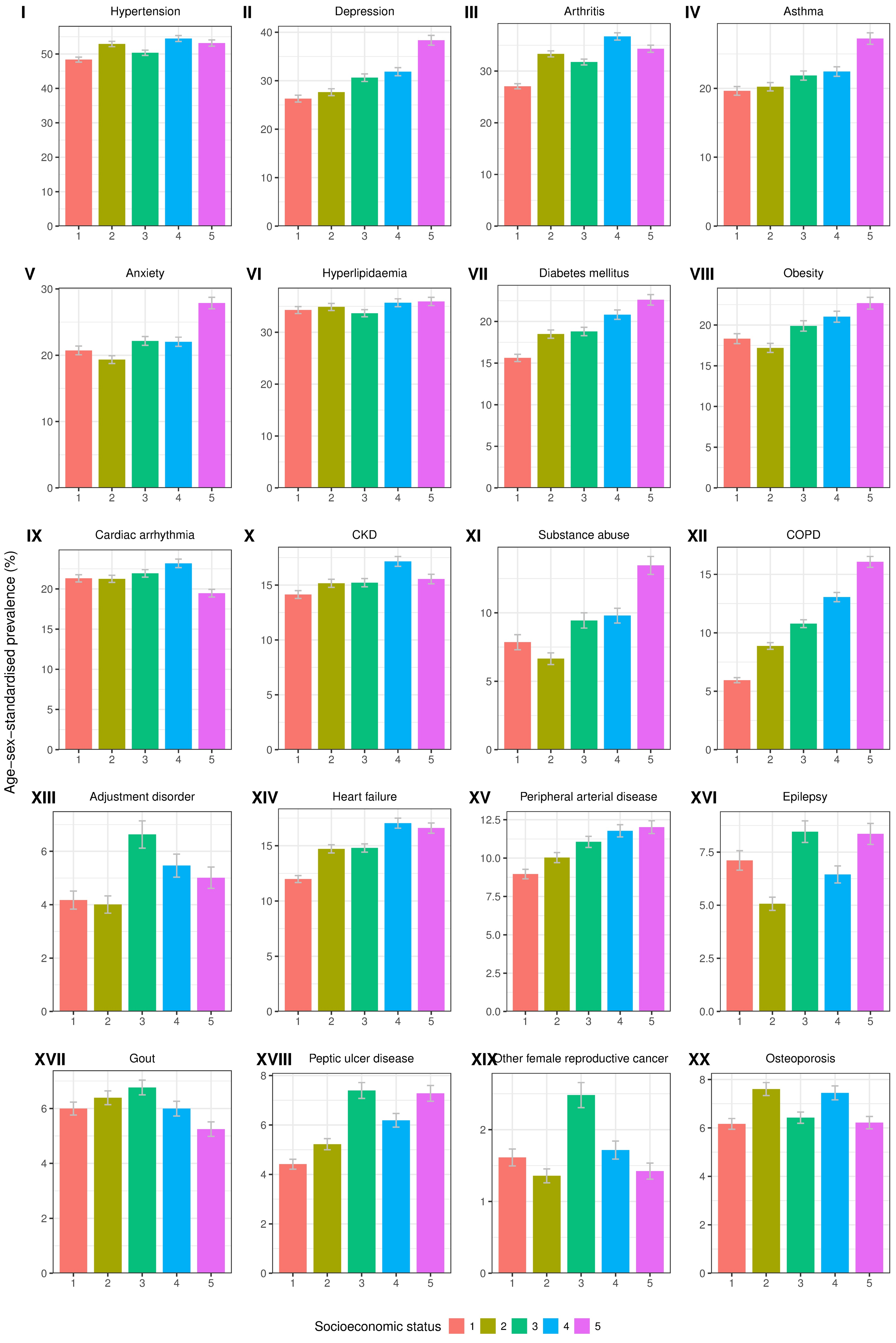

Supplement: S6 Fig — (TIF) [file pmed.1002513.s006.tif]
