# Supplementary material for: Patterns and temporal trends of comorbidity among adult patients with incident cardiovascular disease in the UK between 2000 and 2014: A population-based cohort study
Source: PLoS Med. 2018 Mar 6;15(3):e1002513. doi: 10.1371/journal.pmed.1002513 (PMC5839540; doi:10.1371/journal.pmed.1002513)
Supplement: S1 Table — (DOCX) [file pmed.1002513.s007.docx]

| **Condition** | **ICD code** | **Description** |
| --- | --- | --- |
| Adjustment disorder | F43 | Reaction to severe stress, and adjustment disorders |
| Affective disorder | F39 | Unspecified mood [affective] disorder |
| Affective disorder | F38 | Other mood [affective] disorders |
| Affective disorder | F34 | Persistent mood [affective] disorders |
| Anxiety | F41 | Other anxiety disorders |
| Anxiety | F40 | Phobic anxiety disorders |
| Arthritis | M48.99 | Spondylopathy, unspecified, site unspecified |
| Arthritis | M48.98 | Spondylopathy, unspecified, sacral and sacrococcygeal region |
| Arthritis | M48.97 | Spondylopathy, unspecified, lumbosacral region |
| Arthritis | M48.96 | Spondylopathy, unspecified, lumbar region |
| Arthritis | M48.95 | Spondylopathy, unspecified, thoracolumbar region |
| Arthritis | M48.94 | Spondylopathy, unspecified, thoracic region |
| Arthritis | M48.93 | Spondylopathy, unspecified, cervicothoracic region |
| Arthritis | M48.92 | Spondylopathy, unspecified, cervical region |
| Arthritis | M48.91 | Spondylopathy, unspecified, occipito-atlanto-axial region |
| Arthritis | M48.90 | Spondylopathy, unspecified, multiple sites in spine |
| Arthritis | M48.9 | Spondylopathy, unspecified |
| Arthritis | M48.89 | Other specified spondylopathies, site unspecified |
| Arthritis | M48.88 | Other specified spondylopathies, sacral and sacrococcygeal region |
| Arthritis | M48.87 | Other specified spondylopathies, lumbosacral region |
| Arthritis | M48.86 | Other specified spondylopathies, lumbar region |
| Arthritis | M48.85 | Other specified spondylopathies, thoracolumbar region |
| Arthritis | M48.84 | Other specified spondylopathies, thoracic region |
| Arthritis | M48.83 | Other specified spondylopathies, cervicothoracic region |
| Arthritis | M48.82 | Other specified spondylopathies, cervical region |
| Arthritis | M48.81 | Other specified spondylopathies, occipito-atlanto-axial region |
| Arthritis | M48.80 | Other specified spondylopathies, multiple sites in spine |
| Arthritis | M48.8 | Other specified spondylopathies |
| Arthritis | M48.09 | Spinal stenosis, site unspecified |
| Arthritis | M48.08 | Spinal stenosis, sacral and sacrococcygeal region |
| Arthritis | M48.07 | Spinal stenosis, lumbosacral region |
| Arthritis | M48.06 | Spinal stenosis, lumbar region |
| Arthritis | M48.05 | Spinal stenosis, thoracolumbar region |
| Arthritis | M48.04 | Spinal stenosis, thoracic region |
| Arthritis | M48.03 | Spinal stenosis, cervicothoracic region |
| Arthritis | M48.02 | Spinal stenosis, cervical region |
| Arthritis | M48.01 | Spinal stenosis, occipito-atlanto-axial region |
| Arthritis | M48.00 | Spinal stenosis, multiple sites in spine |
| Arthritis | M48.0 | Spinal stenosis |
| Arthritis | M48 | Other spondylopathies |
| Arthritis | M479 | Spondylosis, unspecified |
| Arthritis | M478 | Other spondylosis |
| Arthritis | M472 | Other spondylosis with radiculopathy |
| Arthritis | M471 | Other spondylosis with myelopathy |
| Arthritis | M47.99 | Spondylosis, unspecified, site unspecified |
| Arthritis | M47.98 | Spondylosis, unspecified, sacral and sacrococcygeal region |
| Arthritis | M47.97 | Spondylosis, unspecified, lumbosacral region |
| Arthritis | M47.96 | Spondylosis, unspecified, lumbar region |
| Arthritis | M47.95 | Spondylosis, unspecified, thoracolumbar region |
| Arthritis | M47.94 | Spondylosis, unspecified, thoracic region |
| Arthritis | M47.93 | Spondylosis, unspecified, cervicothoracic region |
| Arthritis | M47.92 | Spondylosis, unspecified, cervical region |
| Arthritis | M47.91 | Spondylosis, unspecified, occipito-atlanto-axial region |
| Arthritis | M47.90 | Spondylosis, unspecified, multiple sites in spine |
| Arthritis | M47.9 | Spondylosis, unspecified |
| Arthritis | M47.89 | Other spondylosis, site unspecified |
| Arthritis | M47.88 | Other spondylosis, sacral and sacrococcygeal region |
| Arthritis | M47.87 | Other spondylosis, lumbosacral region |
| Arthritis | M47.86 | Other spondylosis, lumbar region |
| Arthritis | M47.85 | Other spondylosis, thoracolumbar region |
| Arthritis | M47.84 | Other spondylosis, thoracic region |
| Arthritis | M47.83 | Other spondylosis, cervicothoracic region |
| Arthritis | M47.82 | Other spondylosis, cervical region |
| Arthritis | M47.81 | Other spondylosis, occipito-atlanto-axial region |
| Arthritis | M47.80 | Other spondylosis, multiple sites in spine |
| Arthritis | M47.8 | Other spondylosis |
| Arthritis | M47.29 | Other spondylosis with radiculopathy, site unspecified |
| Arthritis | M47.28 | Other spondylosis with radiculopathy, sacral and sacrococcygeal region |
| Arthritis | M47.27 | Other spondylosis with radiculopathy, lumbosacral region |
| Arthritis | M47.26 | Other spondylosis with radiculopathy, lumbar region |
| Arthritis | M47.25 | Other spondylosis with radiculopathy, thoracolumbar region |
| Arthritis | M47.24 | Other spondylosis with radiculopathy, thoracic region |
| Arthritis | M47.23 | Other spondylosis with radiculopathy, cervicothoracic region |
| Arthritis | M47.22 | Other spondylosis with radiculopathy, cervical region |
| Arthritis | M47.21 | Other spondylosis with radiculopathy, occipito-atlanto-axial region |
| Arthritis | M47.20 | Other spondylosis with radiculopathy, multiple sites in spine |
| Arthritis | M47.2 | Other spondylosis with radiculopathy |
| Arthritis | M47.19 | Other spondylosis with myelopathy, site unspecified |
| Arthritis | M47.18 | Other spondylosis with myelopathy, sacral and sacrococcygeal region |
| Arthritis | M47.17 | Other spondylosis with myelopathy, lumbosacral region |
| Arthritis | M47.16 | Other spondylosis with myelopathy, lumbar region |
| Arthritis | M47.15 | Other spondylosis with myelopathy, thoracolumbar region |
| Arthritis | M47.14 | Other spondylosis with myelopathy, thoracic region |
| Arthritis | M47.13 | Other spondylosis with myelopathy, cervicothoracic region |
| Arthritis | M47.12 | Other spondylosis with myelopathy, cervical region |
| Arthritis | M47.11 | Other spondylosis with myelopathy, occipito-atlanto-axial region |
| Arthritis | M47.10 | Other spondylosis with myelopathy, multiple sites in spine |
| Arthritis | M47.1 | Other spondylosis with myelopathy |
| Arthritis | M47 | Spondylosis |
| Arthritis | M46.18 | Sacroiliitis, not elsewhere classified, sacral and sacrococcygeal region |
| Arthritis | M46.17 | Sacroiliitis, not elsewhere classified, lumbosacral region |
| Arthritis | M46.1 | Sacroiliitis, not elsewhere classified |
| Arthritis | M43.99 | Deforming dorsopathy, unspecified, site unspecified |
| Arthritis | M43.98 | Deforming dorsopathy, unspecified, sacral and sacrococcygeal region |
| Arthritis | M43.97 | Deforming dorsopathy, unspecified, lumbosacral region |
| Arthritis | M43.96 | Deforming dorsopathy, unspecified, lumbar region |
| Arthritis | M43.95 | Deforming dorsopathy, unspecified, thoracolumbar region |
| Arthritis | M43.94 | Deforming dorsopathy, unspecified, thoracic region |
| Arthritis | M43.93 | Deforming dorsopathy, unspecified, cervicothoracic region |
| Arthritis | M43.92 | Deforming dorsopathy, unspecified, cervical region |
| Arthritis | M43.91 | Deforming dorsopathy, unspecified, occipito-atlanto-axial region |
| Arthritis | M43.90 | Deforming dorsopathy, unspecified, multiple sites in spine |
| Arthritis | M43.9 | Deforming dorsopathy, unspecified |
| Arthritis | M43.89 | Other specified deforming dorsopathies, site unspecified |
| Arthritis | M43.88 | Other specified deforming dorsopathies, sacral and sacrococcygeal region |
| Arthritis | M43.87 | Other specified deforming dorsopathies, lumbosacral region |
| Arthritis | M43.86 | Other specified deforming dorsopathies, lumbar region |
| Arthritis | M43.85 | Other specified deforming dorsopathies, thoracolumbar region |
| Arthritis | M43.84 | Other specified deforming dorsopathies, thoracic region |
| Arthritis | M43.83 | Other specified deforming dorsopathies, cervicothoracic region |
| Arthritis | M43.82 | Other specified deforming dorsopathies, cervical region |
| Arthritis | M43.81 | Other specified deforming dorsopathies, occipito-atlanto-axial region |
| Arthritis | M43.80 | Other specified deforming dorsopathies, multiple sites in spine |
| Arthritis | M43.8 | Other specified deforming dorsopathies |
| Arthritis | M43.09 | Spondylolysis, site unspecified |
| Arthritis | M43.08 | Spondylolysis, sacral and sacrococcygeal region |
| Arthritis | M43.07 | Spondylolysis, lumbosacral region |
| Arthritis | M43.06 | Spondylolysis, lumbar region |
| Arthritis | M43.05 | Spondylolysis, thoracolumbar region |
| Arthritis | M43.04 | Spondylolysis, thoracic region |
| Arthritis | M43.03 | Spondylolysis, cervicothoracic region |
| Arthritis | M43.02 | Spondylolysis, cervical region |
| Arthritis | M43.01 | Spondylolysis, occipito-atlanto-axial region |
| Arthritis | M43.00 | Spondylolysis, multiple sites in spine |
| Arthritis | M43.0 | Spondylolysis |
| Arthritis | M43 | Other deforming dorsopathies |
| Arthritis | M25.99 | Joint disorder, unspecified, site unspecified |
| Arthritis | M25.98 | Joint disorder, unspecified, other site |
| Arthritis | M25.97 | Joint disorder, unspecified, ankle and foot |
| Arthritis | M25.96 | Joint disorder, unspecified, lower leg |
| Arthritis | M25.95 | Joint disorder, unspecified, pelvic region and thigh |
| Arthritis | M25.94 | Joint disorder, unspecified, hand |
| Arthritis | M25.93 | Joint disorder, unspecified, forearm |
| Arthritis | M25.92 | Joint disorder, unspecified, upper arm |
| Arthritis | M25.91 | Joint disorder, unspecified, shoulder region |
| Arthritis | M25.90 | Joint disorder, unspecified, multiple sites |
| Arthritis | M25.9 | Joint disorder, unspecified |
| Arthritis | M25.89 | Other specified joint disorders, site unspecified |
| Arthritis | M25.88 | Other specified joint disorders, other site |
| Arthritis | M25.87 | Other specified joint disorders, ankle and foot |
| Arthritis | M25.86 | Other specified joint disorders, lower leg |
| Arthritis | M25.85 | Other specified joint disorders, pelvic region and thigh |
| Arthritis | M25.84 | Other specified joint disorders, hand |
| Arthritis | M25.83 | Other specified joint disorders, forearm |
| Arthritis | M25.82 | Other specified joint disorders, upper arm |
| Arthritis | M25.81 | Other specified joint disorders, shoulder region |
| Arthritis | M25.80 | Other specified joint disorders, multiple sites |
| Arthritis | M25.8 | Other specified joint disorders |
| Arthritis | M25.79 | Osteophyte, site unspecified |
| Arthritis | M25.78 | Osteophyte, other site |
| Arthritis | M25.77 | Osteophyte, ankle and foot |
| Arthritis | M25.76 | Osteophyte, lower leg |
| Arthritis | M25.75 | Osteophyte, pelvic region and thigh |
| Arthritis | M25.74 | Osteophyte, hand |
| Arthritis | M25.73 | Osteophyte, forearm |
| Arthritis | M25.72 | Osteophyte, upper arm |
| Arthritis | M25.71 | Osteophyte, shoulder region |
| Arthritis | M25.70 | Osteophyte, multiple sites |
| Arthritis | M25.7 | Osteophyte |
| Arthritis | M25.69 | Stiffness of joint, not elsewhere classified, site unspecified |
| Arthritis | M25.68 | Stiffness of joint, not elsewhere classified, other site |
| Arthritis | M25.67 | Stiffness of joint, not elsewhere classified, ankle and foot |
| Arthritis | M25.66 | Stiffness of joint, not elsewhere classified, lower leg |
| Arthritis | M25.65 | Stiffness of joint, not elsewhere classified, pelvic region and thigh |
| Arthritis | M25.64 | Stiffness of joint, not elsewhere classified, hand |
| Arthritis | M25.63 | Stiffness of joint, not elsewhere classified, forearm |
| Arthritis | M25.62 | Stiffness of joint, not elsewhere classified, upper arm |
| Arthritis | M25.61 | Stiffness of joint, not elsewhere classified, shoulder region |
| Arthritis | M25.60 | Stiffness of joint, not elsewhere classified, multiple sites |
| Arthritis | M25.6 | Stiffness of joint, not elsewhere classified |
| Arthritis | M25.59 | Pain in joint, site unspecified |
| Arthritis | M25.58 | Pain in joint, other site |
| Arthritis | M25.57 | Pain in joint, ankle and foot |
| Arthritis | M25.56 | Pain in joint, lower leg |
| Arthritis | M25.55 | Pain in joint, pelvic region and thigh |
| Arthritis | M25.54 | Pain in joint, hand |
| Arthritis | M25.53 | Pain in joint, forearm |
| Arthritis | M25.52 | Pain in joint, upper arm |
| Arthritis | M25.51 | Pain in joint, shoulder region |
| Arthritis | M25.50 | Pain in joint, multiple sites |
| Arthritis | M25.5 | Pain in joint |
| Arthritis | M25 | Other joint disorders, not elsewhere classified |
| Arthritis | M19.99 | Arthrosis, unspecified, site unspecified |
| Arthritis | M19.98 | Arthrosis, unspecified, other site |
| Arthritis | M19.97 | Arthrosis, unspecified, ankle and foot |
| Arthritis | M19.96 | Arthrosis, unspecified, lower leg |
| Arthritis | M19.95 | Arthrosis, unspecified, pelvic region and thigh |
| Arthritis | M19.94 | Arthrosis, unspecified, hand |
| Arthritis | M19.93 | Arthrosis, unspecified, forearm |
| Arthritis | M19.92 | Arthrosis, unspecified, upper arm |
| Arthritis | M19.91 | Arthrosis, unspecified, shoulder region |
| Arthritis | M19.90 | Arthrosis, unspecified, multiple sites |
| Arthritis | M19.9 | Arthrosis, unspecified |
| Arthritis | M19.89 | Other specified arthrosis, site unspecified |
| Arthritis | M19.88 | Other specified arthrosis, other site |
| Arthritis | M19.87 | Other specified arthrosis, ankle and foot |
| Arthritis | M19.86 | Other specified arthrosis, lower leg |
| Arthritis | M19.85 | Other specified arthrosis, pelvic region and thigh |
| Arthritis | M19.84 | Other specified arthrosis, hand |
| Arthritis | M19.83 | Other specified arthrosis, forearm |
| Arthritis | M19.82 | Other specified arthrosis, upper arm |
| Arthritis | M19.81 | Other specified arthrosis, shoulder region |
| Arthritis | M19.80 | Other specified arthrosis, multiple sites |
| Arthritis | M19.8 | Other specified arthrosis |
| Arthritis | M19.29 | Other secondary arthrosis, site unspecified |
| Arthritis | M19.28 | Other secondary arthrosis, other site |
| Arthritis | M19.27 | Other secondary arthrosis, ankle and foot |
| Arthritis | M19.26 | Other secondary arthrosis, lower leg |
| Arthritis | M19.25 | Other secondary arthrosis, pelvic region and thigh |
| Arthritis | M19.24 | Other secondary arthrosis, hand |
| Arthritis | M19.23 | Other secondary arthrosis, forearm |
| Arthritis | M19.22 | Other secondary arthrosis, upper arm |
| Arthritis | M19.21 | Other secondary arthrosis, shoulder region |
| Arthritis | M19.20 | Other secondary arthrosis, multiple sites |
| Arthritis | M19.2 | Other secondary arthrosis |
| Arthritis | M19.19 | Post-traumatic arthrosis of other joints, site unspecified |
| Arthritis | M19.18 | Post-traumatic arthrosis of other joints, other site |
| Arthritis | M19.17 | Post-traumatic arthrosis of other joints, ankle and foot |
| Arthritis | M19.16 | Post-traumatic arthrosis of other joints, lower leg |
| Arthritis | M19.15 | Post-traumatic arthrosis of other joints, pelvic region and thigh |
| Arthritis | M19.14 | Post-traumatic arthrosis of other joints, hand |
| Arthritis | M19.13 | Post-traumatic arthrosis of other joints, forearm |
| Arthritis | M19.12 | Post-traumatic arthrosis of other joints, upper arm |
| Arthritis | M19.11 | Post-traumatic arthrosis of other joints, shoulder region |
| Arthritis | M19.10 | Post-traumatic arthrosis of other joints, multiple sites |
| Arthritis | M19.1 | Post-traumatic arthrosis of other joints |
| Arthritis | M19.09 | Primary arthrosis of other joints, site unspecified |
| Arthritis | M19.08 | Primary arthrosis of other joints, other site |
| Arthritis | M19.07 | Primary arthrosis of other joints, ankle and foot |
| Arthritis | M19.06 | Primary arthrosis of other joints, lower leg |
| Arthritis | M19.05 | Primary arthrosis of other joints, pelvic region and thigh |
| Arthritis | M19.04 | Primary arthrosis of other joints, hand |
| Arthritis | M19.03 | Primary arthrosis of other joints, forearm |
| Arthritis | M19.02 | Primary arthrosis of other joints, upper arm |
| Arthritis | M19.01 | Primary arthrosis of other joints, shoulder region |
| Arthritis | M19.00 | Primary arthrosis of other joints, multiple sites |
| Arthritis | M19.0 | Primary arthrosis of other joints |
| Arthritis | M19 | Other arthrosis |
| Arthritis | M18.9 | Arthrosis of first carpometacarpal joint, unspecified |
| Arthritis | M18.5 | Other secondary arthrosis of first carpometacarpal joint |
| Arthritis | M18.4 | Other secondary arthrosis of first carpometacarpal joints, bilateral |
| Arthritis | M18.3 | Other post-traumatic arthrosis of first carpometacarpal joint |
| Arthritis | M18.2 | Post-traumatic arthrosis of first carpometacarpal joints, bilateral |
| Arthritis | M18.1 | Other primary arthrosis of first carpometacarpal joint |
| Arthritis | M18.0 | Primary arthrosis of first carpometacarpal joints, bilateral |
| Arthritis | M18 | Arthrosis of first carpometacarpal joint |
| Arthritis | M17.9 | Gonarthrosis, unspecified |
| Arthritis | M17.5 | Other secondary gonarthrosis |
| Arthritis | M17.4 | Other secondary gonarthrosis, bilateral |
| Arthritis | M17.3 | Other post-traumatic gonarthrosis |
| Arthritis | M17.2 | Post-traumatic gonarthrosis, bilateral |
| Arthritis | M17.1 | Other primary gonarthrosis |
| Arthritis | M17.0 | Primary gonarthrosis, bilateral |
| Arthritis | M17 | Gonarthrosis [arthrosis of knee] |
| Arthritis | M16.9 | Coxarthrosis, unspecified |
| Arthritis | M16.7 | Other secondary coxarthrosis |
| Arthritis | M16.6 | Other secondary coxarthrosis, bilateral |
| Arthritis | M16.5 | Other post-traumatic coxarthrosis |
| Arthritis | M16.4 | Post-traumatic coxarthrosis, bilateral |
| Arthritis | M16.3 | Other dysplastic coxarthrosis |
| Arthritis | M16.2 | Coxarthrosis resulting from dysplasia, bilateral |
| Arthritis | M16.1 | Other primary coxarthrosis |
| Arthritis | M16.0 | Primary coxarthrosis, bilateral |
| Arthritis | M16 | Coxarthrosis [arthrosis of hip] |
| Arthritis | M15.9 | Polyarthrosis, unspecified |
| Arthritis | M15.8 | Other polyarthrosis |
| Arthritis | M15.4 | Erosive (osteo)arthrosis |
| Arthritis | M15.3 | Secondary multiple arthrosis |
| Arthritis | M15.2 | Bouchard's nodes (with arthropathy) |
| Arthritis | M15.1 | Heberden's nodes (with arthropathy) |
| Arthritis | M15.0 | Primary generalized (osteo)arthrosis |
| Arthritis | M15 | Polyarthrosis |
| Arthritis | M14.8 | Arthropathies in other specified diseases classified elsewhere |
| Arthritis | M14.6 | Neuropathic arthropathy |
| Arthritis | M14.5 | Arthropathies in other endocrine, nutritional and metabolic disorders |
| Arthritis | M14.4 | Arthropathy in amyloidosis (E85.-+) |
| Arthritis | M14.3 | Lipoid dermatoarthritis (E78.8+) |
| Arthritis | M14.2 | Diabetic arthropathy (E10-E14+ with common fourth character .6) |
| Arthritis | M14.1 | Crystal arthropathy in other metabolic disorders |
| Arthritis | M14 | Arthropathies in other diseases classified elsewhere |
| Arthritis | M13.99 | Arthritis, unspecified, site unspecified |
| Arthritis | M13.98 | Arthritis, unspecified, other site |
| Arthritis | M13.97 | Arthritis, unspecified, ankle and foot |
| Arthritis | M13.96 | Arthritis, unspecified, lower leg |
| Arthritis | M13.95 | Arthritis, unspecified, pelvic region and thigh |
| Arthritis | M13.94 | Arthritis, unspecified, hand |
| Arthritis | M13.93 | Arthritis, unspecified, forearm |
| Arthritis | M13.92 | Arthritis, unspecified, upper arm |
| Arthritis | M13.91 | Arthritis, unspecified, shoulder region |
| Arthritis | M13.90 | Arthritis, unspecified, multiple sites |
| Arthritis | M13.9 | Arthritis, unspecified |
| Arthritis | M13.89 | Other specified arthritis, site unspecified |
| Arthritis | M13.88 | Other specified arthritis, other site |
| Arthritis | M13.87 | Other specified arthritis, ankle and foot |
| Arthritis | M13.86 | Other specified arthritis, lower leg |
| Arthritis | M13.85 | Other specified arthritis, pelvic region and thigh |
| Arthritis | M13.84 | Other specified arthritis, hand |
| Arthritis | M13.83 | Other specified arthritis, forearm |
| Arthritis | M13.82 | Other specified arthritis, upper arm |
| Arthritis | M13.81 | Other specified arthritis, shoulder region |
| Arthritis | M13.80 | Other specified arthritis, multiple sites |
| Arthritis | M13.8 | Other specified arthritis |
| Arthritis | M13.19 | Monoarthritis, not elsewhere classified, site unspecified |
| Arthritis | M13.18 | Monoarthritis, not elsewhere classified, other site |
| Arthritis | M13.17 | Monoarthritis, not elsewhere classified, ankle and foot |
| Arthritis | M13.16 | Monoarthritis, not elsewhere classified, lower leg |
| Arthritis | M13.15 | Monoarthritis, not elsewhere classified, pelvic region and thigh |
| Arthritis | M13.14 | Monoarthritis, not elsewhere classified, hand |
| Arthritis | M13.13 | Monoarthritis, not elsewhere classified, forearm |
| Arthritis | M13.12 | Monoarthritis, not elsewhere classified, upper arm |
| Arthritis | M13.11 | Monoarthritis, not elsewhere classified, shoulder region |
| Arthritis | M13.10 | Monoarthritis, not elsewhere classified, multiple sites |
| Arthritis | M13.1 | Monoarthritis, not elsewhere classified |
| Arthritis | M13.09 | Polyarthritis, unspecified, site unspecified |
| Arthritis | M13.08 | Polyarthritis, unspecified, other site |
| Arthritis | M13.07 | Polyarthritis, unspecified, ankle and foot |
| Arthritis | M13.06 | Polyarthritis, unspecified, lower leg |
| Arthritis | M13.05 | Polyarthritis, unspecified, pelvic region and thigh |
| Arthritis | M13.04 | Polyarthritis, unspecified, hand |
| Arthritis | M13.03 | Polyarthritis, unspecified, forearm |
| Arthritis | M13.02 | Polyarthritis, unspecified, upper arm |
| Arthritis | M13.01 | Polyarthritis, unspecified, shoulder region |
| Arthritis | M13.00 | Polyarthritis, unspecified, multiple sites |
| Arthritis | M13.0 | Polyarthritis, unspecified |
| Arthritis | M13 | Other arthritis |
| Arthritis | M12.89 | Other specific arthropathies, not elsewhere classified, site unspecified |
| Arthritis | M12.88 | Other specific arthropathies, not elsewhere classified, other site |
| Arthritis | M12.87 | Other specific arthropathies, not elsewhere classified, ankle and foot |
| Arthritis | M12.86 | Other specific arthropathies, not elsewhere classified, lower leg |
| Arthritis | M12.85 | Other specific arthropathies, not elsewhere classified, pelvic region and thigh |
| Arthritis | M12.84 | Other specific arthropathies, not elsewhere classified, hand |
| Arthritis | M12.83 | Other specific arthropathies, not elsewhere classified, forearm |
| Arthritis | M12.82 | Other specific arthropathies, not elsewhere classified, upper arm |
| Arthritis | M12.81 | Other specific arthropathies, not elsewhere classified, shoulder region |
| Arthritis | M12.80 | Other specific arthropathies, not elsewhere classified, multiple sites |
| Arthritis | M12.8 | Other specific arthropathies, not elsewhere classified |
| Arthritis | M12.59 | Traumatic arthropathy, site unspecified |
| Arthritis | M12.58 | Traumatic arthropathy, other site |
| Arthritis | M12.57 | Traumatic arthropathy, ankle and foot |
| Arthritis | M12.56 | Traumatic arthropathy, lower leg |
| Arthritis | M12.55 | Traumatic arthropathy, pelvic region and thigh |
| Arthritis | M12.54 | Traumatic arthropathy, hand |
| Arthritis | M12.53 | Traumatic arthropathy, forearm |
| Arthritis | M12.52 | Traumatic arthropathy, upper arm |
| Arthritis | M12.51 | Traumatic arthropathy, shoulder region |
| Arthritis | M12.50 | Traumatic arthropathy, multiple sites |
| Arthritis | M12.5 | Traumatic arthropathy |
| Arthritis | M12.49 | Intermittent hydrarthrosis, site unspecified |
| Arthritis | M12.48 | Intermittent hydrarthrosis, other site |
| Arthritis | M12.47 | Intermittent hydrarthrosis, ankle and foot |
| Arthritis | M12.46 | Intermittent hydrarthrosis, lower leg |
| Arthritis | M12.45 | Intermittent hydrarthrosis, pelvic region and thigh |
| Arthritis | M12.44 | Intermittent hydrarthrosis, hand |
| Arthritis | M12.43 | Intermittent hydrarthrosis, forearm |
| Arthritis | M12.42 | Intermittent hydrarthrosis, upper arm |
| Arthritis | M12.41 | Intermittent hydrarthrosis, shoulder region |
| Arthritis | M12.40 | Intermittent hydrarthrosis, multiple sites |
| Arthritis | M12.4 | Intermittent hydrarthrosis |
| Arthritis | M12.39 | Palindromic rheumatism, site unspecified |
| Arthritis | M12.38 | Palindromic rheumatism, other site |
| Arthritis | M12.37 | Palindromic rheumatism, ankle and foot |
| Arthritis | M12.36 | Palindromic rheumatism, lower leg |
| Arthritis | M12.35 | Palindromic rheumatism, pelvic region and thigh |
| Arthritis | M12.34 | Palindromic rheumatism, hand |
| Arthritis | M12.33 | Palindromic rheumatism, forearm |
| Arthritis | M12.32 | Palindromic rheumatism, upper arm |
| Arthritis | M12.31 | Palindromic rheumatism, shoulder region |
| Arthritis | M12.30 | Palindromic rheumatism, multiple sites |
| Arthritis | M12.3 | Palindromic rheumatism |
| Arthritis | M12.29 | Villonodular synovitis (pigmented), site unspecified |
| Arthritis | M12.28 | Villonodular synovitis (pigmented), other site |
| Arthritis | M12.27 | Villonodular synovitis (pigmented), ankle and foot |
| Arthritis | M12.26 | Villonodular synovitis (pigmented), lower leg |
| Arthritis | M12.25 | Villonodular synovitis (pigmented), pelvic region and thigh |
| Arthritis | M12.24 | Villonodular synovitis (pigmented), hand |
| Arthritis | M12.23 | Villonodular synovitis (pigmented), forearm |
| Arthritis | M12.22 | Villonodular synovitis (pigmented), upper arm |
| Arthritis | M12.21 | Villonodular synovitis (pigmented), shoulder region |
| Arthritis | M12.20 | Villonodular synovitis (pigmented), multiple sites |
| Arthritis | M12.2 | Villonodular synovitis (pigmented) |
| Arthritis | M12.19 | Kaschin-Beck disease, site unspecified |
| Arthritis | M12.18 | Kaschin-Beck disease, other site |
| Arthritis | M12.17 | Kaschin-Beck disease, ankle and foot |
| Arthritis | M12.16 | Kaschin-Beck disease, lower leg |
| Arthritis | M12.15 | Kaschin-Beck disease, pelvic region and thigh |
| Arthritis | M12.14 | Kaschin-Beck disease, hand |
| Arthritis | M12.13 | Kaschin-Beck disease, forearm |
| Arthritis | M12.12 | Kaschin-Beck disease, upper arm |
| Arthritis | M12.11 | Kaschin-Beck disease, shoulder region |
| Arthritis | M12.10 | Kaschin-Beck disease, multiple sites |
| Arthritis | M12.1 | Kaschin-Beck disease |
| Arthritis | M12.09 | Chronic postrheumatic arthropathy [Jaccoud], site unspecified |
| Arthritis | M12.08 | Chronic postrheumatic arthropathy [Jaccoud], other site |
| Arthritis | M12.07 | Chronic postrheumatic arthropathy [Jaccoud], ankle and foot |
| Arthritis | M12.06 | Chronic postrheumatic arthropathy [Jaccoud], lower leg |
| Arthritis | M12.05 | Chronic postrheumatic arthropathy [Jaccoud], pelvic region and thigh |
| Arthritis | M12.04 | Chronic postrheumatic arthropathy [Jaccoud], hand |
| Arthritis | M12.03 | Chronic postrheumatic arthropathy [Jaccoud], forearm |
| Arthritis | M12.02 | Chronic postrheumatic arthropathy [Jaccoud], upper arm |
| Arthritis | M12.01 | Chronic postrheumatic arthropathy [Jaccoud], shoulder region |
| Arthritis | M12.00 | Chronic postrheumatic arthropathy [Jaccoud], multiple sites |
| Arthritis | M12.0 | Chronic postrheumatic arthropathy [Jaccoud] |
| Arthritis | M12 | Other specific arthropathies |
| Arthritis | M11.99 | Crystal arthropathy, unspecified, site unspecified |
| Arthritis | M11.98 | Crystal arthropathy, unspecified, other site |
| Arthritis | M11.97 | Crystal arthropathy, unspecified, ankle and foot |
| Arthritis | M11.96 | Crystal arthropathy, unspecified, lower leg |
| Arthritis | M11.95 | Crystal arthropathy, unspecified, pelvic region and thigh |
| Arthritis | M11.94 | Crystal arthropathy, unspecified, hand |
| Arthritis | M11.93 | Crystal arthropathy, unspecified, forearm |
| Arthritis | M11.92 | Crystal arthropathy, unspecified, upper arm |
| Arthritis | M11.91 | Crystal arthropathy, unspecified, shoulder region |
| Arthritis | M11.90 | Crystal arthropathy, unspecified, multiple sites |
| Arthritis | M11.9 | Crystal arthropathy, unspecified |
| Arthritis | M11.89 | Other specified crystal arthropathies, site unspecified |
| Arthritis | M11.88 | Other specified crystal arthropathies, other site |
| Arthritis | M11.87 | Other specified crystal arthropathies, ankle and foot |
| Arthritis | M11.86 | Other specified crystal arthropathies, lower leg |
| Arthritis | M11.85 | Other specified crystal arthropathies, pelvic region and thigh |
| Arthritis | M11.84 | Other specified crystal arthropathies, hand |
| Arthritis | M11.83 | Other specified crystal arthropathies, forearm |
| Arthritis | M11.82 | Other specified crystal arthropathies, upper arm |
| Arthritis | M11.81 | Other specified crystal arthropathies, shoulder region |
| Arthritis | M11.80 | Other specified crystal arthropathies, multiple sites |
| Arthritis | M11.8 | Other specified crystal arthropathies |
| Arthritis | M11.29 | Other chondrocalcinosis, site unspecified |
| Arthritis | M11.28 | Other chondrocalcinosis, other site |
| Arthritis | M11.27 | Other chondrocalcinosis, ankle and foot |
| Arthritis | M11.26 | Other chondrocalcinosis, lower leg |
| Arthritis | M11.25 | Other chondrocalcinosis, pelvic region and thigh |
| Arthritis | M11.24 | Other chondrocalcinosis, hand |
| Arthritis | M11.23 | Other chondrocalcinosis, forearm |
| Arthritis | M11.22 | Other chondrocalcinosis, upper arm |
| Arthritis | M11.21 | Other chondrocalcinosis, shoulder region |
| Arthritis | M11.20 | Other chondrocalcinosis, multiple sites |
| Arthritis | M11.2 | Other chondrocalcinosis |
| Arthritis | M11.19 | Familial chondrocalcinosis, site unspecified |
| Arthritis | M11.18 | Familial chondrocalcinosis, other site |
| Arthritis | M11.17 | Familial chondrocalcinosis, ankle and foot |
| Arthritis | M11.16 | Familial chondrocalcinosis, lower leg |
| Arthritis | M11.15 | Familial chondrocalcinosis, pelvic region and thigh |
| Arthritis | M11.14 | Familial chondrocalcinosis, hand |
| Arthritis | M11.13 | Familial chondrocalcinosis, forearm |
| Arthritis | M11.12 | Familial chondrocalcinosis, upper arm |
| Arthritis | M11.11 | Familial chondrocalcinosis, shoulder region |
| Arthritis | M11.10 | Familial chondrocalcinosis, multiple sites |
| Arthritis | M11.1 | Familial chondrocalcinosis |
| Arthritis | M11.09 | Hydroxyapatite deposition disease, site unspecified |
| Arthritis | M11.08 | Hydroxyapatite deposition disease, other site |
| Arthritis | M11.07 | Hydroxyapatite deposition disease, ankle and foot |
| Arthritis | M11.06 | Hydroxyapatite deposition disease, lower leg |
| Arthritis | M11.05 | Hydroxyapatite deposition disease, pelvic region and thigh |
| Arthritis | M11.04 | Hydroxyapatite deposition disease, hand |
| Arthritis | M11.03 | Hydroxyapatite deposition disease, forearm |
| Arthritis | M11.02 | Hydroxyapatite deposition disease, upper arm |
| Arthritis | M11.01 | Hydroxyapatite deposition disease, shoulder region |
| Arthritis | M11.00 | Hydroxyapatite deposition disease, multiple sites |
| Arthritis | M11.0 | Hydroxyapatite deposition disease |
| Arthritis | M11 | Other crystal arthropathies |
| Arthritis | M07.69 | Other enteropathic arthropathies, site unspecified |
| Arthritis | M07.68 | Other enteropathic arthropathies, other site |
| Arthritis | M07.67 | Other enteropathic arthropathies, ankle and foot |
| Arthritis | M07.66 | Other enteropathic arthropathies, lower leg |
| Arthritis | M07.65 | Other enteropathic arthropathies, pelvic region and thigh |
| Arthritis | M07.64 | Other enteropathic arthropathies, hand |
| Arthritis | M07.63 | Other enteropathic arthropathies, forearm |
| Arthritis | M07.62 | Other enteropathic arthropathies, upper arm |
| Arthritis | M07.61 | Other enteropathic arthropathies, shoulder region |
| Arthritis | M07.60 | Other enteropathic arthropathies, multiple sites |
| Arthritis | M07.6 | Other enteropathic arthropathies |
| Arthritis | M07.59 | Arthropathy in ulcerative colitis (K51.-+), site unspecified |
| Arthritis | M07.58 | Arthropathy in ulcerative colitis (K51.-+), other site |
| Arthritis | M07.57 | Arthropathy in ulcerative colitis (K51.-+), ankle and foot |
| Arthritis | M07.56 | Arthropathy in ulcerative colitis (K51.-+), lower leg |
| Arthritis | M07.55 | Arthropathy in ulcerative colitis (K51.-+), pelvic region and thigh |
| Arthritis | M07.54 | Arthropathy in ulcerative colitis (K51.-+), hand |
| Arthritis | M07.53 | Arthropathy in ulcerative colitis (K51.-+), forearm |
| Arthritis | M07.52 | Arthropathy in ulcerative colitis (K51.-+), upper arm |
| Arthritis | M07.51 | Arthropathy in ulcerative colitis (K51.-+), shoulder region |
| Arthritis | M07.50 | Arthropathy in ulcerative colitis (K51.-+), multiple sites |
| Arthritis | M07.5 | Arthropathy in ulcerative colitis (K51.-+) |
| Arthritis | M07.49 | Arthropathy in Crohn's disease [regional enteritis] (K50.-+), site unspecified |
| Arthritis | M07.48 | Arthropathy in Crohn's disease [regional enteritis] (K50.-+), other site |
| Arthritis | M07.47 | Arthropathy in Crohn's disease [regional enteritis] (K50.-+), ankle and foot |
| Arthritis | M07.46 | Arthropathy in Crohn's disease [regional enteritis] (K50.-+), lower leg |
| Arthritis | M07.45 | Arthropathy in Crohn's disease [regional enteritis] (K50.-+), pelvic region and thigh |
| Arthritis | M07.44 | Arthropathy in Crohn's disease [regional enteritis] (K50.-+), hand |
| Arthritis | M07.43 | Arthropathy in Crohn's disease [regional enteritis] (K50.-+), forearm |
| Arthritis | M07.42 | Arthropathy in Crohn's disease [regional enteritis] (K50.-+), upper arm |
| Arthritis | M07.41 | Arthropathy in Crohn's disease [regional enteritis] (K50.-+), shoulder region |
| Arthritis | M07.40 | Arthropathy in Crohn's disease [regional enteritis] (K50.-+), multiple sites |
| Arthritis | M07.4 | Arthropathy in Crohn's disease [regional enteritis] (K50.-+) |
| Arthritis | M07.39 | Other psoriatic arthropathies (L40.5+), site unspecified |
| Arthritis | M07.38 | Other psoriatic arthropathies (L40.5+), other site |
| Arthritis | M07.37 | Other psoriatic arthropathies (L40.5+), ankle and foot |
| Arthritis | M07.36 | Other psoriatic arthropathies (L40.5+), lower leg |
| Arthritis | M07.35 | Other psoriatic arthropathies (L40.5+), pelvic region and thigh |
| Arthritis | M07.34 | Other psoriatic arthropathies (L40.5+), hand |
| Arthritis | M07.33 | Other psoriatic arthropathies (L40.5+), forearm |
| Arthritis | M07.32 | Other psoriatic arthropathies (L40.5+), upper arm |
| Arthritis | M07.31 | Other psoriatic arthropathies (L40.5+), shoulder region |
| Arthritis | M07.30 | Other psoriatic arthropathies (L40.5+), multiple sites |
| Arthritis | M07.3 | Other psoriatic arthropathies (L40.5+) |
| Arthritis | M07.29 | Psoriatic spondylitis (L40.5+), site unspecified |
| Arthritis | M07.28* | Psoriatic spondylitis (L40.5+), other site |
| Arthritis | M07.27 | Psoriatic spondylitis (L40.5+), ankle and foot |
| Arthritis | M07.26 | Psoriatic spondylitis (L40.5+), lower leg |
| Arthritis | M07.25 | Psoriatic spondylitis (L40.5+), pelvic region and thigh |
| Arthritis | M07.24 | Psoriatic spondylitis (L40.5+), hand |
| Arthritis | M07.23 | Psoriatic spondylitis (L40.5+), forearm |
| Arthritis | M07.22 | Psoriatic spondylitis (L40.5+), upper arm |
| Arthritis | M07.21 | Psoriatic spondylitis (L40.5+), shoulder region |
| Arthritis | M07.20 | Psoriatic spondylitis (L40.5+), multiple sites |
| Arthritis | M07.2 | Psoriatic spondylitis (L40.5+) |
| Arthritis | M07.19 | Arthritis mutilans (L40.5+), site unspecified |
| Arthritis | M07.18 | Arthritis mutilans (L40.5+), other site |
| Arthritis | M07.17 | Arthritis mutilans (L40.5+), ankle and foot |
| Arthritis | M07.16 | Arthritis mutilans (L40.5+), lower leg |
| Arthritis | M07.15 | Arthritis mutilans (L40.5+), pelvic region and thigh |
| Arthritis | M07.14 | Arthritis mutilans (L40.5+), hand |
| Arthritis | M07.13 | Arthritis mutilans (L40.5+), forearm |
| Arthritis | M07.12 | Arthritis mutilans (L40.5+), upper arm |
| Arthritis | M07.11 | Arthritis mutilans (L40.5+), shoulder region |
| Arthritis | M07.10 | Arthritis mutilans (L40.5+), multiple sites |
| Arthritis | M07.1 | Arthritis mutilans (L40.5+) |
| Arthritis | M07.09 | Distal interphalangeal psoriatic arthropathy (L40.5+), site unspecified |
| Arthritis | M07.07 | Distal interphalangeal psoriatic arthropathy (L40.5+), ankle and foot |
| Arthritis | M07.04 | Distal interphalangeal psoriatic arthropathy (L40.5+), hand |
| Arthritis | M07.00 | Distal interphalangeal psoriatic arthropathy (L40.5+), multiple sites |
| Arthritis | M07.0 | Distal interphalangeal psoriatic arthropathy (L40.5+) |
| Arthritis | M07 | Psoriatic and enteropathic arthropathies |
| Arthritis | M06.49 | Inflammatory polyarthropathy, site unspecified |
| Arthritis | M06.48 | Inflammatory polyarthropathy, other site |
| Arthritis | M06.47 | Inflammatory polyarthropathy, ankle and foot |
| Arthritis | M06.46 | Inflammatory polyarthropathy, lower leg |
| Arthritis | M06.45 | Inflammatory polyarthropathy, pelvic region and thigh |
| Arthritis | M06.44 | Inflammatory polyarthropathy, hand |
| Arthritis | M06.43 | Inflammatory polyarthropathy, forearm |
| Arthritis | M06.42 | Inflammatory polyarthropathy, upper arm |
| Arthritis | M06.41 | Inflammatory polyarthropathy, shoulder region |
| Arthritis | M06.40 | Inflammatory polyarthropathy, multiple sites |
| Arthritis | M06.4 | Inflammatory polyarthropathy |
| Asthma | J46 | Status asthmaticus |
| Asthma | J45.9 | Asthma, unspecified |
| Asthma | J45.8 | Mixed Asthma |
| Asthma | J45.1 | Nonallergic asthma |
| Asthma | J45.0 | Predominantly allergic asthma |
| Asthma | J45 | Asthma |
| Bipolar disorder | F31 | Bipolar affective disorder |
| Bipolar disorder | F30 | Manic episode |
| Bladder cancer | D09.0 | Carcinoma in situ, bladder |
| Bladder cancer | C67 | Malignant neoplasm of bladder |
| Breast cancer | D05.9 | Carcinoma in situ of breast, unspecified |
| Breast cancer | D05.7 | Other carcinoma in situ of breast |
| Breast cancer | D05.1 | Intraductal carcinoma in situ of breast |
| Breast cancer | D05.0 | Lobular carcinoma in situ of breast |
| Breast cancer | D05 | Carcinoma in situ of breast |
| Breast cancer | C50 | Malignant neoplasm of breast |
| Cardiac arrhythmia | I49 | Other cardiac arrhythmia |
| Cardiac arrhythmia | I48 | Atrial fibrillation and flutter |
| Cardiac arrhythmia | I47 | Paroxysmal tachycardia |
| Cardiac arrhythmia | I45 | Other conduction disorders |
| Cardiac arrhythmia | I44 | Atrioventricular and left bundle-branch block |
| Cervical cancer | D06.9 | Carcinoma in situ, cervix, unspecified |
| Cervical cancer | D06.7 | Carcinoma in situ, other parts of cervix |
| Cervical cancer | D06.1 | Carcinoma in situ, exocervix |
| Cervical cancer | D06.0 | Carcinoma in situ, endocervix |
| Cervical cancer | D06 | Carcinoma in situ of cervix uteri |
| Cervical cancer | C53 | Malignant neoplasm of cervix uteri |
| Chronic kidney disease | N189 | Chronic kidney disease, unspecified |
| Chronic kidney disease | N185 | Chronic kidney disease, stage 5 |
| Chronic kidney disease | N184 | Chronic kidney disease, stage 4 |
| Chronic kidney disease | N183 | Chronic kidney disease, stage 3 |
| Chronic kidney disease | N11 | Chronic tubulo-interstitial nephritis |
| Chronic kidney disease | N074 | Hereditary nephropathy, not elsewhere classified ; Diffuse endocapillary proliferative glomerulonephritis |
| Chronic kidney disease | N073 | Hereditary nephropathy, not elsewhere classified ; Diffuse mesangial proliferative glomerulonephritis |
| Chronic kidney disease | N072 | Hereditary nephropathy, not elsewhere classified ; Diffuse membranous glomerulonephritis |
| Chronic kidney disease | N03 | Chronic nephritic syndrome |
| Chronic kidney disease | N00 | Acute nephritic syndrome |
| Colon cancer | D01.0 | Carcinoma in situ, colon |
| Colon cancer | C18 | Malignant neoplasm of colon |
| Chronic obstructive pulmonary disease | J449 | Chronic obstructive pulmonary disease, unspecified |
| Chronic obstructive pulmonary disease | J448 | Other specified chronic obstructive pulmonary disease |
| Chronic obstructive pulmonary disease | J441 | Chronic obstructive pulmonary disease with acute exacerbation, unspecified |
| Chronic obstructive pulmonary disease | J440 | Chronic obstructive pulmonary disease with acute lower respiratory infection |
| Chronic obstructive pulmonary disease | J43 | Emphysema |
| Chronic obstructive pulmonary disease | J42 | Unspecified chronic bronchitis |
| Chronic obstructive pulmonary disease | J41 | Simple and mucopurulent chronic bronchitis |
| Connective tissue disease | M36.8 | Systemic disorders of connective tissue in other diseases classified elsewhere |
| Connective tissue disease | M36.4 | Arthropathy in hypersensitivity reactions classified elsewhere |
| Connective tissue disease | M36.3 | Arthropathy in other blood disorders (D50-D76+) |
| Connective tissue disease | M36.2 | Haemophilic arthropathy (D66-D68+) |
| Connective tissue disease | M36.1 | Arthropathy in neoplastic disease (C00-D48+) |
| Connective tissue disease | M36.0 | Dermato(poly)myositis in neoplastic disease (C00-D48+) |
| Connective tissue disease | M36 | Systemic disorders of connective tissue in diseases classified elsewhere |
| Connective tissue disease | M35.9 | Systemic involvement of connective tissue, unspecified |
| Connective tissue disease | M35.8 | Other specified systemic involvement of connective tissue |
| Connective tissue disease | M35.7 | Hypermobility syndrome |
| Connective tissue disease | M35.6 | Relapsing panniculitis [Weber-Christian] |
| Connective tissue disease | M35.5 | Multifocal fibrosclerosis |
| Connective tissue disease | M35.4 | Diffuse (eosinophilic) fasciitis |
| Connective tissue disease | M35.3 | Polymyalgia rheumatica |
| Connective tissue disease | M35.3 | Polymyalgia rheumatica |
| Connective tissue disease | M35.2 | Behcet's disease |
| Connective tissue disease | M35.1 | Other overlap syndromes |
| Connective tissue disease | M35.0 | Sicca syndrome [Sjogren] |
| Connective tissue disease | M35 | Other systemic involvement of connective tissue |
| Connective tissue disease | M34.9 | Systemic sclerosis, unspecified |
| Connective tissue disease | M34.8 | Other forms of systemic sclerosis |
| Connective tissue disease | M34.2 | Systemic sclerosis induced by drugs and chemicals |
| Connective tissue disease | M34.1 | CR(E)ST syndrome |
| Connective tissue disease | M34.0 | Progressive systemic sclerosis |
| Connective tissue disease | M34 | Systemic sclerosis |
| Connective tissue disease | M34 | Systemic sclerosis |
| Connective tissue disease | M33.9 | Dermatopolymyositis, unspecified |
| Connective tissue disease | M33.9 | Dermatopolymyositis, unspecified |
| Connective tissue disease | M33.2 | Polymyositis |
| Connective tissue disease | M33.2 | Polymyositis |
| Connective tissue disease | M33.1 | Other dermatomyositis |
| Connective tissue disease | M33.1 | Other dermatomyositis |
| Connective tissue disease | M33.0 | Juvenile dermatomyositis |
| Connective tissue disease | M33.0 | Juvenile dermatomyositis |
| Connective tissue disease | M33 | Dermatopolymyositis |
| Connective tissue disease | M32.9 | Systemic lupus erythematosus, unspecified |
| Connective tissue disease | M32.8 | Other forms of systemic lupus erythematosus |
| Connective tissue disease | M32.1 | Systemic lupus erythematosus with organ or system involvement |
| Connective tissue disease | M32.0 | Drug-induced systemic lupus erythematosus |
| Connective tissue disease | M32 | Systemic lupus erythematosus |
| Connective tissue disease | M32 | Systemic lupus erythematosus |
| Connective tissue disease | M31.9 | Necrotizing vasculopathy, unspecified |
| Connective tissue disease | M31.8 | Other specified necrotizing vasculopathies |
| Connective tissue disease | M31.7 | Microscopic polyangiitis |
| Connective tissue disease | M31.6 | Other giant cell arteritis |
| Connective tissue disease | M31.5 | Giant cell arteritis with polymyalgia rheumatica |
| Connective tissue disease | M31.4 | Aortic arch syndrome [Takayasu] |
| Connective tissue disease | M31.3 | Wegener's granulomatosis |
| Connective tissue disease | M31.2 | Lethal midline granuloma |
| Connective tissue disease | M31.1 | Thrombotic microangiopathy |
| Connective tissue disease | M31.0 | Hypersensitivity angiitis |
| Connective tissue disease | M31 | Other necrotizing vasculopathies |
| Connective tissue disease | M30.8 | Other conditions related to polyarteritis nodosa |
| Connective tissue disease | M30.3 | Mucocutaneouslymph node syndrome [Kawasaki] |
| Connective tissue disease | M30.2 | Juvenile polyarteritis |
| Connective tissue disease | M30.1 | Polyarteritis with lung involvement [Churg-Strauss] |
| Connective tissue disease | M30.0 | Polyarteritis nodosa |
| Connective tissue disease | M30 | Polyarteritis nodosa and related conditions |
| Connective tissue disease | M06.9 | Rheumatoid arthritis, unspecified |
| Connective tissue disease | M06.3 | Rheumatoid nodule |
| Connective tissue disease | M06.0 | Seronegative rheumatoid arthritis |
| Connective tissue disease | M05 | Seropositive rheumatoid arthritis |
| Dementia | G30 | Alzheimer disease |
| Dementia | F051 | Delirium superimposed on dementia |
| Dementia | F03 | Unspecified dementia |
| Dementia | F02 | Dementia in other diseases classified elsewhere |
| Dementia | F01 | Vascular dementia |
| Dementia | F00 | Dementia in Alzheimer's disease |
| Depression | F381 | Other recurrent mood [affective] disorders |
| Depression | F341 | Dysthymia |
| Depression | F339 | Recurrent depressive disorder, unspecified |
| Depression | F338 | Other recurrent depressive disorders |
| Depression | F334 | Recurrent depressive disorder, currently in remission |
| Depression | F333 | Recurrent depressive disorder, current episode severe with psychotic symptoms |
| Depression | F332 | Recurrent depressive disorder, current episode severe without psychotic symptoms |
| Depression | F331 | Recurrent depressive disorder, current episode moderate |
| Depression | F330 | Recurrent depressive disorder, current episode mild |
| Depression | F33 | Recurrent depressive disorder |
| Depression | F329 | Depressive episode, unspecified |
| Depression | F328 | Other depressive episodes |
| Depression | F323 | Severe depressive episode with psychotic symptoms |
| Depression | F322 | Severe depressive episode without psychotic symptoms |
| Depression | F321 | Moderate depressive episode |
| Depression | F320 | Mild depressive episode |
| Depression | F32 | Depressive episode |
| Diabetes mellitus | O243 | Diabetes mellitus in pregnancy: Pre-existing diabetes mellitus, unspecified |
| Diabetes mellitus | O242 | Diabetes mellitus in pregnancy: Pre-existing malnutrition-related diabetes mellitus |
| Diabetes mellitus | O241 | Diabetes mellitus in pregnancy: Pre-existing diabetes mellitus, non-insulin-dependent |
| Diabetes mellitus | O240 | Diabetes mellitus in pregnancy: Pre-existing diabetes mellitus, insulin-dependent |
| Diabetes mellitus | N083 | Glomerular disorders in diabetes mellitus |
| Diabetes mellitus | M142 | Diabetic arthropathy |
| Diabetes mellitus | H360 | Diabetic retinopathy |
| Diabetes mellitus | H280 | Diabetic cataract |
| Diabetes mellitus | G632 | Diabetic polyneuropathy |
| Diabetes mellitus | G590 | Diabetic mononeuropathy |
| Diabetes mellitus | E14 | Unspecified diabetes mellitus |
| Diabetes mellitus | E13 | Other specified diabetes mellitus |
| Diabetes mellitus | E12 | Malnutrition-related diabetes mellitus |
| Diabetes mellitus | E11 | Non-insulin-dependent diabetes mellitus |
| Diabetes mellitus | E10 | Insulin-dependent diabetes mellitus |
| Eating disorder | F50.9 | Eating disorder, unspecified |
| Eating disorder | F50.8 | Other eating disorders |
| Eating disorder | F50.5 | Vomiting associated with other psychological disturbances |
| Eating disorder | F50.4 | Overeating associated with other psychological disturbances |
| Eating disorder | F50.3 | Atypical bulimia nervosa |
| Eating disorder | F50.2 | Bulimia nervosa |
| Eating disorder | F50.1 | Atypical anorexia nervosa |
| Eating disorder | F50.0 | Anorexia nervosa |
| ENT cancer | D02.1 | Carcinoma in situ, trachea |
| ENT cancer | D02.0 | Carcinoma in situ, larynx |
| ENT cancer | D00.0 | Carcinoma in situ, lip, oral cavity and pharynx |
| ENT cancer | C32 | Malignant neoplasm of larynx |
| ENT cancer | C31 | Malignant neoplasm of accessory sinuses |
| ENT cancer | C30 | Malignant neoplasm of nasal cavity and middle ear |
| ENT cancer | C14 | Malignant neoplasm of other and ill-defined sites in the lip, oral cavity and pharynx |
| ENT cancer | C13 | Malignant neoplasm of hypopharynx |
| ENT cancer | C12 | Malignant neoplasm of piriform sinus |
| ENT cancer | C11 | Malignant neoplasm of nasopharynx |
| ENT cancer | C10 | Malignant neoplasm of oropharynx |
| ENT cancer | C09 | Malignant neoplasm of tonsil |
| ENT cancer | C08 | Malignant neoplasm of other and unspecified major salivary glands |
| ENT cancer | C07 | Malignant neoplasm of parotid gland |
| ENT cancer | C06 | Malignant neoplasm of other and unspecified parts of mouth |
| ENT cancer | C05 | Malignant neoplasm of palate |
| ENT cancer | C04 | Malignant neoplasm of floor of mouth |
| ENT cancer | C03 | Malignant neoplasm of gum |
| ENT cancer | C02 | Malignant neoplasm of other and unspecified parts of tongue |
| ENT cancer | C01 | Malignant neoplasm of base of tongue |
| Epilepsy | G41 | Status epilepticus |
| Epilepsy | G40 | Epilepsy |
| Gout | M140 | Gouty arthropathy due to enzyme defects and other inherited disorders |
| Gout | M10 | Gout |
| Hemiplegia | G82.2 | Paraplegia, unspecified |
| Hemiplegia | G82.1 | Spastic paraplegia |
| Hemiplegia | G82.0 | Flaccid paraplegia |
| Heart failure | I50.9 | Heart failure, unspecified |
| Heart failure | I50.1 | Left ventricular failure |
| Heart failure | I50 | Heart failure |
| Heart failure | I43* | Cardiomyopathy in diseases classified elsewhere |
| Heart failure | I42.0 | Dilated cardiomyopathy (Congestive cardiomyopathy) |
| Heart failure | I42 | Cardiomyopathy |
| Heart failure | I260 | Pulmonary embolism with mention of acute cor pulmonale |
| Heart failure | I132 | Hypertensive heart and renal disease with both (congestive) heart failure and renal failure |
| Heart failure | I130 | Hypertensive heart and renal disease with (congestive) heart failure |
| Heart failure | I110 | Hypertensive heart disease with (congestive) heart failure |
| HIV/AIDS | Z21 | Asymptomatic human immunodeficiency virus [HIV] infection status |
| HIV/AIDS | R75 | Laboratory evidence of human immunodeficiency virus [HIV] |
| HIV/AIDS | B24 | Unspecified human immunodeficiency virus [HIV] disease |
| HIV/AIDS | B23 | Human immunodeficiency virus [HIV] disease resulting in other conditions |
| HIV/AIDS | B227 | HIV disease resulting in multiple diseases classified elsewhere |
| HIV/AIDS | B222 | HIV disease resulting in wasting syndrome |
| HIV/AIDS | B221 | HIV disease resulting in lymphoid interstitial pneumonitis |
| HIV/AIDS | B220 | HIV disease resulting in encephalopathy |
| HIV/AIDS | B219 | HIV disease resulting in unspecified malignant neoplasm |
| HIV/AIDS | B218 | HIV disease resulting in other malignant neoplasms |
| HIV/AIDS | B217 | HIV disease resulting in multiple malignant neoplasms |
| HIV/AIDS | B213 | HIV disease resulting in other malignant neoplasms of lymphoid, haematopoietic and related tissue |
| HIV/AIDS | B212 | HIV disease resulting in other types of non-Hodgkin lymphoma |
| HIV/AIDS | B211 | HIV disease resulting in Burkitt lymphoma |
| HIV/AIDS | B210 | HIV disease resulting in Kaposi sarcoma |
| HIV/AIDS | B209 | HIV disease resulting in unspecified infectious or parasitic disease |
| HIV/AIDS | B208 | HIV disease resulting in other infectious and parasitic diseases |
| HIV/AIDS | B207 | HIV disease resulting in multiple infections |
| HIV/AIDS | B206 | HIV disease resulting in Pneumocystis jirovecii pneumonia |
| HIV/AIDS | B205 | HIV disease resulting in other mycoses |
| HIV/AIDS | B204 | HIV disease resulting in candidiasis |
| HIV/AIDS | B203 | HIV disease resulting in other viral infections |
| HIV/AIDS | B202 | HIV disease resulting in cytomegaloviral disease |
| HIV/AIDS | B201 | HIV disease resulting in other bacterial infections |
| HIV/AIDS | B200 | HIV disease resulting in mycobacterial infection |
| Hyperlipidaemia | E78 | Disorders of lipoprotein metabolism and other lipidaemias |
| Hypertension | I13 | Hypertensive heart and renal disease |
| Hypertension | I12 | Hypertensive renal disease |
| Hypertension | I11 | Hypertensive heart disease |
| Hypertension | I10 | Essential (primary) hypertension |
| Ischaemic heart disease | I25 | Chronic ischaemic heart disease |
| Ischaemic heart disease | I24.1 | Dressler's syndrome |
| Ischaemic heart disease | I24 | Other acute ischaemic heart diseases |
| Ischaemic heart disease | I23 | Certain current complications following acute myocardial infarction |
| Ischaemic heart disease | I22 | Subsequent myocardial infarction |
| Ischaemic heart disease | I21 | Acute myocardial infarction |
| Ischaemic heart disease | I20 | Angina pectoris |
| Learning disability | F89 | Disorders of psychological development |
| Learning disability | F88 | Disorders of psychological development |
| Learning disability | F84.0 | Childhood autism |
| Learning disability | F84 | Disorders of psychological development |
| Learning disability | F83 | Disorders of psychological development |
| Learning disability | F82 | Disorders of psychological development |
| Learning disability | F81 | Disorders of psychological development |
| Learning disability | F80 | Disorders of psychological development |
| Learning disability | F79 | Unspecified mental retardation |
| Learning disability | F78 | Other mental retardation |
| Learning disability | F73 | Profound mental retardation |
| Learning disability | F72 | Severe mental retardation |
| Learning disability | F71 | Moderate mental retardation |
| Learning disability | F70 | Mild mental retardation |
| Leukaemia | D46.9 | Myelodysplastic syndrome, unspecified |
| Leukaemia | D46.7 | Other myelodysplastic syndromes |
| Leukaemia | D46.6 | Myelodysplastic syndrome with isolated del(5q) chromosomal abnormality |
| Leukaemia | D46.5 | Refractory anaemia with multi-lineage dysplasia |
| Leukaemia | D46.4 | Refractory anaemia, unspecified |
| Leukaemia | D46.3 | Refractory anaemia with excess of blasts with transformation |
| Leukaemia | D46.2 | Refractory anaemia with excess of blasts |
| Leukaemia | D46.1 | Refractory anaemia with ring sideroblasts |
| Leukaemia | D46.0 | Refractory anaemia without ring sideroblasts, so stated |
| Leukaemia | D46 | Myelodysplastic syndromes |
| Leukaemia | D45 | Polycythaemia vera |
| Leukaemia | C95 | Leukaemia of unspecified cell type |
| Leukaemia | C947 | Other specified leukaemias |
| Leukaemia | C946 | Myelodysplastic and myeloproliferative disease, not elsewhere classified |
| Leukaemia | C944 | Acute panmyelosis with myelofibrosis |
| Leukaemia | C943 | Mast cell leukaemia |
| Leukaemia | C942 | Acute megakaryoblastic leukaemia |
| Leukaemia | C940 | Acute erythroid leukaemia |
| Leukaemia | C93 | Monocytic leukaemia |
| Leukaemia | C92 | Myeloid leukaemia |
| Leukaemia | C919 | Lymphoid leukaemia, unspecified |
| Leukaemia | C918 | Mature B-cell leukaemia Burkitt-type |
| Leukaemia | C917 | Other lymphoid leukaemia |
| Leukaemia | C916 | Prolymphocytic leukaemia of T-cell type |
| Leukaemia | C913 | Prolymphocytic leukaemia of B-cell type |
| Leukaemia | C911 | Chronic lymphocytic leukaemia of B-cell type |
| Leukaemia | C910 | Acute lymphoblastic leukaemia [ALL] |
| Leukaemia | C903 | Solitary plasmacytoma |
| Leukaemia | C902 | Extramedullary plasmacytoma |
| Leukaemia | C901 | Plasma cell leukaemia |
| Leukaemia | C900 | Multiple myeloma |
| Leukaemia | C884 | Extranodal marginal zone B-cell lymphoma of mucosa-associated lymphoid tissue [MALT-lymphoma] |
| Leukaemia | C882 | Other heavy chain disease |
| Leukaemia | C880 | Waldenstrom macroglobulinaemia |
| Leukaemia | C86 | Other specified types of T/NK-cell lymphoma |
| Liver cancer | D01.5 | Carcinoma in situ, liver, gallbladder and bile ducts |
| Liver cancer | C22 | Malignant neoplasm of liver and intrahepatic bile ducts |
| Liver disease | K77* | Liver disorders in diseases classified elsewhere |
| Liver disease | K76.7 | Hepatorenal syndrome |
| Liver disease | K76.6 | Portal hypertension |
| Liver disease | K76 | Other diseases of liver |
| Liver disease | K75 | Other inflammatory liver diseases |
| Liver disease | K74.6 | Other and unspecified cirrhosis of liver |
| Liver disease | K74.5 | Biliary cirrhosis, unspecified |
| Liver disease | K74.4 | Secondary biliary cirrhosis |
| Liver disease | K74.3 | Primary biliary cirrhosis |
| Liver disease | K74.2 | Hepatic fibrosis with hepatic sclerosis |
| Liver disease | K74.0 | Hepatic fibrosis |
| Liver disease | K74 | Fibrosis and cirrhosis of liver |
| Liver disease | K73 | Chronic hepatitis, not elsewhere classified |
| Liver disease | K72.9 | Hepatic failure, unspecified |
| Liver disease | K72.1 | Chronic hepatic failure |
| Liver disease | K72 | Hepatic failure, not elsewhere classified |
| Liver disease | K71.7 | Toxic liver disease with fibrosis and cirrhosis of liver |
| Liver disease | K71 | Toxic liver disease |
| Liver disease | K70.3 | Alcoholic cirrhosis of liver |
| Liver disease | K70.2 | Alcoholic fibrosis and sclerosis of liver |
| Liver disease | K70 | Alcoholic liver disease |
| Liver disease | I85 | Oesophageal varices |
| Liver cancer | D01.5 | Carcinoma in situ, liver, gallbladder and bile ducts |
| Liver cancer | C22 | Malignant neoplasm of liver and intrahepatic bile ducts |
| Lung cancer | D02.2 | Carcinoma in situ, bronchus and lung |
| Lung cancer | C34 | Malignant neoplasm of bronchus and lung |
| Lymphoma | C96 | Other and unspecified malignant neoplasms of lymphoid, haematopoietic and related tissue |
| Lymphoma | C915 | Adult T-cell lymphoma/leukaemia (HTLV-1-associated) |
| Lymphoma | C914 | Hairy-cell leukaemia |
| Lymphoma | C889 | Malignant immunoproliferative disease, unspecified |
| Lymphoma | C887 | Other malignant immunoproliferative diseases |
| Lymphoma | C883 | Immunoproliferative small intestinal disease |
| Lymphoma | C85 | Other and unspecified types of non-Hodgkin lymphoma |
| Lymphoma | C84 | Mature T/NK-cell lymphomas |
| Lymphoma | C83 | Non-follicular lymphoma |
| Lymphoma | C82 | Follicular lymphoma |
| Lymphoma | C81 | Hodgkin lymphoma |
| Metastatic cancer | C79 | Secondary malignant neoplasm of other and unspecified sites |
| Metastatic cancer | C78 | Secondary malignant neoplasm of respiratory and digestive organs |
| Metastatic cancer | C77 | Secondary and unspecified malignant neoplasm of lymph nodes |
| Obesity | E66 | Obesity |
| Oesophageal cancer | D00.1 | Carcinoma in situ, oesophagus |
| Oesophageal cancer | C15 | Malignant neoplasm of oesophagus |
| Other cancer | D48.9 | Neoplasm of uncertain or unknown behaviour, unspecified |
| Other cancer | D48.7 | Neoplasm of uncertain or unknown behaviour, other specified sites |
| Other cancer | D48.6 | Neoplasm of uncertain or unknown behaviour, breast |
| Other cancer | D48.5 | Neoplasm of uncertain or unknown behaviour, skin |
| Other cancer | D48.4 | Neoplasm of uncertain or unknown behaviour, peritoneum |
| Other cancer | D48.3 | Neoplasm of uncertain or unknown behaviour, retroperitoneum |
| Other cancer | D48.2 | Neoplasm of uncertain or unknown behaviour, peripheral nerves and autonomic nervous system |
| Other cancer | D48.1 | Neoplasm of uncertain or unknown behaviour, connective and other soft tissue |
| Other cancer | D48.0 | Neoplasm of uncertain or unknown behaviour, bone and articular cartilage |
| Other cancer | D48 | Neoplasm of uncertain or unknown behaviour of other and unspecified sites |
| Other cancer | D47.9 | Neoplasm of uncertain or unknown behaviour of lymphoid, haematopoietic and related tissue, unspecified |
| Other cancer | D47.7 | Other specified neoplasms of uncertain or unknown behaviour of lymphoid, haematopoietic and related tissue |
| Other cancer | D47.5 | Chronic eosinophilic leukaemia [hypereosinophilic syndrome] |
| Other cancer | D47.4 | Osteomyelofibrosis |
| Other cancer | D47.3 | Essential (haemorrhagic) thrombocythaemia |
| Other cancer | D47.2 | Monoclonal gammopathy of undetermined significance (MGUS) |
| Other cancer | D47.1 | Chronic myeloproliferative disease |
| Other cancer | D47.0 | Histiocytic and mast cell tumours of uncertain and unknown behaviour |
| Other cancer | D47 | Other neoplasms of uncertain or unknown behaviour of lymphoid, haematopoietic and related tissue |
| Other cancer | D44.9 | Neoplasm of uncertain or unknown behaviour, endocrine gland, unspecified |
| Other cancer | D44.8 | Neoplasm of uncertain or unknown behaviour, pluriglandular involvement |
| Other cancer | D44.7 | Neoplasm of uncertain or unknown behaviour, aortic body and other paraganglia |
| Other cancer | D44.6 | Neoplasm of uncertain or unknown behaviour, carotid body |
| Other cancer | D44.5 | Neoplasm of uncertain or unknown behaviour, pineal gland |
| Other cancer | D44.4 | Neoplasm of uncertain or unknown behaviour, craniopharyngeal duct |
| Other cancer | D44.3 | Neoplasm of uncertain or unknown behaviour, pituitary gland |
| Other cancer | D44.2 | Neoplasm of uncertain or unknown behaviour, parathyroid gland |
| Other cancer | D44.1 | Neoplasm of uncertain or unknown behaviour, adrenal gland |
| Other cancer | D44.0 | Neoplasm of uncertain or unknown behaviour, thyroid gland |
| Other cancer | D44 | Neoplasm of uncertain or unknown behaviour of endocrine glands |
| Other cancer | D43.9 | Neoplasm of uncertain or unknown behaviour, central nervous system, unspecified |
| Other cancer | D43.7 | Neoplasm of uncertain or unknown behaviour, other parts of central nervous system |
| Other cancer | D43.4 | Neoplasm of uncertain or unknown behaviour, spinal cord |
| Other cancer | D43.3 | Neoplasm of uncertain or unknown behaviour, cranial nerves |
| Other cancer | D43.2 | Neoplasm of uncertain or unknown behaviour, brain, unspecified |
| Other cancer | D43.1 | Neoplasm of uncertain or unknown behaviour, brain, infratentorial |
| Other cancer | D43.0 | Neoplasm of uncertain or unknown behaviour, brain, supratentorial |
| Other cancer | D43 | Neoplasm of uncertain or unknown behaviour of brain and central nervous system |
| Other cancer | D42.9 | Neoplasm of uncertain or unknown behaviour, meninges, unspecified |
| Other cancer | D42.1 | Neoplasm of uncertain or unknown behaviour, spinal meninges |
| Other cancer | D42.0 | Neoplasm of uncertain or unknown behaviour, cerebral meninges |
| Other cancer | D42 | Neoplasm of uncertain or unknown behaviour of meninges |
| Other cancer | D41.9 | Neoplasm of uncertain or unknown behaviour, urinary organ, unspecified |
| Other cancer | D41.7 | Neoplasm of uncertain or unknown behaviour, other urinary organs |
| Other cancer | D41.4 | Neoplasm of uncertain or unknown behaviour, bladder |
| Other cancer | D41.3 | Neoplasm of uncertain or unknown behaviour, urethra |
| Other cancer | D41.2 | Neoplasm of uncertain or unknown behaviour, ureter |
| Other cancer | D41.1 | Neoplasm of uncertain or unknown behaviour, renal pelvis |
| Other cancer | D41.0 | Neoplasm of uncertain or unknown behaviour, kidney |
| Other cancer | D41 | Neoplasm of uncertain or unknown behaviour of urinary organs |
| Other cancer | D40.9 | Neoplasm of uncertain or unknown behaviour, male genital organ, unspecified |
| Other cancer | D40.7 | Neoplasm of uncertain or unknown behaviour, other male genital organs |
| Other cancer | D40.1 | Neoplasm of uncertain or unknown behaviour, testis |
| Other cancer | D40.0 | Neoplasm of uncertain or unknown behaviour, prostate |
| Other cancer | D40 | Neoplasm of uncertain or unknown behaviour of male genital organs |
| Other cancer | D39.9 | Neoplasm of uncertain or unknown behaviour, female genital organ, unspecified |
| Other cancer | D39.7 | Neoplasm of uncertain or unknown behaviour, other female genital organs |
| Other cancer | D39.2 | Neoplasm of uncertain or unknown behaviour, placenta |
| Other cancer | D39.1 | Neoplasm of uncertain or unknown behaviour, ovary |
| Other cancer | D39.0 | Neoplasm of uncertain or unknown behaviour, uterus |
| Other cancer | D39 | Neoplasm of uncertain or unknown behaviour of female genital organs |
| Other cancer | D38.6 | Neoplasm of uncertain or unknown behaviour, respiratory organ, unspecified |
| Other cancer | D38.5 | Neoplasm of uncertain or unknown behaviour, other respiratory organs |
| Other cancer | D38.4 | Neoplasm of uncertain or unknown behaviour, thymus |
| Other cancer | D38.3 | Neoplasm of uncertain or unknown behaviour, mediastinum |
| Other cancer | D38.2 | Neoplasm of uncertain or unknown behaviour, pleura |
| Other cancer | D38.1 | Neoplasm of uncertain or unknown behaviour, trachea, bronchus and lung |
| Other cancer | D38.0 | Neoplasm of uncertain or unknown behaviour, larynx |
| Other cancer | D38 | Neoplasm of uncertain or unknown behaviour of middle ear and respiratory and intrathoracic organs |
| Other cancer | D37.9 | Neoplasm of uncertain or unknown behaviour, digestive organ, unspecified |
| Other cancer | D37.7 | Neoplasm of uncertain or unknown behaviour, other digestive organs |
| Other cancer | D37.6 | Neoplasm of uncertain or unknown behaviour, liver, gallbladder and bile ducts |
| Other cancer | D37.5 | Neoplasm of uncertain or unknown behaviour, rectum |
| Other cancer | D37.4 | Neoplasm of uncertain or unknown behaviour, colon |
| Other cancer | D37.3 | Neoplasm of uncertain or unknown behaviour, appendix |
| Other cancer | D37.2 | Neoplasm of uncertain or unknown behaviour, small intestine |
| Other cancer | D37.1 | Neoplasm of uncertain or unknown behaviour, stomach |
| Other cancer | D37.0 | Neoplasm of uncertain or unknown behaviour, lip, oral cavity and pharynx |
| Other cancer | D37 | Neoplasm of uncertain or unknown behaviour of oral cavity and digestive organs |
| Other cancer | D18.1 | Lymphangioma, any site |
| Other cancer | D18.0 | Haemangioma, any site |
| Other cancer | D18 | Haemangioma and lymphangioma, any site |
| Other cancer | D09.7 | Carcinoma in situ of other specified sites |
| Other cancer | D09.3 | Carcinoma in situ, thyroid and other endocrine glands |
| Other cancer | D09.2 | Carcinoma in situ, eye |
| Other cancer | D09 | Carcinoma in situ of other and unspecified sites |
| Other cancer | D07 | Carcinoma in situ of other and unspecified genital organs |
| Other cancer | C97 | Malignant neoplasms of independent (primary) multiple sites |
| Other cancer | C80 | Malignant neoplasm without specification of site |
| Other cancer | C76 | Malignant neoplasm of other and ill-defined sites |
| Other cancer | C75 | Malignant neoplasm of other endocrine glands and related structures |
| Other cancer | C74 | Malignant neoplasm of adrenal gland |
| Other cancer | C73 | Malignant neoplasm of thyroid gland |
| Other cancer | C72 | Malignant neoplasm of spinal cord, cranial nerves and other parts of central nervous system |
| Other cancer | C71 | Malignant neoplasm of brain |
| Other cancer | C70 | Malignant neoplasm of meninges |
| Other cancer | C69 | Malignant neoplasm of eye and adnexa |
| Other cancer | C49 | Malignant neoplasm of other connective and soft tissue |
| Other cancer | C48 | Malignant neoplasm of retroperitoneum and peritoneum |
| Other cancer | C47 | Malignant neoplasm of peripheral nerves and autonomic nervous system |
| Other cancer | C46 | Kaposi sarcoma |
| Other cancer | C45 | Mesothelioma |
| Other cancer | C43 | Malignant melanoma of skin |
| Other cancer | C41 | Malignant neoplasm of bone and articular cartilage of other and unspecified sites |
| Other cancer | C40 | Malignant neoplasm of bone and articular cartilage of limbs |
| Other cancer | C39 | Malignant neoplasm of other and ill-defined sites in the respiratory system and intrathoracic organs |
| Other cancer | C38 | Malignant neoplasm of heart, mediastinum and pleura |
| Other cancer | C37 | Malignant neoplasm of thymus |
| Other cancer | C00 | Malignant neoplasm of lip |
| Other female reproductive cancer | D25.9 | Leiomyoma of uterus, unspecified |
| Other female reproductive cancer | D25.2 | Subserosal leiomyoma of uterus |
| Other female reproductive cancer | D25.1 | Intramural leiomyoma of uterus |
| Other female reproductive cancer | D25.0 | Submucous leiomyoma of uterus |
| Other female reproductive cancer | D25 | Leiomyoma of uterus |
| Other female reproductive cancer | D07.3 | Carcinoma in situ, other and unspecified female genital organs |
| Other female reproductive cancer | D07.2 | Carcinoma in situ, vagina |
| Other female reproductive cancer | D07.1 | Carcinoma in situ, vulva |
| Other female reproductive cancer | D07.0 | Carcinoma in situ, endometrium |
| Other female reproductive cancer | C58 | Malignant neoplasm of placenta |
| Other female reproductive cancer | C57 | Malignant neoplasm of other and unspecified female genital organs |
| Other female reproductive cancer | C55 | Malignant neoplasm of uterus, part unspecified |
| Other female reproductive cancer | C54 | Malignant neoplasm of corpus uteri |
| Other female reproductive cancer | C52 | Malignant neoplasm of vagina |
| Other female reproductive cancer | C51 | Malignant neoplasm of vulva |
| Other gastrointestinal cancer | D01.9 | Carcinoma in situ, digestive organ, unspecified |
| Other gastrointestinal cancer | D01.7 | Carcinoma in situ, other specified digestive organs |
| Other gastrointestinal cancer | D01.4 | Carcinoma in situ, other and unspecified parts of intestine |
| Other gastrointestinal cancer | D01.3 | Carcinoma in situ, anus and anal canal |
| Other gastrointestinal cancer | C26 | Malignant neoplasm of other and ill-defined digestive organs |
| Other gastrointestinal cancer | C24 | Malignant neoplasm of other and unspecified parts of biliary tract |
| Other gastrointestinal cancer | C23 | Malignant neoplasm of gallbladder |
| Other gastrointestinal cancer | C21 | Malignant neoplasm of anus and anal canal |
| Other gastrointestinal cancer | C17 | Malignant neoplasm of small intestine |
| Other male reproductive cancer | D07.6 | Carcinoma in situ, other and unspecified male genital organs |
| Other male reproductive cancer | D07.4 | Carcinoma in situ, penis |
| Other male reproductive cancer | C63 | Malignant neoplasm of other and unspecified male genital organs |
| Other male reproductive cancer | C62 | Malignant neoplasm of testis |
| Other male reproductive cancer | C60 | Malignant neoplasm of penis |
| Other respiratory cancer | D02.4 | Carcinoma in situ, respiratory system, unspecified |
| Other respiratory cancer | D02.3 | Carcinoma in situ, other parts of respiratory system |
| Other respiratory cancer | D02 | Carcinoma in situ of middle ear and respiratory system |
| Other respiratory cancer | C33 | Malignant neoplasm of trachea |
| Other urological cancer | D09.1 | Carcinoma in situ, other and unspecified urinary organs |
| Other urological cancer | C68 | Malignant neoplasm of other and unspecified urinary organs |
| Other urological cancer | C66 | Malignant neoplasm of ureter |
| Osteoporosis | M82* | Osteoporosis in diseases classified elsewhere |
| Osteoporosis | M81 | Osteoporosis without pathological fracture |
| Osteoporosis | M80 | Osteoporosis with pathological fracture |
| Ovarian cancer | C56 | Malignant neoplasm of ovary |
| Pancreatic cancer | C25 | Malignant neoplasm of pancreas |
| Peptic ulcer disease | K28 | Gastrojejunal ulcer |
| Peptic ulcer disease | K27 | Peptic ulcer, site unspecified |
| Peptic ulcer disease | K26 | Duodenal ulcer |
| Peptic ulcer disease | K25 | Gastric ulcer |
| Peripheral arterial disease | I79 | Disorders of arteries, arterioles and capillaries in diseases classified elsewhere |
| Peripheral arterial disease | I78 | Diseases of capillaries |
| Peripheral arterial disease | I77 | Other disorders of arteries and arterioles |
| Peripheral arterial disease | I745 | Embolism and thrombosis of iliac artery |
| Peripheral arterial disease | I744 | Embolism and thrombosis of arteries of extremities, unspecified |
| Peripheral arterial disease | I743 | Embolism and thrombosis of arteries of lower extremities |
| Peripheral arterial disease | I74 | Arterial embolism and thrombosis |
| Peripheral arterial disease | I739 | Peripheral vascular disease, unspecified |
| Peripheral arterial disease | I738 | Other specified peripheral vascular diseases |
| Peripheral arterial disease | I731 | Thromboangiitis obliterans [Buerger] |
| Peripheral arterial disease | I730 | Raynaud's syndrome |
| Peripheral arterial disease | I73 | Other peripheral vascular diseases |
| Peripheral arterial disease | I72 | Other aneurysm and dissection |
| Peripheral arterial disease | I72 | Other aneurysm and dissection |
| Peripheral arterial disease | I72 | Other aneurysm and dissection |
| Peripheral arterial disease | I719 | Aortic aneurysm of unspecified site, without mention of rupture |
| Peripheral arterial disease | I719 | Aortic aneurysm of unspecified site, without mention of rupture |
| Peripheral arterial disease | I718 | Aortic aneurysm of unspecified site, ruptured |
| Peripheral arterial disease | I718 | Aortic aneurysm of unspecified site, ruptured |
| Peripheral arterial disease | I716 | Thoracoabdominal aortic aneurysm, without mention of rupture |
| Peripheral arterial disease | I716 | Thoracoabdominal aortic aneurysm, without mention of rupture |
| Peripheral arterial disease | I715 | Thoracoabdominal aortic aneurysm, ruptured |
| Peripheral arterial disease | I715 | Thoracoabdominal aortic aneurysm, ruptured |
| Peripheral arterial disease | I714 | Abdominal aortic aneurysm, without mention of rupture |
| Peripheral arterial disease | I714 | Abdominal aortic aneurysm, without mention of rupture |
| Peripheral arterial disease | I713 | Abdominal aortic aneurysm, ruptured |
| Peripheral arterial disease | I713 | Abdominal aortic aneurysm, ruptured |
| Peripheral arterial disease | I712 | Thoracic aortic aneurysm, without mention of rupture |
| Peripheral arterial disease | I712 | Thoracic aortic aneurysm, without mention of rupture |
| Peripheral arterial disease | I711 | Thoracic aortic aneurysm, ruptured |
| Peripheral arterial disease | I711 | Thoracic aortic aneurysm, ruptured |
| Peripheral arterial disease | I710 | Dissection of aorta [any part] |
| Peripheral arterial disease | I710 | Dissection of aorta [any part] |
| Peripheral arterial disease | I71 | Aortic aneurysm and dissection |
| Peripheral arterial disease | I70 | Atherosclerosis |
| Peripheral arterial disease | I70 | Atherosclerosis |
| Prostate cancer | D07.5 | Carcinoma in situ, prostate |
| Prostate cancer | C61 | Malignant neoplasm of prostate |
| Psychoses | F30.2 | psychotic types of manic episode |
| Psychoses | F29 | Unspecified nonorganic psychosis |
| Psychoses | F28 | Other nonorganic psychotic disorders |
| Psychoses | F23 | Acute and transient psychotic disorders |
| Psychoses | F22 | Persistent delusional disorders |
| Psychoses | F09 | Unspecified organic or symptomatic mental disorder |
| Rectal cancer | D01.2 | Carcinoma in situ, rectum |
| Rectal cancer | D01.1 | Carcinoma in situ, rectosigmoid junction |
| Rectal cancer | C20 | Malignant neoplasm of rectum |
| Rectal cancer | C19 | Malignant neoplasm of rectosigmoid junction |
| Renal cancer | C65 | Malignant neoplasm of renal pelvis |
| Renal cancer | C64 | Malignant neoplasm of kidney, except renal pelvis |
| Rheumatoid arthritis | M08.4 | Pauciarticular juvenile arthritis |
| Rheumatoid arthritis | M08.3 | Juvenile polyarthritis (seronegative) |
| Rheumatoid arthritis | M08.2 | Juvenile arthritis with systemic onset |
| Rheumatoid arthritis | M08.0 | Juvenile rheumatoid arthritis |
| Rheumatoid arthritis | M06.9 | Rheumatoid arthritis, unspecified |
| Rheumatoid arthritis | M06.8 | Other specified rheumatoid arthritis |
| Rheumatoid arthritis | M06.3 | Rheumatoid nodule |
| Rheumatoid arthritis | M06.2 | Rheumatoid bursitis |
| Rheumatoid arthritis | M06.1 | Adult-onset Still's disease |
| Rheumatoid arthritis | M06.0 | Seronegative rheumatoid arthritis |
| Rheumatoid arthritis | M05.9 | Seropositive rheumatoid arthritis, unspecified |
| Rheumatoid arthritis | M05.8 | Other seropositive rheumatoid arthritis |
| Rheumatoid arthritis | M05.3 | Rheumatoid arthritis with involvement of oth organs and sys |
| Rheumatoid arthritis | M05.2 | Rheumatoid vasculitis |
| Rheumatoid arthritis | M05.1 | Rheumatoid lung disease |
| Rheumatoid arthritis | M05.0 | Felty's syndrome |
| Rheumatoid arthritis | J99.0 | Rheumatoid lung disease |
| Rheumatoid arthritis | I52.8 | Rheumatoid carditis |
| Schizophrenia | F29 | Unspecified nonorganic psychosis |
| Schizophrenia | F28 | Other nonorganic psychotic disorders |
| Schizophrenia | F25 | Schizoaffective disorders |
| Schizophrenia | F24 | Induced delusional disorder |
| Schizophrenia | F23 | Acute and transient psychotic disorders |
| Schizophrenia | F22 | Persistent delusional disorders |
| Schizophrenia | F21 | Schizotypal disorder |
| Schizophrenia | F20 | Schizophrenia |
| Skin cancer | D04.9 | Carcinoma in situ, skin, unspecified |
| Skin cancer | D04.8 | Carcinoma in situ, skin of other sites |
| Skin cancer | D04.7 | Carcinoma in situ, skin of lower limb, including hip |
| Skin cancer | D04.6 | Carcinoma in situ, skin of upper limb, including shoulder |
| Skin cancer | D04.5 | Carcinoma in situ, skin of trunk |
| Skin cancer | D04.4 | Carcinoma in situ, skin of scalp and neck |
| Skin cancer | D04.3 | Carcinoma in situ, skin of other and unspecified parts of face |
| Skin cancer | D04.2 | Carcinoma in situ, skin of ear and external auricular canal |
| Skin cancer | D04.1 | Carcinoma in situ, skin of eyelid, including canthus |
| Skin cancer | D04.0 | Carcinoma in situ, skin of lip |
| Skin cancer | D04 | Carcinoma in situ of skin |
| Skin cancer | D03.9 | Melanoma in situ, unspecified |
| Skin cancer | D03.8 | Melanoma in situ of other sites |
| Skin cancer | D03.7 | Melanoma in situ of lower limb, including hip |
| Skin cancer | D03.6 | Melanoma in situ of upper limb, including shoulder |
| Skin cancer | D03.5 | Melanoma in situ of trunk |
| Skin cancer | D03.4 | Melanoma in situ of scalp and neck |
| Skin cancer | D03.3 | Melanoma in situ of other and unspecified parts of face |
| Skin cancer | D03.2 | Melanoma in situ of ear and external auricular canal |
| Skin cancer | D03.1 | Melanoma in situ of eyelid, including canthus |
| Skin cancer | D03.0 | Melanoma in situ of lip |
| Skin cancer | D03 | Melanoma in situ |
| Skin cancer | C44 | Other malignant neoplasms of skin |
| Stomach cancer | D00.2 | Carcinoma in situ, stomach |
| Stomach cancer | C16 | Malignant neoplasm of stomach |
| Stroke/TIA | I698 | Sequelae of other and unspecified cerebrovascular diseases |
| Stroke/TIA | I694 | Sequelae of stroke, not specified as haemorrhage or infarction |
| Stroke/TIA | I693 | Sequelae of cerebral infarction |
| Stroke/TIA | I692 | Sequelae of other nontraumatic intracranial haemorrhage |
| Stroke/TIA | I691 | Sequelae of intracerebral haemorrhage |
| Stroke/TIA | I690 | Sequelae of subarachnoid haemorrhage |
| Stroke/TIA | I69 | Sequelae of cerebrovascular disease |
| Stroke/TIA | I68 | Cerebrovascular disorders in diseases classified elsewhere |
| Stroke/TIA | I679 | Cerebrovascular disease, unspecified |
| Stroke/TIA | I672 | Cerebral atherosclerosis |
| Stroke/TIA | I67 | Other cerebrovascular diseases |
| Stroke/TIA | I66 | Occlusion and stenosis of cerebral arteries, not resulting in cerebral infarction |
| Stroke/TIA | I65 | Occlusion and stenosis of precerebral arteries, not resulting in cerebral infarction |
| Stroke/TIA | I64 | Stroke, not specified as haemorrhage or infarction |
| Stroke/TIA | I63 | Cerebral infarction |
| Stroke/TIA | I63 | Cerebral infarction |
| Stroke/TIA | I629 | Intracranial haemorrhage (nontraumatic), unspecified |
| Stroke/TIA | I621 | Nontraumatic extradural haemorrhage |
| Stroke/TIA | I620 | Subdural haemorrhage (acute)(nontraumatic) |
| Stroke/TIA | I62 | Other nontraumatic intracranial haemorrhage |
| Stroke/TIA | I61 | Intracerebral haemorrhage |
| Stroke/TIA | I61 | Intracerebral haemorrhage |
| Stroke/TIA | I61 | Intracerebral haemorrhage |
| Stroke/TIA | I60 | Subarachnoid haemorrhage |
| Stroke/TIA | I60 | Subarachnoid haemorrhage |
| Stroke/TIA | I60 | Subarachnoid haemorrhage |
| Stroke/TIA | G467 | Other lacunar syndromes |
| Stroke/TIA | G466 | Pure sensory lacunar syndrome |
| Stroke/TIA | G465 | Pure motor lacunar syndrome |
| Stroke/TIA | G464 | Cerebellar stroke syndrome |
| Stroke/TIA | G463 | Brain stem stroke syndrome |
| Substance abuse | F19 | Mental and behavioural disorders due to multiple drug use and use of other psychoactive substances |
| Substance abuse | F18 | Mental and behavioural disorders due to use of volatile solvents |
| Substance abuse | F16 | Mental and behavioural disorders due to use of hallucinogens |
| Substance abuse | F15 | Mental and behavioural disorders due to use of other stimulants, including caffeine |
| Substance abuse | F14 | Mental and behavioural disorders due to use of cocaine |
| Substance abuse | F13 | Mental and behavioural disorders due to use of sedatives or hypnotics |
| Substance abuse | F12 | Mental and behavioural disorders due to use of cannabinoids |
| Substance abuse | F11 | Mental and behavioural disorders due to use of opioids |
| Substance abuse | F10 | Mental and behavioural disorders due to use of alcohol |
| Unspecified cancer | D09.9 | Carcinoma in situ, unspecified |
| Unspecified cancer | D01 | Carcinoma in situ of other and unspecified digestive organs |
