# Supplementary material for: Patterns and temporal trends of comorbidity among adult patients with incident cardiovascular disease in the UK between 2000 and 2014: A population-based cohort study
Source: PLoS Med. 2018 Mar 6;15(3):e1002513. doi: 10.1371/journal.pmed.1002513 (PMC5839540; doi:10.1371/journal.pmed.1002513)
Supplement: S2 Table — (DOCX) [file pmed.1002513.s008.docx]

| **Year** | **Women** | **Men** |
| --- | --- | --- |
| 2000 | 73.6 | 67.7 |
| 2001 | 73.6 | 67.6 |
| 2002 | 73.6 | 67.5 |
| 2003 | 73.8 | 67.5 |
| 2004 | 73.8 | 67.4 |
| 2005 | 73.9 | 67.6 |
| 2006 | 73.7 | 67.3 |
| 2007 | 73.8 | 67.2 |
| 2008 | 74.0 | 67.6 |
| 2009 | 74.3 | 67.6 |
| 2010 | 73.9 | 67.6 |
| 2011 | 73.9 | 67.6 |
| 2012 | 74.1 | 67.8 |
| 2013 | 73.7 | 67.8 |
| 2014 | 73.9 | 67.6 |
