# Supplementary material for: Patterns and temporal trends of comorbidity among adult patients with incident cardiovascular disease in the UK between 2000 and 2014: A population-based cohort study
Source: PLoS Med. 2018 Mar 6;15(3):e1002513. doi: 10.1371/journal.pmed.1002513 (PMC5839540; doi:10.1371/journal.pmed.1002513)
Supplement: S3 Table — (DOCX) [file pmed.1002513.s009.docx]

1. Overall during 2000 to 2014

| **Condition count** | **Cases** | **Persons** | **Crude** | | | **Age-sex-standardised** | | |
| --- | --- | --- | --- | --- | --- | --- | --- | --- |
|  |  |  | Rate | Lower 95% CI | Upper 95% CI | Rate | Lower 95% CI | Upper 95% CI |
| 0 | 20678 | 229205 | 9.0% | 8.8% | 9.2% | 18.9% | 17.1% | 21.2% |
| 1 | 36819 | 229205 | 16.1% | 15.9% | 16.3% | 20.8% | 19.5% | 22.5% |
| 2 | 42859 | 229205 | 18.7% | 18.5% | 18.9% | 19.7% | 18.6% | 21.3% |
| 3 | 39209 | 229205 | 17.1% | 16.9% | 17.3% | 15.7% | 14.4% | 17.5% |
| 4 | 31620 | 229205 | 13.8% | 13.6% | 14.0% | 10.0% | 9.3% | 11.1% |
| ≥5 | 58020 | 229205 | 25.3% | 25.1% | 25.5% | 15.0% | 14.3% | 16.1% |

1. Annual for each year 2000 to 2014

| **Condition count** | **Year** | **Cases** | **Persons** | **Crude** | | | **Age-sex-standardised** | | |
| --- | --- | --- | --- | --- | --- | --- | --- | --- | --- |
|  |  |  |  | Rate | Lower 95% CI | Upper 95% CI | Rate | Lower 95% CI | Upper 95% CI |
| 0 | 2000 | 2193 | 14504 | 15.1% | 14.3% | 16.0% | 32.1% | 23.0% | 48.2% |
| 1 | 2000 | 3515 | 14504 | 24.2% | 23.4% | 25.1% | 24.1% | 21.3% | 34.9% |
| 2 | 2000 | 3418 | 14504 | 23.6% | 22.7% | 24.4% | 18.6% | 16.9% | 29.0% |
| 3 | 2000 | 2388 | 14504 | 16.5% | 15.6% | 17.3% | 11.8% | 10.2% | 22.4% |
| 4 | 2000 | 1457 | 14504 | 10.0% | 9.2% | 10.9% | 7.1% | 6.0% | 17.8% |
| ≥5 | 2000 | 1533 | 14504 | 10.6% | 9.7% | 11.4% | 6.3% | 5.6% | 17.0% |
| 0 | 2001 | 2326 | 15905 | 14.6% | 13.8% | 15.4% | 24.1% | 19.2% | 33.5% |
| 1 | 2001 | 3628 | 15905 | 22.8% | 22.0% | 23.6% | 24.2% | 21.5% | 31.7% |
| 2 | 2001 | 3585 | 15905 | 22.5% | 21.8% | 23.3% | 22.3% | 17.6% | 31.5% |
| 3 | 2001 | 2690 | 15905 | 16.9% | 16.1% | 17.7% | 13.4% | 11.6% | 20.7% |
| 4 | 2001 | 1779 | 15905 | 11.2% | 10.4% | 12.0% | 6.9% | 6.2% | 13.9% |
| ≥5 | 2001 | 1897 | 15905 | 11.9% | 11.1% | 12.7% | 9.0% | 5.2% | 18.1% |
| 0 | 2002 | 2188 | 16883 | 13.0% | 12.2% | 13.7% | 22.7% | 18.4% | 30.2% |
| 1 | 2002 | 3706 | 16883 | 22.0% | 21.2% | 22.7% | 26.9% | 21.9% | 35.0% |
| 2 | 2002 | 3823 | 16883 | 22.6% | 21.9% | 23.4% | 19.1% | 17.3% | 24.6% |
| 3 | 2002 | 3011 | 16883 | 17.8% | 17.1% | 18.6% | 15.2% | 11.9% | 22.0% |
| 4 | 2002 | 1959 | 16883 | 11.6% | 10.8% | 12.4% | 8.4% | 7.1% | 13.8% |
| ≥5 | 2002 | 2196 | 16883 | 13.0% | 12.2% | 13.8% | 7.6% | 6.8% | 12.9% |
| 0 | 2003 | 1857 | 16356 | 11.4% | 10.6% | 12.1% | 25.8% | 17.0% | 41.7% |
| 1 | 2003 | 3376 | 16356 | 20.6% | 19.9% | 21.4% | 24.9% | 21.6% | 36.0% |
| 2 | 2003 | 3675 | 16356 | 22.5% | 21.7% | 23.2% | 19.4% | 17.1% | 30.0% |
| 3 | 2003 | 2958 | 16356 | 18.1% | 17.3% | 18.9% | 13.5% | 11.9% | 24.0% |
| 4 | 2003 | 2037 | 16356 | 12.5% | 11.7% | 13.2% | 7.8% | 6.7% | 18.4% |
| ≥5 | 2003 | 2453 | 16356 | 15.0% | 14.2% | 15.8% | 8.6% | 7.9% | 19.1% |
| 0 | 2004 | 1700 | 16059 | 10.6% | 9.8% | 11.4% | 17.0% | 14.6% | 38.3% |
| 1 | 2004 | 3111 | 16059 | 19.4% | 18.6% | 20.2% | 22.1% | 19.8% | 43.1% |
| 2 | 2004 | 3471 | 16059 | 21.6% | 20.8% | 22.4% | 20.8% | 18.4% | 41.9% |
| 3 | 2004 | 2921 | 16059 | 18.2% | 17.4% | 19.0% | 14.8% | 13.1% | 36.1% |
| 4 | 2004 | 2070 | 16059 | 12.9% | 12.1% | 13.7% | 8.5% | 7.6% | 30.4% |
| ≥5 | 2004 | 2785 | 16059 | 17.3% | 16.6% | 18.1% | 10.2% | 9.2% | 31.9% |
| 0 | 2005 | 1522 | 15682 | 9.7% | 8.9% | 10.5% | 19.6% | 13.3% | 33.3% |
| 1 | 2005 | 2721 | 15682 | 17.4% | 16.6% | 18.1% | 21.6% | 18.3% | 32.7% |
| 2 | 2005 | 3289 | 15682 | 21.0% | 20.2% | 21.8% | 23.1% | 16.7% | 36.6% |
| 3 | 2005 | 2848 | 15682 | 18.2% | 17.4% | 18.9% | 13.4% | 11.8% | 23.9% |
| 4 | 2005 | 2196 | 15682 | 14.0% | 13.2% | 14.8% | 10.2% | 8.4% | 20.9% |
| ≥5 | 2005 | 3106 | 15682 | 19.8% | 19.0% | 20.6% | 12.1% | 10.6% | 22.7% |
| 0 | 2006 | 1368 | 15672 | 8.7% | 7.9% | 9.5% | 15.9% | 13.6% | 37.2% |
| 1 | 2006 | 2532 | 15672 | 16.2% | 15.4% | 17.0% | 26.6% | 15.2% | 53.8% |
| 2 | 2006 | 3033 | 15672 | 19.4% | 18.6% | 20.1% | 18.8% | 16.7% | 39.9% |
| 3 | 2006 | 2845 | 15672 | 18.2% | 17.4% | 18.9% | 15.4% | 13.6% | 36.7% |
| 4 | 2006 | 2251 | 15672 | 14.4% | 13.6% | 15.2% | 9.0% | 8.4% | 30.8% |
| ≥5 | 2006 | 3643 | 15672 | 23.2% | 22.5% | 24.0% | 14.3% | 12.8% | 35.7% |
| 0 | 2007 | 1261 | 15412 | 8.2% | 7.4% | 9.0% | 18.0% | 14.9% | 39.5% |
| 1 | 2007 | 2217 | 15412 | 14.4% | 13.6% | 15.2% | 16.9% | 14.9% | 38.1% |
| 2 | 2007 | 2791 | 15412 | 18.1% | 17.3% | 18.9% | 18.8% | 16.3% | 40.0% |
| 3 | 2007 | 2772 | 15412 | 18.0% | 17.2% | 18.8% | 15.3% | 13.5% | 36.6% |
| 4 | 2007 | 2311 | 15412 | 15.0% | 14.2% | 15.8% | 10.1% | 9.0% | 31.8% |
| ≥5 | 2007 | 4059 | 15412 | 26.3% | 25.5% | 27.1% | 14.4% | 13.4% | 35.7% |
| 0 | 2008 | 1138 | 15828 | 7.2% | 6.4% | 8.0% | 13.9% | 11.9% | 35.4% |
| 1 | 2008 | 2166 | 15828 | 13.7% | 12.9% | 14.5% | 18.6% | 16.3% | 39.7% |
| 2 | 2008 | 2715 | 15828 | 17.2% | 16.4% | 18.0% | 19.1% | 16.9% | 40.2% |
| 3 | 2008 | 2796 | 15828 | 17.7% | 16.9% | 18.5% | 14.8% | 13.1% | 36.1% |
| 4 | 2008 | 2361 | 15828 | 14.9% | 14.1% | 15.7% | 10.3% | 9.1% | 31.9% |
| ≥5 | 2008 | 4651 | 15828 | 29.4% | 28.6% | 30.2% | 16.8% | 15.4% | 37.9% |
| 0 | 2009 | 1066 | 16007 | 6.7% | 5.9% | 7.5% | 19.2% | 12.8% | 33.0% |
| 1 | 2009 | 2064 | 16007 | 12.9% | 12.1% | 13.7% | 17.9% | 15.6% | 28.6% |
| 2 | 2009 | 2579 | 16007 | 16.1% | 15.3% | 16.9% | 16.9% | 15.0% | 27.4% |
| 3 | 2009 | 2687 | 16007 | 16.8% | 16.0% | 17.6% | 17.8% | 11.7% | 31.3% |
| 4 | 2009 | 2462 | 16007 | 15.4% | 14.6% | 16.2% | 10.8% | 9.6% | 21.4% |
| ≥5 | 2009 | 5149 | 16007 | 32.2% | 31.4% | 33.0% | 17.4% | 16.3% | 27.6% |
| 0 | 2010 | 998 | 15807 | 6.3% | 5.5% | 7.1% | 12.1% | 10.1% | 33.8% |
| 1 | 2010 | 1881 | 15807 | 11.9% | 11.1% | 12.7% | 16.5% | 14.4% | 37.8% |
| 2 | 2010 | 2468 | 15807 | 15.6% | 14.8% | 16.4% | 18.5% | 16.1% | 39.7% |
| 3 | 2010 | 2726 | 15807 | 17.2% | 16.4% | 18.0% | 15.0% | 13.2% | 36.3% |
| 4 | 2010 | 2360 | 15807 | 14.9% | 14.1% | 15.7% | 11.1% | 9.7% | 32.8% |
| ≥5 | 2010 | 5373 | 15807 | 34.0% | 33.2% | 34.8% | 20.2% | 18.4% | 41.2% |
| 0 | 2011 | 899 | 15052 | 6.0% | 5.2% | 6.8% | 14.9% | 10.4% | 24.2% |
| 1 | 2011 | 1748 | 15052 | 11.6% | 10.8% | 12.4% | 17.3% | 14.7% | 24.9% |
| 2 | 2011 | 2300 | 15052 | 15.3% | 14.5% | 16.1% | 20.8% | 16.1% | 30.1% |
| 3 | 2011 | 2400 | 15052 | 15.9% | 15.1% | 16.8% | 16.4% | 12.0% | 25.4% |
| 4 | 2011 | 2273 | 15052 | 15.1% | 14.3% | 15.9% | 10.4% | 9.3% | 17.3% |
| ≥5 | 2011 | 5432 | 15052 | 36.1% | 35.3% | 36.9% | 20.2% | 18.9% | 27.1% |
| 0 | 2012 | 816 | 14821 | 5.5% | 4.7% | 6.3% | 11.7% | 9.3% | 33.5% |
| 1 | 2012 | 1654 | 14821 | 11.2% | 10.3% | 12.0% | 15.5% | 13.3% | 36.9% |
| 2 | 2012 | 2173 | 14821 | 14.7% | 13.8% | 15.5% | 17.3% | 14.7% | 38.6% |
| 3 | 2012 | 2297 | 14821 | 15.5% | 14.7% | 16.3% | 14.4% | 12.5% | 35.8% |
| 4 | 2012 | 2223 | 14821 | 15.0% | 14.2% | 15.8% | 12.3% | 10.3% | 33.9% |
| ≥5 | 2012 | 5657 | 14821 | 38.2% | 37.3% | 39.0% | 22.2% | 20.6% | 43.1% |
| 0 | 2013 | 722 | 13507 | 5.3% | 4.5% | 6.2% | 10.4% | 8.3% | 21.2% |
| 1 | 2013 | 1383 | 13507 | 10.2% | 9.4% | 11.1% | 19.6% | 13.3% | 33.4% |
| 2 | 2013 | 1918 | 13507 | 14.2% | 13.3% | 15.1% | 19.3% | 16.5% | 30.1% |
| 3 | 2013 | 2088 | 13507 | 15.5% | 14.6% | 16.3% | 13.6% | 11.8% | 24.2% |
| 4 | 2013 | 2097 | 13507 | 15.5% | 14.7% | 16.4% | 15.4% | 9.5% | 29.0% |
| ≥5 | 2013 | 5299 | 13507 | 39.2% | 38.4% | 40.1% | 21.7% | 20.4% | 31.9% |
| 0 | 2014 | 624 | 11715 | 5.3% | 4.4% | 6.3% | 13.6% | 10.6% | 24.7% |
| 1 | 2014 | 1117 | 11715 | 9.5% | 8.6% | 10.5% | 13.2% | 11.1% | 24.0% |
| 2 | 2014 | 1621 | 11715 | 13.8% | 12.9% | 14.8% | 15.6% | 13.3% | 26.4% |
| 3 | 2014 | 1782 | 11715 | 15.2% | 14.3% | 16.1% | 22.0% | 13.7% | 37.9% |
| 4 | 2014 | 1784 | 11715 | 15.2% | 14.3% | 16.2% | 11.2% | 9.7% | 21.8% |
| ≥5 | 2014 | 4787 | 11715 | 40.9% | 39.9% | 41.8% | 24.3% | 22.1% | 34.8% |
