# Supplementary material for: Patterns and temporal trends of comorbidity among adult patients with incident cardiovascular disease in the UK between 2000 and 2014: A population-based cohort study
Source: PLoS Med. 2018 Mar 6;15(3):e1002513. doi: 10.1371/journal.pmed.1002513 (PMC5839540; doi:10.1371/journal.pmed.1002513)
Supplement: S4 Table — (DOCX) [file pmed.1002513.s010.docx]

1. Overall during 2000 to 2014

| **Condition** | **Cases** | **Persons** | **Crude** | | | **Age-sex-standardised** | | |
| --- | --- | --- | --- | --- | --- | --- | --- | --- |
|  |  |  | Rate | Lower 95% CI | Upper 95% CI | Rate | Lower 95% CI | Upper 95% CI |
| Adjustment disorder | 5077 | 229205 | 2.2% | 2.2% | 2.3% | 4.4% | 2.8% | 7.5% |
| Affective disorder | 826 | 229205 | 0.4% | 0.3% | 0.4% | 0.4% | 0.3% | 2.7% |
| Anxiety | 30255 | 229205 | 13.2% | 13.1% | 13.3% | 15.0% | 13.7% | 17.6% |
| Arthritis | 85527 | 229205 | 37.3% | 37.1% | 37.5% | 20.9% | 19.5% | 23.5% |
| Asthma | 31103 | 229205 | 13.6% | 13.4% | 13.7% | 17.7% | 15.8% | 20.8% |
| Bipolar disorder | 1236 | 229205 | 0.5% | 0.5% | 0.6% | 1.1% | 0.4% | 3.9% |
| Bladder cancer | 2410 | 229205 | 1.1% | 1.0% | 1.1% | 0.4% | 0.4% | 2.8% |
| Breast cancer | 4981 | 110803 | 4.5% | 4.4% | 4.6% | 2.2% | 2.1% | 5.2% |
| Cardiac arrhythmia | 27695 | 229205 | 12.1% | 12.0% | 12.2% | 7.1% | 6.0% | 9.6% |
| Cervical cancer | 401 | 110803 | 0.4% | 0.3% | 0.4% | 0.6% | 0.5% | 3.8% |
| Chronic kidney disease | 24314 | 229205 | 10.6% | 10.5% | 10.7% | 5.3% | 4.9% | 7.4% |
| Colon cancer | 2472 | 229205 | 1.1% | 1.0% | 1.1% | 0.9% | 0.3% | 3.6% |
| Chronic obstructive pulmonary disease | 20363 | 229205 | 8.9% | 8.8% | 9.0% | 4.5% | 4.3% | 6.6% |
| Connective tissue disease | 10440 | 229205 | 4.6% | 4.5% | 4.6% | 2.4% | 2.2% | 4.5% |
| Dementia | 8595 | 229205 | 3.7% | 3.7% | 3.8% | 1.0% | 0.9% | 3.2% |
| Depression | 42973 | 229205 | 18.7% | 18.6% | 18.9% | 23.0% | 21.3% | 26.0% |
| Diabetes mellitus | 33307 | 229205 | 14.5% | 14.4% | 14.7% | 11.2% | 10.1% | 13.7% |
| Eating disorder | 423 | 229205 | 0.2% | 0.2% | 0.2% | 0.7% | 0.5% | 3.0% |
| Ent cancer | 899 | 229205 | 0.4% | 0.4% | 0.4% | 0.2% | 0.2% | 2.6% |
| Epilepsy | 5057 | 229205 | 2.2% | 2.1% | 2.3% | 3.6% | 3.0% | 6.0% |
| Gout | 14255 | 229205 | 6.2% | 6.1% | 6.3% | 3.4% | 3.2% | 5.6% |
| Hemiplegia | 777 | 229205 | 0.3% | 0.3% | 0.4% | 1.2% | 0.3% | 4.2% |
| Heart failure | 19090 | 229205 | 8.3% | 8.2% | 8.4% | 4.1% | 3.8% | 6.3% |
| Hiv/aids | 3975 | 229205 | 1.7% | 1.7% | 1.8% | 1.2% | 1.0% | 3.4% |
| Hyperlipidaemia | 35304 | 229205 | 15.4% | 15.3% | 15.6% | 11.3% | 10.7% | 13.6% |
| Hypertension | 109365 | 229205 | 47.7% | 47.5% | 47.9% | 28.9% | 27.7% | 31.4% |
| Learning disability | 967 | 229205 | 0.4% | 0.4% | 0.4% | 1.6% | 0.9% | 4.4% |
| Leukaemia | 2048 | 229205 | 0.9% | 0.9% | 0.9% | 0.6% | 0.5% | 2.9% |
| Liver disease | 3086 | 229205 | 1.3% | 1.3% | 1.4% | 1.5% | 1.3% | 3.7% |
| Liver cancer | 92 | 229205 | 0.0% | 0.0% | 0.0% | 0.0% | 0.0% | 2.5% |
| Lung cancer | 1107 | 229205 | 0.5% | 0.5% | 0.5% | 0.3% | 0.2% | 2.6% |
| Lymphoma | 1532 | 229205 | 0.7% | 0.6% | 0.7% | 0.7% | 0.4% | 3.2% |
| Metastatic cancer | 3200 | 229205 | 1.4% | 1.3% | 1.4% | 0.8% | 0.7% | 3.1% |
| Obesity | 22315 | 229205 | 9.7% | 9.6% | 9.9% | 10.8% | 10.2% | 12.9% |
| Oesophageal cancer | 453 | 229205 | 0.2% | 0.2% | 0.2% | 0.1% | 0.1% | 2.5% |
| Other cancer | 5711 | 229205 | 2.5% | 2.4% | 2.6% | 1.8% | 1.5% | 4.0% |
| Other female reproductive cancer | 2346 | 110803 | 2.1% | 2.0% | 2.2% | 3.2% | 1.6% | 7.1% |
| Other gastrointestinal cancer | 467 | 229205 | 0.2% | 0.2% | 0.2% | 0.1% | 0.1% | 2.5% |
| Other male reproductive cancer | 287 | 118402 | 0.2% | 0.2% | 0.3% | 0.3% | 0.2% | 1.7% |
| Other respiratory cancer | 813 | 229205 | 0.4% | 0.3% | 0.4% | 0.2% | 0.2% | 2.6% |
| Other urological cancer | 359 | 229205 | 0.2% | 0.1% | 0.2% | 0.1% | 0.1% | 2.5% |
| Osteoporosis | 15655 | 229205 | 6.8% | 6.7% | 6.9% | 2.9% | 2.5% | 5.2% |
| Ovarian cancer | 445 | 110803 | 0.4% | 0.4% | 0.4% | 0.2% | 0.2% | 3.6% |
| Peripheral arterial disease | 16522 | 229205 | 7.2% | 7.1% | 7.3% | 3.8% | 3.6% | 6.0% |
| Pancreatic cancer | 172 | 229205 | 0.1% | 0.1% | 0.1% | 0.0% | 0.0% | 2.5% |
| Prostate cancer | 4258 | 118402 | 3.6% | 3.5% | 3.7% | 1.4% | 1.3% | 2.7% |
| Psychoses | 1843 | 229205 | 0.8% | 0.8% | 0.8% | 1.3% | 0.6% | 4.0% |
| Peptic ulcer disease | 13249 | 229205 | 5.8% | 5.7% | 5.9% | 3.4% | 3.2% | 5.5% |
| Rectal cancer | 1493 | 229205 | 0.7% | 0.6% | 0.7% | 0.3% | 0.2% | 2.6% |
| Renal cancer | 539 | 229205 | 0.2% | 0.2% | 0.3% | 0.1% | 0.1% | 2.5% |
| Rheumatoid arthritis | 5768 | 229205 | 2.5% | 2.5% | 2.6% | 1.5% | 1.4% | 3.7% |
| Schizophrenia | 1892 | 229205 | 0.8% | 0.8% | 0.9% | 1.0% | 0.8% | 3.2% |
| Skin cancer | 8112 | 229205 | 3.5% | 3.5% | 3.6% | 2.1% | 1.3% | 4.7% |
| Stomach cancer | 379 | 229205 | 0.2% | 0.1% | 0.2% | 0.1% | 0.1% | 2.5% |
| Substance abuse | 6768 | 229205 | 3.0% | 2.9% | 3.0% | 5.1% | 4.6% | 7.3% |
| Unspecified cancer | 3648 | 229205 | 1.6% | 1.5% | 1.6% | 1.0% | 0.6% | 3.4% |

1. Annual for each year from 2000 to 2014

| **Condition** | **Year** | **Cases** | **Persons** | **Crude** | | | **Age-sex-standardised** | | |
| --- | --- | --- | --- | --- | --- | --- | --- | --- | --- |
|  |  |  |  | Rate | Lower 95% CI | Upper 95% CI | Rate | Lower 95% CI | Upper 95% CI |
| Adjustment disorder | 2000 | 50 | 3234 | 1.6% | 1.2% | 2.0% | 2.5% | 1.4% | 16.2% |
| Adjustment disorder | 2001 | 169 | 9424.5 | 1.8% | 1.5% | 2.1% | 4.2% | 1.5% | 17.1% |
| Adjustment disorder | 2002 | 286.5 | 15243 | 1.9% | 1.7% | 2.1% | 6.1% | 1.5% | 24.3% |
| Adjustment disorder | 2003 | 396 | 20512 | 1.9% | 1.8% | 2.1% | 3.9% | 2.3% | 8.1% |
| Adjustment disorder | 2004 | 488 | 25003 | 2.0% | 1.8% | 2.1% | 3.0% | 2.4% | 14.8% |
| Adjustment disorder | 2005 | 606.5 | 29065 | 2.1% | 1.9% | 2.3% | 3.3% | 2.7% | 4.1% |
| Adjustment disorder | 2006 | 721 | 32942.5 | 2.2% | 2.0% | 2.4% | 3.4% | 2.8% | 4.3% |
| Adjustment disorder | 2007 | 822 | 36548.5 | 2.3% | 2.1% | 2.4% | 3.3% | 2.8% | 4.1% |
| Adjustment disorder | 2008 | 945 | 39402.5 | 2.4% | 2.3% | 2.6% | 3.4% | 2.9% | 4.2% |
| Adjustment disorder | 2009 | 1012 | 42274.5 | 2.4% | 2.3% | 2.5% | 3.3% | 2.8% | 3.9% |
| Adjustment disorder | 2010 | 1104 | 43957.5 | 2.6% | 2.4% | 2.7% | 3.3% | 2.8% | 9.3% |
| Adjustment disorder | 2011 | 1172.5 | 45143.5 | 2.7% | 2.5% | 2.7% | 3.5% | 2.7% | 26.4% |
| Adjustment disorder | 2012 | 1245.5 | 45639.5 | 2.8% | 2.6% | 2.9% | 3.5% | 3.1% | 15.5% |
| Adjustment disorder | 2013 | 1232 | 43644.5 | 2.9% | 2.7% | 3.0% | 6.7% | 2.1% | 21.4% |
| Adjustment disorder | 2014 | 1098 | 38363.5 | 2.9% | 2.7% | 3.0% | 3.8% | 3.0% | 8.0% |
| Affective disorder | 2000 | 5.5 | 3234 | 0.2% | 0.1% | 0.4% | 0.1% | 0.0% | 13.9% |
| Affective disorder | 2001 | 18 | 9424.5 | 0.2% | 0.1% | 0.3% | 0.2% | 0.1% | 12.2% |
| Affective disorder | 2002 | 31.5 | 15243 | 0.2% | 0.1% | 0.3% | 0.2% | 0.1% | 16.2% |
| Affective disorder | 2003 | 54 | 20512 | 0.3% | 0.2% | 0.3% | 0.4% | 0.2% | 3.8% |
| Affective disorder | 2004 | 72 | 25003 | 0.3% | 0.2% | 0.4% | 0.3% | 0.2% | 12.6% |
| Affective disorder | 2005 | 80.5 | 29065 | 0.3% | 0.2% | 0.3% | 0.3% | 0.2% | 0.8% |
| Affective disorder | 2006 | 95.5 | 32942.5 | 0.3% | 0.2% | 0.4% | 0.4% | 0.2% | 0.9% |
| Affective disorder | 2007 | 104.5 | 36548.5 | 0.3% | 0.2% | 0.3% | 0.3% | 0.2% | 0.8% |
| Affective disorder | 2008 | 120.5 | 39402.5 | 0.3% | 0.3% | 0.4% | 0.3% | 0.2% | 0.7% |
| Affective disorder | 2009 | 137 | 42274.5 | 0.3% | 0.3% | 0.4% | 0.4% | 0.3% | 0.9% |
| Affective disorder | 2010 | 145 | 43957.5 | 0.3% | 0.3% | 0.4% | 0.3% | 0.2% | 6.6% |
| Affective disorder | 2011 | 158.5 | 45143.5 | 0.4% | 0.3% | 0.4% | 0.4% | 0.2% | 24.3% |
| Affective disorder | 2012 | 170 | 45639.5 | 0.4% | 0.3% | 0.4% | 0.4% | 0.3% | 12.6% |
| Affective disorder | 2013 | 164 | 43644.5 | 0.4% | 0.3% | 0.4% | 0.3% | 0.3% | 12.5% |
| Affective disorder | 2014 | 146 | 38363.5 | 0.4% | 0.3% | 0.4% | 0.4% | 0.3% | 4.6% |
| Anxiety | 2000 | 294 | 3234 | 9.1% | 8.1% | 10.1% | 11.0% | 8.6% | 24.5% |
| Anxiety | 2001 | 921.5 | 9424.5 | 9.9% | 9.2% | 10.4% | 10.1% | 8.3% | 20.9% |
| Anxiety | 2002 | 1581 | 15243 | 10.5% | 9.9% | 10.9% | 10.4% | 8.9% | 24.7% |
| Anxiety | 2003 | 2212 | 20512 | 10.9% | 10.4% | 11.2% | 11.0% | 10.1% | 14.3% |
| Anxiety | 2004 | 2767.5 | 25003 | 11.2% | 10.7% | 11.5% | 11.9% | 10.8% | 23.0% |
| Anxiety | 2005 | 3335 | 29065 | 11.7% | 11.1% | 11.8% | 12.9% | 12.0% | 14.1% |
| Anxiety | 2006 | 3902 | 32942.5 | 12.1% | 11.5% | 12.2% | 13.4% | 12.4% | 14.5% |
| Anxiety | 2007 | 4481.5 | 36548.5 | 12.5% | 11.9% | 12.6% | 13.8% | 12.9% | 15.0% |
| Anxiety | 2008 | 5007.5 | 39402.5 | 13.0% | 12.4% | 13.0% | 14.4% | 13.4% | 15.5% |
| Anxiety | 2009 | 5519 | 42274.5 | 13.4% | 12.7% | 13.4% | 14.7% | 13.8% | 15.8% |
| Anxiety | 2010 | 5904.5 | 43957.5 | 13.8% | 13.1% | 13.8% | 14.7% | 13.6% | 20.6% |
| Anxiety | 2011 | 6159 | 45143.5 | 14.1% | 13.3% | 14.0% | 14.6% | 13.1% | 35.9% |
| Anxiety | 2012 | 6407.5 | 45639.5 | 14.5% | 13.7% | 14.4% | 15.2% | 14.1% | 26.7% |
| Anxiety | 2013 | 6295.5 | 43644.5 | 14.9% | 14.1% | 14.8% | 16.0% | 14.5% | 27.0% |
| Anxiety | 2014 | 5649.5 | 38363.5 | 15.3% | 14.4% | 15.1% | 17.4% | 15.8% | 21.8% |
| Arthritis | 2000 | 959 | 3234 | 29.8% | 28.1% | 31.3% | 19.5% | 17.5% | 32.2% |
| Arthritis | 2001 | 2833.5 | 9424.5 | 30.2% | 29.1% | 31.0% | 18.0% | 14.3% | 29.9% |
| Arthritis | 2002 | 4731 | 15243 | 31.2% | 30.3% | 31.8% | 19.8% | 13.7% | 36.8% |
| Arthritis | 2003 | 6494 | 20512 | 31.9% | 31.0% | 32.3% | 20.1% | 19.2% | 23.4% |
| Arthritis | 2004 | 8069 | 25003 | 32.6% | 31.7% | 32.9% | 19.8% | 18.9% | 30.7% |
| Arthritis | 2005 | 9532 | 29065 | 33.2% | 32.3% | 33.3% | 23.3% | 22.5% | 24.4% |
| Arthritis | 2006 | 10997 | 32942.5 | 33.8% | 32.9% | 33.9% | 23.8% | 23.0% | 24.8% |
| Arthritis | 2007 | 12394 | 36548.5 | 34.5% | 33.4% | 34.4% | 24.1% | 23.3% | 25.0% |
| Arthritis | 2008 | 13557 | 39402.5 | 35.0% | 33.9% | 34.9% | 24.4% | 23.6% | 25.4% |
| Arthritis | 2009 | 14807.5 | 42274.5 | 35.7% | 34.6% | 35.5% | 24.6% | 23.9% | 25.5% |
| Arthritis | 2010 | 15575.5 | 43957.5 | 36.1% | 35.0% | 35.9% | 22.6% | 21.7% | 28.3% |
| Arthritis | 2011 | 16180 | 45143.5 | 36.6% | 35.4% | 36.3% | 19.5% | 18.3% | 40.4% |
| Arthritis | 2012 | 16508 | 45639.5 | 37.0% | 35.7% | 36.6% | 22.9% | 21.9% | 34.0% |
| Arthritis | 2013 | 16031 | 43644.5 | 37.6% | 36.3% | 37.2% | 22.4% | 21.5% | 33.3% |
| Arthritis | 2014 | 14279.5 | 38363.5 | 38.2% | 36.7% | 37.7% | 24.6% | 22.0% | 29.6% |
| Asthma | 2000 | 368 | 3234 | 11.4% | 10.3% | 12.5% | 12.0% | 9.6% | 25.6% |
| Asthma | 2001 | 1059 | 9424.5 | 11.3% | 10.6% | 11.9% | 15.0% | 10.3% | 27.6% |
| Asthma | 2002 | 1733 | 15243 | 11.4% | 10.9% | 11.9% | 18.4% | 10.6% | 36.9% |
| Asthma | 2003 | 2375.5 | 20512 | 11.7% | 11.2% | 12.0% | 15.5% | 13.3% | 19.6% |
| Asthma | 2004 | 2968 | 25003 | 12.0% | 11.5% | 12.3% | 14.9% | 13.1% | 26.4% |
| Asthma | 2005 | 3540 | 29065 | 12.3% | 11.8% | 12.6% | 14.6% | 13.4% | 16.1% |
| Asthma | 2006 | 4126.5 | 32942.5 | 12.7% | 12.2% | 12.9% | 14.7% | 13.6% | 16.1% |
| Asthma | 2007 | 4712.5 | 36548.5 | 13.1% | 12.6% | 13.2% | 15.3% | 14.2% | 16.6% |
| Asthma | 2008 | 5172.5 | 39402.5 | 13.3% | 12.8% | 13.5% | 16.2% | 15.0% | 17.6% |
| Asthma | 2009 | 5708.5 | 42274.5 | 13.7% | 13.2% | 13.8% | 16.5% | 15.3% | 17.8% |
| Asthma | 2010 | 6025.5 | 43957.5 | 13.9% | 13.4% | 14.0% | 16.6% | 15.1% | 22.6% |
| Asthma | 2011 | 6289.5 | 45143.5 | 14.2% | 13.6% | 14.3% | 17.8% | 15.6% | 39.0% |
| Asthma | 2012 | 6464.5 | 45639.5 | 14.4% | 13.8% | 14.5% | 21.7% | 15.5% | 36.3% |
| Asthma | 2013 | 6300.5 | 43644.5 | 14.7% | 14.1% | 14.8% | 19.0% | 17.0% | 30.2% |
| Asthma | 2014 | 5647.5 | 38363.5 | 15.1% | 14.4% | 15.1% | 21.5% | 17.6% | 27.8% |
| Bipolar disorder | 2000 | 11.5 | 3234 | 0.4% | 0.2% | 0.6% | 0.7% | 0.2% | 14.5% |
| Bipolar disorder | 2001 | 32 | 9424.5 | 0.3% | 0.2% | 0.5% | 0.4% | 0.2% | 12.3% |
| Bipolar disorder | 2002 | 51.5 | 15243 | 0.3% | 0.3% | 0.4% | 0.4% | 0.2% | 16.3% |
| Bipolar disorder | 2003 | 73.5 | 20512 | 0.4% | 0.3% | 0.4% | 0.5% | 0.3% | 3.9% |
| Bipolar disorder | 2004 | 83.5 | 25003 | 0.3% | 0.3% | 0.4% | 0.5% | 0.3% | 12.6% |
| Bipolar disorder | 2005 | 102.5 | 29065 | 0.4% | 0.3% | 0.4% | 0.4% | 0.3% | 0.9% |
| Bipolar disorder | 2006 | 116.5 | 32942.5 | 0.4% | 0.3% | 0.4% | 0.4% | 0.3% | 0.9% |
| Bipolar disorder | 2007 | 135 | 36548.5 | 0.4% | 0.3% | 0.4% | 0.4% | 0.3% | 0.9% |
| Bipolar disorder | 2008 | 155.5 | 39402.5 | 0.4% | 0.3% | 0.5% | 0.4% | 0.3% | 0.9% |
| Bipolar disorder | 2009 | 169 | 42274.5 | 0.4% | 0.3% | 0.5% | 0.6% | 0.4% | 1.0% |
| Bipolar disorder | 2010 | 186.5 | 43957.5 | 0.4% | 0.4% | 0.5% | 0.7% | 0.4% | 6.9% |
| Bipolar disorder | 2011 | 196 | 45143.5 | 0.4% | 0.4% | 0.5% | 0.7% | 0.3% | 24.6% |
| Bipolar disorder | 2012 | 213.5 | 45639.5 | 0.5% | 0.4% | 0.5% | 0.6% | 0.4% | 12.9% |
| Bipolar disorder | 2013 | 203 | 43644.5 | 0.5% | 0.4% | 0.5% | 3.8% | 0.3% | 18.9% |
| Bipolar disorder | 2014 | 186.5 | 38363.5 | 0.5% | 0.4% | 0.6% | 0.6% | 0.4% | 4.9% |
| Bladder cancer | 2000 | 23.5 | 3234 | 0.7% | 0.5% | 1.1% | 0.5% | 0.2% | 14.1% |
| Bladder cancer | 2001 | 76.5 | 9424.5 | 0.8% | 0.6% | 1.0% | 0.3% | 0.2% | 12.2% |
| Bladder cancer | 2002 | 127 | 15243 | 0.8% | 0.7% | 1.0% | 0.3% | 0.3% | 16.3% |
| Bladder cancer | 2003 | 156.5 | 20512 | 0.7% | 0.7% | 0.9% | 0.3% | 0.3% | 3.7% |
| Bladder cancer | 2004 | 182.5 | 25003 | 0.7% | 0.6% | 0.8% | 0.4% | 0.4% | 12.7% |
| Bladder cancer | 2005 | 214.5 | 29065 | 0.7% | 0.6% | 0.8% | 0.5% | 0.4% | 1.0% |
| Bladder cancer | 2006 | 240.5 | 32942.5 | 0.7% | 0.6% | 0.8% | 0.5% | 0.4% | 0.9% |
| Bladder cancer | 2007 | 272.5 | 36548.5 | 0.7% | 0.7% | 0.8% | 0.5% | 0.4% | 0.9% |
| Bladder cancer | 2008 | 294 | 39402.5 | 0.7% | 0.7% | 0.8% | 0.5% | 0.4% | 0.9% |
| Bladder cancer | 2009 | 304.5 | 42274.5 | 0.7% | 0.6% | 0.8% | 0.4% | 0.4% | 0.8% |
| Bladder cancer | 2010 | 326.5 | 43957.5 | 0.7% | 0.7% | 0.8% | 0.3% | 0.3% | 6.5% |
| Bladder cancer | 2011 | 337.5 | 45143.5 | 0.7% | 0.7% | 0.8% | 0.3% | 0.2% | 24.3% |
| Bladder cancer | 2012 | 340 | 45639.5 | 0.7% | 0.7% | 0.8% | 0.3% | 0.3% | 12.5% |
| Bladder cancer | 2013 | 344 | 43644.5 | 0.7% | 0.7% | 0.9% | 0.5% | 0.4% | 12.7% |
| Bladder cancer | 2014 | 311 | 38363.5 | 0.8% | 0.7% | 0.9% | 0.3% | 0.3% | 4.5% |
| Breast cancer | 2000 | 94 | 6468 | 1.5% | 1.2% | 1.8% | 0.6% | 0.5% | 24.5% |
| Breast cancer | 2001 | 263 | 18849 | 1.4% | 1.2% | 1.6% | 0.6% | 0.5% | 6.4% |
| Breast cancer | 2002 | 459 | 30486 | 1.5% | 1.4% | 1.6% | 0.7% | 0.6% | 6.5% |
| Breast cancer | 2003 | 613 | 41024 | 1.5% | 1.4% | 1.6% | 0.7% | 0.6% | 6.5% |
| Breast cancer | 2004 | 771 | 50006 | 1.5% | 1.4% | 1.7% | 0.7% | 0.6% | 24.5% |
| Breast cancer | 2005 | 891 | 58130 | 1.5% | 1.4% | 1.6% | 0.9% | 0.9% | 1.2% |
| Breast cancer | 2006 | 1055 | 65885 | 1.6% | 1.5% | 1.7% | 1.0% | 0.9% | 1.2% |
| Breast cancer | 2007 | 1168 | 73097 | 1.6% | 1.5% | 1.7% | 1.0% | 0.9% | 1.2% |
| Breast cancer | 2008 | 1287 | 78805 | 1.6% | 1.5% | 1.7% | 1.0% | 0.9% | 1.2% |
| Breast cancer | 2009 | 1420 | 84549 | 1.7% | 1.6% | 1.8% | 1.0% | 1.0% | 1.2% |
| Breast cancer | 2010 | 1522 | 87915 | 1.7% | 1.6% | 1.8% | 0.7% | 0.6% | 12.5% |
| Breast cancer | 2011 | 1573 | 90287 | 1.7% | 1.7% | 1.8% | 0.7% | 0.6% | 12.5% |
| Breast cancer | 2012 | 1596 | 91279 | 1.7% | 1.7% | 1.8% | 0.7% | 0.7% | 24.5% |
| Breast cancer | 2013 | 1524 | 87289 | 1.7% | 1.7% | 1.8% | 0.7% | 0.7% | 24.5% |
| Breast cancer | 2014 | 1418 | 76727 | 1.8% | 1.8% | 1.9% | 0.7% | 0.7% | 8.5% |
| Cardiac arrhythmia | 2000 | 342.5 | 3234 | 10.6% | 9.6% | 11.7% | 6.7% | 5.5% | 19.5% |
| Cardiac arrhythmia | 2001 | 989.5 | 9424.5 | 10.5% | 9.9% | 11.1% | 7.5% | 4.3% | 19.9% |
| Cardiac arrhythmia | 2002 | 1643.5 | 15243 | 10.8% | 10.3% | 11.3% | 9.6% | 4.4% | 27.5% |
| Cardiac arrhythmia | 2003 | 2198.5 | 20512 | 10.7% | 10.3% | 11.1% | 7.4% | 6.4% | 10.6% |
| Cardiac arrhythmia | 2004 | 2769 | 25003 | 11.1% | 10.7% | 11.5% | 7.3% | 6.5% | 18.9% |
| Cardiac arrhythmia | 2005 | 3297.5 | 29065 | 11.4% | 11.0% | 11.7% | 8.8% | 8.2% | 9.7% |
| Cardiac arrhythmia | 2006 | 3884 | 32942.5 | 11.8% | 11.4% | 12.1% | 9.0% | 8.4% | 9.9% |
| Cardiac arrhythmia | 2007 | 4444 | 36548.5 | 12.2% | 11.8% | 12.5% | 9.3% | 8.8% | 10.1% |
| Cardiac arrhythmia | 2008 | 4967.5 | 39402.5 | 12.7% | 12.3% | 12.9% | 9.9% | 9.3% | 10.7% |
| Cardiac arrhythmia | 2009 | 5570 | 42274.5 | 13.2% | 12.9% | 13.5% | 10.4% | 9.7% | 11.2% |
| Cardiac arrhythmia | 2010 | 6016 | 43957.5 | 13.8% | 13.4% | 14.0% | 9.1% | 8.4% | 14.8% |
| Cardiac arrhythmia | 2011 | 6445.5 | 45143.5 | 14.4% | 14.0% | 14.6% | 8.3% | 7.3% | 30.2% |
| Cardiac arrhythmia | 2012 | 6772.5 | 45639.5 | 14.9% | 14.5% | 15.2% | 10.2% | 9.3% | 21.6% |
| Cardiac arrhythmia | 2013 | 6669 | 43644.5 | 15.4% | 14.9% | 15.6% | 10.9% | 9.8% | 22.2% |
| Cardiac arrhythmia | 2014 | 6037 | 38363.5 | 15.8% | 15.4% | 16.1% | 10.8% | 9.8% | 14.8% |
| Cervical cancer | 2000 | 6 | 6468 | 0.1% | 0.0% | 0.2% | 0.0% | 0.0% | 24.2% |
| Cervical cancer | 2001 | 13 | 18849 | 0.1% | 0.0% | 0.1% | 0.0% | 0.0% | 6.1% |
| Cervical cancer | 2002 | 27 | 30486 | 0.1% | 0.1% | 0.1% | 0.1% | 0.0% | 6.1% |
| Cervical cancer | 2003 | 44 | 41024 | 0.1% | 0.1% | 0.1% | 0.1% | 0.1% | 6.1% |
| Cervical cancer | 2004 | 54 | 50006 | 0.1% | 0.1% | 0.1% | 0.1% | 0.1% | 24.2% |
| Cervical cancer | 2005 | 65 | 58130 | 0.1% | 0.1% | 0.1% | 0.2% | 0.1% | 0.4% |
| Cervical cancer | 2006 | 79 | 65885 | 0.1% | 0.1% | 0.1% | 0.2% | 0.1% | 0.4% |
| Cervical cancer | 2007 | 90 | 73097 | 0.1% | 0.1% | 0.2% | 0.2% | 0.1% | 0.5% |
| Cervical cancer | 2008 | 105 | 78805 | 0.1% | 0.1% | 0.2% | 0.2% | 0.1% | 0.4% |
| Cervical cancer | 2009 | 120 | 84549 | 0.1% | 0.1% | 0.2% | 0.2% | 0.1% | 0.4% |
| Cervical cancer | 2010 | 124 | 87915 | 0.1% | 0.1% | 0.2% | 0.2% | 0.1% | 12.2% |
| Cervical cancer | 2011 | 129 | 90287 | 0.1% | 0.1% | 0.2% | 0.2% | 0.1% | 12.2% |
| Cervical cancer | 2012 | 141 | 91279 | 0.2% | 0.1% | 0.2% | 0.2% | 0.2% | 24.3% |
| Cervical cancer | 2013 | 135 | 87289 | 0.2% | 0.1% | 0.2% | 0.3% | 0.2% | 24.3% |
| Cervical cancer | 2014 | 136 | 76727 | 0.2% | 0.1% | 0.2% | 0.4% | 0.1% | 8.3% |
| Chronic kidney disease | 2000 | 36.5 | 3234 | 1.1% | 0.8% | 1.6% | 1.6% | 0.5% | 15.5% |
| Chronic kidney disease | 2001 | 119.5 | 9424.5 | 1.3% | 1.1% | 1.5% | 2.7% | 1.0% | 14.5% |
| Chronic kidney disease | 2002 | 194.5 | 15243 | 1.3% | 1.1% | 1.5% | 2.1% | 1.1% | 17.5% |
| Chronic kidney disease | 2003 | 274.5 | 20512 | 1.3% | 1.2% | 1.5% | 2.0% | 1.2% | 5.5% |
| Chronic kidney disease | 2004 | 322 | 25003 | 1.3% | 1.2% | 1.4% | 1.7% | 1.1% | 13.9% |
| Chronic kidney disease | 2005 | 397.5 | 29065 | 1.4% | 1.2% | 1.5% | 1.6% | 1.3% | 2.3% |
| Chronic kidney disease | 2006 | 521.5 | 32942.5 | 1.6% | 1.5% | 1.7% | 1.8% | 1.5% | 2.4% |
| Chronic kidney disease | 2007 | 1152 | 36548.5 | 3.2% | 3.0% | 3.3% | 3.0% | 2.5% | 3.6% |
| Chronic kidney disease | 2008 | 1852 | 39402.5 | 4.8% | 4.5% | 4.9% | 4.0% | 3.5% | 4.6% |
| Chronic kidney disease | 2009 | 2632 | 42274.5 | 6.3% | 6.0% | 6.5% | 4.9% | 4.5% | 5.5% |
| Chronic kidney disease | 2010 | 3222.5 | 43957.5 | 7.5% | 7.1% | 7.6% | 4.8% | 4.3% | 10.7% |
| Chronic kidney disease | 2011 | 3765.5 | 45143.5 | 8.5% | 8.1% | 8.6% | 4.3% | 3.8% | 27.0% |
| Chronic kidney disease | 2012 | 4191.5 | 45639.5 | 9.4% | 8.9% | 9.5% | 5.8% | 5.3% | 17.5% |
| Chronic kidney disease | 2013 | 4298 | 43644.5 | 10.1% | 9.6% | 10.1% | 6.0% | 5.4% | 17.5% |
| Chronic kidney disease | 2014 | 3924 | 38363.5 | 10.5% | 9.9% | 10.5% | 6.6% | 5.8% | 10.6% |
| Colon cancer | 2000 | 19 | 3234 | 0.6% | 0.4% | 0.9% | 0.3% | 0.2% | 14.0% |
| Colon cancer | 2001 | 72 | 9424.5 | 0.8% | 0.6% | 1.0% | 2.0% | 0.2% | 15.4% |
| Colon cancer | 2002 | 105 | 15243 | 0.7% | 0.6% | 0.8% | 3.6% | 0.2% | 22.4% |
| Colon cancer | 2003 | 143 | 20512 | 0.7% | 0.6% | 0.8% | 0.5% | 0.3% | 4.0% |
| Colon cancer | 2004 | 169.5 | 25003 | 0.7% | 0.6% | 0.8% | 0.5% | 0.2% | 12.7% |
| Colon cancer | 2005 | 207.5 | 29065 | 0.7% | 0.6% | 0.8% | 0.5% | 0.3% | 1.1% |
| Colon cancer | 2006 | 241 | 32942.5 | 0.7% | 0.6% | 0.8% | 0.5% | 0.4% | 1.1% |
| Colon cancer | 2007 | 269.5 | 36548.5 | 0.7% | 0.7% | 0.8% | 0.4% | 0.4% | 0.9% |
| Colon cancer | 2008 | 298.5 | 39402.5 | 0.8% | 0.7% | 0.8% | 0.5% | 0.4% | 0.9% |
| Colon cancer | 2009 | 324 | 42274.5 | 0.8% | 0.7% | 0.9% | 0.5% | 0.4% | 0.8% |
| Colon cancer | 2010 | 341 | 43957.5 | 0.8% | 0.7% | 0.9% | 0.4% | 0.3% | 6.5% |
| Colon cancer | 2011 | 361.5 | 45143.5 | 0.8% | 0.7% | 0.9% | 0.3% | 0.2% | 24.3% |
| Colon cancer | 2012 | 375 | 45639.5 | 0.8% | 0.7% | 0.9% | 0.4% | 0.3% | 12.6% |
| Colon cancer | 2013 | 365 | 43644.5 | 0.8% | 0.8% | 0.9% | 0.4% | 0.3% | 12.6% |
| Colon cancer | 2014 | 318 | 38363.5 | 0.8% | 0.7% | 0.9% | 0.4% | 0.3% | 4.6% |
| Chronic obstructive pulmonary disease | 2000 | 238.5 | 3234 | 7.4% | 6.5% | 8.3% | 4.4% | 3.6% | 17.4% |
| Chronic obstructive pulmonary disease | 2001 | 654.5 | 9424.5 | 6.9% | 6.4% | 7.5% | 3.5% | 3.0% | 14.5% |
| Chronic obstructive pulmonary disease | 2002 | 1029.5 | 15243 | 6.8% | 6.4% | 7.2% | 3.3% | 3.0% | 18.4% |
| Chronic obstructive pulmonary disease | 2003 | 1307 | 20512 | 6.4% | 6.0% | 6.7% | 3.6% | 3.3% | 6.8% |
| Chronic obstructive pulmonary disease | 2004 | 1575 | 25003 | 6.3% | 6.0% | 6.6% | 3.9% | 3.5% | 15.7% |
| Chronic obstructive pulmonary disease | 2005 | 1770 | 29065 | 6.1% | 5.8% | 6.4% | 4.3% | 3.9% | 4.9% |
| Chronic obstructive pulmonary disease | 2006 | 2006.5 | 32942.5 | 6.1% | 5.8% | 6.4% | 4.2% | 3.9% | 4.8% |
| Chronic obstructive pulmonary disease | 2007 | 2297.5 | 36548.5 | 6.3% | 6.0% | 6.5% | 4.3% | 4.0% | 4.9% |
| Chronic obstructive pulmonary disease | 2008 | 2537.5 | 39402.5 | 6.4% | 6.2% | 6.7% | 4.4% | 4.1% | 4.9% |
| Chronic obstructive pulmonary disease | 2009 | 2757.5 | 42274.5 | 6.5% | 6.3% | 6.8% | 4.4% | 4.2% | 4.9% |
| Chronic obstructive pulmonary disease | 2010 | 2930 | 43957.5 | 6.7% | 6.4% | 6.9% | 4.0% | 3.6% | 9.8% |
| Chronic obstructive pulmonary disease | 2011 | 3090 | 45143.5 | 6.9% | 6.6% | 7.1% | 3.5% | 3.1% | 26.4% |
| Chronic obstructive pulmonary disease | 2012 | 3217 | 45639.5 | 7.1% | 6.8% | 7.3% | 4.1% | 3.8% | 15.8% |
| Chronic obstructive pulmonary disease | 2013 | 3141 | 43644.5 | 7.2% | 7.0% | 7.4% | 4.3% | 4.1% | 16.0% |
| Chronic obstructive pulmonary disease | 2014 | 2763 | 38363.5 | 7.2% | 6.9% | 7.5% | 4.2% | 4.0% | 8.1% |
| Connective tissue disease | 2000 | 99 | 3234 | 3.1% | 2.5% | 3.7% | 2.1% | 1.5% | 15.5% |
| Connective tissue disease | 2001 | 284.5 | 9424.5 | 3.1% | 2.7% | 3.4% | 1.7% | 1.3% | 13.2% |
| Connective tissue disease | 2002 | 478 | 15243 | 3.2% | 2.9% | 3.4% | 2.0% | 1.4% | 17.4% |
| Connective tissue disease | 2003 | 663.5 | 20512 | 3.3% | 3.0% | 3.5% | 2.4% | 1.8% | 5.8% |
| Connective tissue disease | 2004 | 812 | 25003 | 3.3% | 3.0% | 3.5% | 2.1% | 1.6% | 14.0% |
| Connective tissue disease | 2005 | 970 | 29065 | 3.4% | 3.1% | 3.6% | 2.5% | 2.2% | 3.0% |
| Connective tissue disease | 2006 | 1130 | 32942.5 | 3.5% | 3.2% | 3.6% | 2.5% | 2.3% | 3.1% |
| Connective tissue disease | 2007 | 1273 | 36548.5 | 3.6% | 3.3% | 3.7% | 2.7% | 2.4% | 3.2% |
| Connective tissue disease | 2008 | 1396.5 | 39402.5 | 3.7% | 3.4% | 3.7% | 2.7% | 2.5% | 3.2% |
| Connective tissue disease | 2009 | 1548 | 42274.5 | 3.8% | 3.5% | 3.8% | 2.7% | 2.5% | 3.1% |
| Connective tissue disease | 2010 | 1639 | 43957.5 | 3.9% | 3.6% | 3.9% | 2.6% | 2.3% | 8.8% |
| Connective tissue disease | 2011 | 1757 | 45143.5 | 4.1% | 3.7% | 4.1% | 2.5% | 2.0% | 25.7% |
| Connective tissue disease | 2012 | 1813 | 45639.5 | 4.2% | 3.8% | 4.2% | 2.9% | 2.6% | 15.1% |
| Connective tissue disease | 2013 | 1760.5 | 43644.5 | 4.3% | 3.9% | 4.2% | 2.6% | 2.3% | 14.2% |
| Connective tissue disease | 2014 | 1598.5 | 38363.5 | 4.4% | 4.0% | 4.4% | 2.8% | 2.5% | 6.9% |
| Dementia | 2000 | 75 | 3234 | 2.3% | 1.9% | 2.9% | 1.1% | 0.9% | 14.7% |
| Dementia | 2001 | 179.5 | 9424.5 | 1.9% | 1.6% | 2.2% | 0.6% | 0.4% | 12.4% |
| Dementia | 2002 | 267 | 15243 | 1.8% | 1.6% | 2.0% | 0.6% | 0.4% | 16.4% |
| Dementia | 2003 | 324 | 20512 | 1.6% | 1.4% | 1.8% | 0.8% | 0.7% | 4.2% |
| Dementia | 2004 | 370.5 | 25003 | 1.5% | 1.3% | 1.6% | 0.6% | 0.5% | 12.8% |
| Dementia | 2005 | 414.5 | 29065 | 1.5% | 1.3% | 1.6% | 0.9% | 0.8% | 1.4% |
| Dementia | 2006 | 448.5 | 32942.5 | 1.4% | 1.2% | 1.5% | 0.9% | 0.8% | 1.3% |
| Dementia | 2007 | 494 | 36548.5 | 1.4% | 1.2% | 1.5% | 0.9% | 0.8% | 1.3% |
| Dementia | 2008 | 538 | 39402.5 | 1.4% | 1.3% | 1.5% | 0.8% | 0.8% | 1.3% |
| Dementia | 2009 | 588.5 | 42274.5 | 1.5% | 1.3% | 1.5% | 0.9% | 0.8% | 1.3% |
| Dementia | 2010 | 622 | 43957.5 | 1.5% | 1.3% | 1.5% | 0.7% | 0.6% | 6.9% |
| Dementia | 2011 | 635.5 | 45143.5 | 1.5% | 1.3% | 1.5% | 0.4% | 0.3% | 24.4% |
| Dementia | 2012 | 681.5 | 45639.5 | 1.6% | 1.4% | 1.6% | 0.7% | 0.6% | 12.9% |
| Dementia | 2013 | 656.5 | 43644.5 | 1.6% | 1.4% | 1.6% | 0.6% | 0.5% | 12.8% |
| Dementia | 2014 | 581 | 38363.5 | 1.6% | 1.4% | 1.6% | 0.7% | 0.6% | 4.9% |
| Depression | 2000 | 460 | 3234 | 14.3% | 13.1% | 15.5% | 16.6% | 13.8% | 30.2% |
| Depression | 2001 | 1418 | 9424.5 | 15.2% | 14.3% | 15.8% | 18.4% | 14.1% | 30.6% |
| Depression | 2002 | 2345.5 | 15243 | 15.6% | 14.8% | 16.0% | 20.2% | 13.8% | 37.2% |
| Depression | 2003 | 3241.5 | 20512 | 16.0% | 15.3% | 16.3% | 18.8% | 17.3% | 22.6% |
| Depression | 2004 | 4028 | 25003 | 16.4% | 15.7% | 16.6% | 19.6% | 17.8% | 30.7% |
| Depression | 2005 | 4811 | 29065 | 16.9% | 16.1% | 17.0% | 20.3% | 19.0% | 21.8% |
| Depression | 2006 | 5600 | 32942.5 | 17.4% | 16.6% | 17.4% | 21.0% | 19.7% | 22.5% |
| Depression | 2007 | 6327 | 36548.5 | 17.7% | 16.9% | 17.7% | 21.0% | 19.8% | 22.4% |
| Depression | 2008 | 6988.5 | 39402.5 | 18.2% | 17.4% | 18.1% | 22.0% | 20.8% | 23.4% |
| Depression | 2009 | 7624.5 | 42274.5 | 18.6% | 17.7% | 18.4% | 22.4% | 21.2% | 23.8% |
| Depression | 2010 | 8047.5 | 43957.5 | 18.9% | 17.9% | 18.7% | 22.7% | 21.2% | 28.8% |
| Depression | 2011 | 8438.5 | 45143.5 | 19.3% | 18.3% | 19.1% | 22.4% | 20.5% | 43.4% |
| Depression | 2012 | 8764.5 | 45639.5 | 19.9% | 18.8% | 19.6% | 23.4% | 21.9% | 34.9% |
| Depression | 2013 | 8560 | 43644.5 | 20.3% | 19.2% | 20.0% | 26.6% | 20.2% | 40.5% |
| Depression | 2014 | 7655 | 38363.5 | 20.8% | 19.6% | 20.4% | 24.5% | 22.7% | 29.0% |
| Diabetes mellitus | 2000 | 365 | 3234 | 11.3% | 10.2% | 12.4% | 9.2% | 7.5% | 22.2% |
| Diabetes mellitus | 2001 | 1105.5 | 9424.5 | 11.7% | 11.1% | 12.4% | 10.8% | 7.2% | 23.2% |
| Diabetes mellitus | 2002 | 1841.5 | 15243 | 12.0% | 11.6% | 12.6% | 12.3% | 6.9% | 29.8% |
| Diabetes mellitus | 2003 | 2536.5 | 20512 | 12.3% | 11.9% | 12.8% | 10.1% | 9.1% | 13.5% |
| Diabetes mellitus | 2004 | 3186 | 25003 | 12.7% | 12.3% | 13.2% | 10.9% | 9.8% | 22.2% |
| Diabetes mellitus | 2005 | 3741.5 | 29065 | 12.8% | 12.5% | 13.3% | 11.4% | 10.7% | 12.4% |
| Diabetes mellitus | 2006 | 4263 | 32942.5 | 12.9% | 12.6% | 13.3% | 11.6% | 10.8% | 12.6% |
| Diabetes mellitus | 2007 | 4822.5 | 36548.5 | 13.1% | 12.9% | 13.5% | 11.7% | 10.9% | 12.6% |
| Diabetes mellitus | 2008 | 5228.5 | 39402.5 | 13.2% | 12.9% | 13.6% | 11.7% | 11.0% | 12.6% |
| Diabetes mellitus | 2009 | 5710 | 42274.5 | 13.4% | 13.2% | 13.8% | 11.9% | 11.2% | 12.8% |
| Diabetes mellitus | 2010 | 6024.5 | 43957.5 | 13.6% | 13.4% | 14.0% | 11.1% | 10.3% | 16.9% |
| Diabetes mellitus | 2011 | 6358.5 | 45143.5 | 14.0% | 13.8% | 14.4% | 10.7% | 9.6% | 32.3% |
| Diabetes mellitus | 2012 | 6552.5 | 45639.5 | 14.3% | 14.0% | 14.7% | 11.9% | 10.8% | 23.3% |
| Diabetes mellitus | 2013 | 6377.5 | 43644.5 | 14.5% | 14.3% | 14.9% | 12.1% | 11.0% | 23.3% |
| Diabetes mellitus | 2014 | 5687.5 | 38363.5 | 14.8% | 14.5% | 15.2% | 11.9% | 10.8% | 16.0% |
| Eating disorder | 2000 | 1.5 | 3234 | 0.0% | 0.0% | 0.2% | 0.1% | 0.0% | 13.8% |
| Eating disorder | 2001 | 7 | 9424.5 | 0.1% | 0.0% | 0.2% | 0.3% | 0.1% | 12.2% |
| Eating disorder | 2002 | 11.5 | 15243 | 0.1% | 0.0% | 0.1% | 0.2% | 0.1% | 16.2% |
| Eating disorder | 2003 | 19.5 | 20512 | 0.1% | 0.1% | 0.1% | 0.2% | 0.1% | 3.6% |
| Eating disorder | 2004 | 28.5 | 25003 | 0.1% | 0.1% | 0.2% | 0.6% | 0.2% | 12.8% |
| Eating disorder | 2005 | 35.5 | 29065 | 0.1% | 0.1% | 0.2% | 0.3% | 0.2% | 0.8% |
| Eating disorder | 2006 | 42 | 32942.5 | 0.1% | 0.1% | 0.2% | 0.5% | 0.2% | 1.1% |
| Eating disorder | 2007 | 48.5 | 36548.5 | 0.1% | 0.1% | 0.2% | 0.6% | 0.3% | 1.2% |
| Eating disorder | 2008 | 57.5 | 39402.5 | 0.2% | 0.1% | 0.2% | 0.6% | 0.4% | 1.2% |
| Eating disorder | 2009 | 64.5 | 42274.5 | 0.2% | 0.1% | 0.2% | 0.6% | 0.4% | 1.2% |
| Eating disorder | 2010 | 75.5 | 43957.5 | 0.2% | 0.1% | 0.2% | 0.6% | 0.4% | 7.0% |
| Eating disorder | 2011 | 79 | 45143.5 | 0.2% | 0.1% | 0.2% | 0.8% | 0.4% | 24.6% |
| Eating disorder | 2012 | 82.5 | 45639.5 | 0.2% | 0.1% | 0.2% | 0.6% | 0.4% | 13.0% |
| Eating disorder | 2013 | 88.5 | 43644.5 | 0.2% | 0.2% | 0.2% | 0.7% | 0.3% | 12.7% |
| Eating disorder | 2014 | 82 | 38363.5 | 0.2% | 0.2% | 0.3% | 0.6% | 0.4% | 5.0% |
| Ent cancer | 2000 | 12.5 | 3234 | 0.4% | 0.2% | 0.7% | 0.2% | 0.1% | 13.9% |
| Ent cancer | 2001 | 30 | 9424.5 | 0.3% | 0.2% | 0.5% | 0.1% | 0.1% | 12.1% |
| Ent cancer | 2002 | 49.5 | 15243 | 0.3% | 0.2% | 0.4% | 0.1% | 0.1% | 16.2% |
| Ent cancer | 2003 | 51.5 | 20512 | 0.2% | 0.2% | 0.3% | 0.1% | 0.1% | 3.5% |
| Ent cancer | 2004 | 63.5 | 25003 | 0.3% | 0.2% | 0.3% | 0.1% | 0.1% | 12.5% |
| Ent cancer | 2005 | 72.5 | 29065 | 0.2% | 0.2% | 0.3% | 0.2% | 0.1% | 0.7% |
| Ent cancer | 2006 | 82.5 | 32942.5 | 0.2% | 0.2% | 0.3% | 0.2% | 0.1% | 0.7% |
| Ent cancer | 2007 | 101.5 | 36548.5 | 0.3% | 0.2% | 0.3% | 0.2% | 0.1% | 0.6% |
| Ent cancer | 2008 | 121 | 39402.5 | 0.3% | 0.3% | 0.4% | 0.2% | 0.1% | 0.6% |
| Ent cancer | 2009 | 126.5 | 42274.5 | 0.3% | 0.3% | 0.4% | 0.2% | 0.1% | 0.6% |
| Ent cancer | 2010 | 131 | 43957.5 | 0.3% | 0.3% | 0.4% | 0.2% | 0.1% | 6.4% |
| Ent cancer | 2011 | 140 | 45143.5 | 0.3% | 0.3% | 0.4% | 0.2% | 0.1% | 24.2% |
| Ent cancer | 2012 | 138 | 45639.5 | 0.3% | 0.3% | 0.4% | 0.2% | 0.1% | 12.5% |
| Ent cancer | 2013 | 130.5 | 43644.5 | 0.3% | 0.3% | 0.4% | 0.2% | 0.1% | 12.5% |
| Ent cancer | 2014 | 123 | 38363.5 | 0.3% | 0.3% | 0.4% | 0.2% | 0.1% | 4.4% |
| Epilepsy | 2000 | 63.5 | 3234 | 2.0% | 1.5% | 2.5% | 5.1% | 1.7% | 19.9% |
| Epilepsy | 2001 | 166.5 | 9424.5 | 1.8% | 1.5% | 2.1% | 4.6% | 2.2% | 16.3% |
| Epilepsy | 2002 | 293.5 | 15243 | 1.9% | 1.7% | 2.2% | 4.7% | 2.8% | 19.9% |
| Epilepsy | 2003 | 383 | 20512 | 1.9% | 1.7% | 2.1% | 3.1% | 2.2% | 6.6% |
| Epilepsy | 2004 | 472.5 | 25003 | 1.9% | 1.7% | 2.1% | 3.2% | 2.4% | 15.0% |
| Epilepsy | 2005 | 557 | 29065 | 1.9% | 1.8% | 2.1% | 3.2% | 2.6% | 4.1% |
| Epilepsy | 2006 | 660.5 | 32942.5 | 2.0% | 1.9% | 2.2% | 3.5% | 2.9% | 4.5% |
| Epilepsy | 2007 | 750.5 | 36548.5 | 2.1% | 1.9% | 2.2% | 3.4% | 2.8% | 4.3% |
| Epilepsy | 2008 | 816 | 39402.5 | 2.1% | 1.9% | 2.2% | 3.2% | 2.6% | 4.0% |
| Epilepsy | 2009 | 905.5 | 42274.5 | 2.2% | 2.0% | 2.3% | 3.4% | 2.8% | 4.2% |
| Epilepsy | 2010 | 952 | 43957.5 | 2.2% | 2.0% | 2.3% | 5.5% | 2.7% | 13.1% |
| Epilepsy | 2011 | 1014 | 45143.5 | 2.3% | 2.1% | 2.4% | 4.3% | 3.1% | 27.0% |
| Epilepsy | 2012 | 1048 | 45639.5 | 2.3% | 2.2% | 2.4% | 4.3% | 3.4% | 16.2% |
| Epilepsy | 2013 | 1027.5 | 43644.5 | 2.4% | 2.2% | 2.5% | 4.8% | 3.7% | 16.6% |
| Epilepsy | 2014 | 887.5 | 38363.5 | 2.3% | 2.2% | 2.5% | 5.6% | 3.3% | 11.0% |
| Gout | 2000 | 142 | 3234 | 4.3% | 3.7% | 5.2% | 2.5% | 2.1% | 15.5% |
| Gout | 2001 | 426.5 | 9424.5 | 4.5% | 4.1% | 5.0% | 2.6% | 2.2% | 13.9% |
| Gout | 2002 | 719 | 15243 | 4.6% | 4.4% | 5.1% | 2.7% | 2.4% | 18.1% |
| Gout | 2003 | 972.5 | 20512 | 4.6% | 4.5% | 5.0% | 2.8% | 2.5% | 5.8% |
| Gout | 2004 | 1204.5 | 25003 | 4.7% | 4.6% | 5.1% | 3.1% | 2.8% | 15.2% |
| Gout | 2005 | 1438.5 | 29065 | 4.8% | 4.7% | 5.2% | 3.4% | 3.2% | 4.0% |
| Gout | 2006 | 1687.5 | 32942.5 | 5.0% | 4.9% | 5.4% | 3.6% | 3.3% | 4.1% |
| Gout | 2007 | 1934.5 | 36548.5 | 5.1% | 5.1% | 5.5% | 3.5% | 3.3% | 3.9% |
| Gout | 2008 | 2156 | 39402.5 | 5.2% | 5.3% | 5.7% | 3.6% | 3.4% | 4.0% |
| Gout | 2009 | 2375.5 | 42274.5 | 5.4% | 5.4% | 5.8% | 3.6% | 3.5% | 4.1% |
| Gout | 2010 | 2558 | 43957.5 | 5.5% | 5.6% | 6.0% | 3.1% | 2.8% | 8.8% |
| Gout | 2011 | 2675 | 45143.5 | 5.6% | 5.7% | 6.1% | 3.2% | 2.6% | 26.2% |
| Gout | 2012 | 2748 | 45639.5 | 5.7% | 5.8% | 6.2% | 3.3% | 3.0% | 14.9% |
| Gout | 2013 | 2709 | 43644.5 | 5.9% | 6.0% | 6.4% | 4.1% | 3.5% | 16.1% |
| Gout | 2014 | 2453.5 | 38363.5 | 6.0% | 6.2% | 6.6% | 3.7% | 3.3% | 7.6% |
| Hemiplegia | 2000 | 14 | 3234 | 0.4% | 0.3% | 0.7% | 1.1% | 0.2% | 15.1% |
| Hemiplegia | 2001 | 44 | 9424.5 | 0.5% | 0.3% | 0.6% | 2.2% | 0.2% | 15.6% |
| Hemiplegia | 2002 | 67 | 15243 | 0.4% | 0.3% | 0.6% | 3.9% | 0.3% | 22.7% |
| Hemiplegia | 2003 | 90 | 20512 | 0.4% | 0.4% | 0.5% | 0.8% | 0.5% | 4.3% |
| Hemiplegia | 2004 | 115.5 | 25003 | 0.5% | 0.4% | 0.6% | 0.8% | 0.4% | 12.9% |
| Hemiplegia | 2005 | 132.5 | 29065 | 0.5% | 0.4% | 0.5% | 0.7% | 0.4% | 1.3% |
| Hemiplegia | 2006 | 141.5 | 32942.5 | 0.4% | 0.4% | 0.5% | 0.8% | 0.4% | 1.5% |
| Hemiplegia | 2007 | 151.5 | 36548.5 | 0.4% | 0.4% | 0.5% | 0.6% | 0.4% | 1.2% |
| Hemiplegia | 2008 | 156.5 | 39402.5 | 0.4% | 0.3% | 0.5% | 0.6% | 0.4% | 1.0% |
| Hemiplegia | 2009 | 164.5 | 42274.5 | 0.4% | 0.3% | 0.5% | 0.8% | 0.5% | 1.3% |
| Hemiplegia | 2010 | 162 | 43957.5 | 0.4% | 0.3% | 0.4% | 2.4% | 0.4% | 10.1% |
| Hemiplegia | 2011 | 168 | 45143.5 | 0.4% | 0.3% | 0.4% | 0.9% | 0.4% | 24.6% |
| Hemiplegia | 2012 | 169.5 | 45639.5 | 0.4% | 0.3% | 0.4% | 0.9% | 0.4% | 13.1% |
| Hemiplegia | 2013 | 166.5 | 43644.5 | 0.4% | 0.3% | 0.4% | 1.0% | 0.5% | 13.2% |
| Hemiplegia | 2014 | 143 | 38363.5 | 0.4% | 0.3% | 0.4% | 1.0% | 0.5% | 5.3% |
| Heart failure | 2000 | 335.5 | 3234 | 10.4% | 9.4% | 11.5% | 6.1% | 5.1% | 18.9% |
| Heart failure | 2001 | 964.5 | 9424.5 | 10.3% | 9.6% | 10.9% | 5.0% | 4.0% | 15.9% |
| Heart failure | 2002 | 1472 | 15243 | 9.7% | 9.2% | 10.1% | 4.9% | 4.2% | 19.7% |
| Heart failure | 2003 | 1868 | 20512 | 9.1% | 8.7% | 9.5% | 6.0% | 5.2% | 9.2% |
| Heart failure | 2004 | 2175.5 | 25003 | 8.7% | 8.4% | 9.1% | 5.5% | 5.0% | 17.4% |
| Heart failure | 2005 | 2403 | 29065 | 8.3% | 8.0% | 8.6% | 6.4% | 5.9% | 7.2% |
| Heart failure | 2006 | 2612.5 | 32942.5 | 8.0% | 7.6% | 8.2% | 6.4% | 5.8% | 7.2% |
| Heart failure | 2007 | 2805 | 36548.5 | 7.7% | 7.4% | 8.0% | 6.3% | 5.7% | 7.2% |
| Heart failure | 2008 | 2911 | 39402.5 | 7.4% | 7.1% | 7.7% | 6.1% | 5.5% | 6.8% |
| Heart failure | 2009 | 3117 | 42274.5 | 7.4% | 7.1% | 7.6% | 6.1% | 5.6% | 6.8% |
| Heart failure | 2010 | 3254 | 43957.5 | 7.4% | 7.2% | 7.7% | 5.5% | 4.8% | 11.3% |
| Heart failure | 2011 | 3349 | 45143.5 | 7.4% | 7.2% | 7.7% | 5.0% | 4.1% | 27.5% |
| Heart failure | 2012 | 3402.5 | 45639.5 | 7.5% | 7.2% | 7.7% | 5.5% | 4.8% | 17.0% |
| Heart failure | 2013 | 3269.5 | 43644.5 | 7.5% | 7.2% | 7.7% | 5.9% | 5.1% | 17.5% |
| Heart failure | 2014 | 2904.5 | 38363.5 | 7.6% | 7.3% | 7.8% | 6.2% | 5.3% | 10.3% |
| Hiv/aids | 2000 | 45 | 3234 | 1.4% | 1.0% | 1.9% | 0.9% | 0.6% | 14.4% |
| Hiv/aids | 2001 | 155 | 9424.5 | 1.6% | 1.4% | 1.9% | 1.0% | 0.8% | 12.7% |
| Hiv/aids | 2002 | 252.5 | 15243 | 1.7% | 1.5% | 1.9% | 1.0% | 0.8% | 16.7% |
| Hiv/aids | 2003 | 332 | 20512 | 1.6% | 1.5% | 1.8% | 1.1% | 0.9% | 4.3% |
| Hiv/aids | 2004 | 417.5 | 25003 | 1.7% | 1.5% | 1.8% | 1.0% | 0.9% | 13.2% |
| Hiv/aids | 2005 | 471 | 29065 | 1.6% | 1.5% | 1.8% | 1.2% | 1.0% | 1.8% |
| Hiv/aids | 2006 | 535.5 | 32942.5 | 1.6% | 1.5% | 1.8% | 1.2% | 1.0% | 1.7% |
| Hiv/aids | 2007 | 593 | 36548.5 | 1.6% | 1.5% | 1.8% | 1.2% | 1.0% | 1.7% |
| Hiv/aids | 2008 | 655 | 39402.5 | 1.7% | 1.5% | 1.8% | 1.3% | 1.1% | 1.7% |
| Hiv/aids | 2009 | 705.5 | 42274.5 | 1.7% | 1.6% | 1.8% | 1.2% | 1.1% | 1.6% |
| Hiv/aids | 2010 | 712 | 43957.5 | 1.6% | 1.5% | 1.7% | 1.2% | 0.9% | 7.2% |
| Hiv/aids | 2011 | 722.5 | 45143.5 | 1.6% | 1.5% | 1.7% | 1.0% | 0.8% | 24.7% |
| Hiv/aids | 2012 | 736 | 45639.5 | 1.6% | 1.5% | 1.7% | 1.2% | 1.0% | 13.4% |
| Hiv/aids | 2013 | 710 | 43644.5 | 1.6% | 1.5% | 1.7% | 1.1% | 0.9% | 13.2% |
| Hiv/aids | 2014 | 608 | 38363.5 | 1.6% | 1.5% | 1.7% | 1.0% | 0.9% | 5.2% |
| Hyperlipidaemia | 2000 | 327.5 | 3234 | 10.1% | 9.1% | 11.2% | 10.5% | 8.4% | 23.6% |
| Hyperlipidaemia | 2001 | 1039 | 9424.5 | 11.0% | 10.4% | 11.7% | 10.3% | 8.6% | 21.0% |
| Hyperlipidaemia | 2002 | 1804.5 | 15243 | 11.8% | 11.3% | 12.4% | 10.6% | 9.2% | 25.0% |
| Hyperlipidaemia | 2003 | 2660.5 | 20512 | 13.0% | 12.5% | 13.4% | 12.4% | 11.0% | 15.8% |
| Hyperlipidaemia | 2004 | 3495 | 25003 | 14.0% | 13.6% | 14.4% | 12.4% | 11.4% | 23.7% |
| Hyperlipidaemia | 2005 | 4373.5 | 29065 | 15.0% | 14.6% | 15.5% | 13.5% | 12.7% | 14.5% |
| Hyperlipidaemia | 2006 | 5438.5 | 32942.5 | 16.5% | 16.1% | 16.9% | 14.8% | 13.9% | 15.8% |
| Hyperlipidaemia | 2007 | 6572 | 36548.5 | 18.0% | 17.6% | 18.4% | 15.5% | 14.8% | 16.5% |
| Hyperlipidaemia | 2008 | 7663 | 39402.5 | 19.4% | 19.1% | 19.8% | 16.5% | 15.8% | 17.5% |
| Hyperlipidaemia | 2009 | 8866 | 42274.5 | 21.0% | 20.6% | 21.4% | 17.6% | 16.8% | 18.5% |
| Hyperlipidaemia | 2010 | 9895.5 | 43957.5 | 22.5% | 22.1% | 22.9% | 17.8% | 16.9% | 23.4% |
| Hyperlipidaemia | 2011 | 10837.5 | 45143.5 | 24.0% | 23.6% | 24.4% | 17.4% | 16.3% | 38.5% |
| Hyperlipidaemia | 2012 | 11499.5 | 45639.5 | 25.2% | 24.8% | 25.6% | 18.8% | 18.0% | 29.8% |
| Hyperlipidaemia | 2013 | 11625 | 43644.5 | 26.6% | 26.2% | 27.1% | 19.2% | 18.5% | 30.2% |
| Hyperlipidaemia | 2014 | 10637.5 | 38363.5 | 27.7% | 27.3% | 28.2% | 20.6% | 19.4% | 24.6% |
| Hypertension | 2000 | 1310 | 3234 | 40.6% | 38.8% | 42.2% | 32.0% | 26.9% | 45.9% |
| Hypertension | 2001 | 3941.5 | 9424.5 | 42.0% | 40.8% | 42.8% | 31.1% | 26.3% | 43.2% |
| Hypertension | 2002 | 6608.5 | 15243 | 43.5% | 42.6% | 44.1% | 33.4% | 26.5% | 50.3% |
| Hypertension | 2003 | 9144 | 20512 | 44.8% | 43.9% | 45.3% | 33.5% | 31.7% | 37.3% |
| Hypertension | 2004 | 11454.5 | 25003 | 46.1% | 45.2% | 46.4% | 32.7% | 31.0% | 43.6% |
| Hypertension | 2005 | 13618 | 29065 | 47.2% | 46.3% | 47.4% | 36.9% | 35.6% | 38.3% |
| Hypertension | 2006 | 15780 | 32942.5 | 48.2% | 47.4% | 48.4% | 37.1% | 36.0% | 38.5% |
| Hypertension | 2007 | 17823.5 | 36548.5 | 49.2% | 48.3% | 49.3% | 37.3% | 36.3% | 38.6% |
| Hypertension | 2008 | 19481.5 | 39402.5 | 49.9% | 48.9% | 49.9% | 37.7% | 36.6% | 38.8% |
| Hypertension | 2009 | 21232 | 42274.5 | 50.7% | 49.7% | 50.7% | 37.9% | 36.9% | 39.0% |
| Hypertension | 2010 | 22301 | 43957.5 | 51.2% | 50.3% | 51.2% | 34.8% | 33.6% | 40.5% |
| Hypertension | 2011 | 23158 | 45143.5 | 51.8% | 50.8% | 51.8% | 30.8% | 29.5% | 51.4% |
| Hypertension | 2012 | 23684 | 45639.5 | 52.4% | 51.4% | 52.4% | 35.2% | 34.1% | 46.1% |
| Hypertension | 2013 | 22831.5 | 43644.5 | 52.8% | 51.8% | 52.8% | 35.2% | 33.8% | 46.0% |
| Hypertension | 2014 | 20126 | 38363.5 | 53.0% | 52.0% | 53.0% | 35.8% | 34.4% | 40.1% |
| Learning disability | 2000 | 7 | 3234 | 0.2% | 0.1% | 0.4% | 0.4% | 0.1% | 14.2% |
| Learning disability | 2001 | 20.5 | 9424.5 | 0.2% | 0.1% | 0.3% | 1.2% | 0.1% | 13.2% |
| Learning disability | 2002 | 34 | 15243 | 0.2% | 0.2% | 0.3% | 0.7% | 0.2% | 16.5% |
| Learning disability | 2003 | 54.5 | 20512 | 0.3% | 0.2% | 0.3% | 0.5% | 0.2% | 4.0% |
| Learning disability | 2004 | 60 | 25003 | 0.2% | 0.2% | 0.3% | 0.6% | 0.2% | 12.8% |
| Learning disability | 2005 | 74.5 | 29065 | 0.3% | 0.2% | 0.3% | 0.5% | 0.3% | 1.2% |
| Learning disability | 2006 | 97.5 | 32942.5 | 0.3% | 0.2% | 0.4% | 0.6% | 0.3% | 1.2% |
| Learning disability | 2007 | 121 | 36548.5 | 0.3% | 0.3% | 0.4% | 0.6% | 0.4% | 1.1% |
| Learning disability | 2008 | 132 | 39402.5 | 0.3% | 0.3% | 0.4% | 0.6% | 0.4% | 1.1% |
| Learning disability | 2009 | 155.5 | 42274.5 | 0.4% | 0.3% | 0.4% | 0.9% | 0.6% | 1.6% |
| Learning disability | 2010 | 164.5 | 43957.5 | 0.4% | 0.3% | 0.4% | 2.6% | 0.5% | 10.3% |
| Learning disability | 2011 | 176 | 45143.5 | 0.4% | 0.3% | 0.5% | 1.5% | 0.7% | 25.1% |
| Learning disability | 2012 | 192.5 | 45639.5 | 0.4% | 0.4% | 0.5% | 4.6% | 0.6% | 19.7% |
| Learning disability | 2013 | 203 | 43644.5 | 0.5% | 0.4% | 0.5% | 1.5% | 0.8% | 13.7% |
| Learning disability | 2014 | 171.5 | 38363.5 | 0.4% | 0.4% | 0.5% | 3.6% | 1.1% | 9.7% |
| Leukaemia | 2000 | 16 | 3234 | 0.5% | 0.3% | 0.8% | 0.3% | 0.1% | 14.0% |
| Leukaemia | 2001 | 44.5 | 9424.5 | 0.5% | 0.4% | 0.6% | 0.4% | 0.2% | 12.3% |
| Leukaemia | 2002 | 70 | 15243 | 0.5% | 0.4% | 0.6% | 0.6% | 0.1% | 16.5% |
| Leukaemia | 2003 | 95 | 20512 | 0.5% | 0.4% | 0.6% | 0.5% | 0.2% | 3.9% |
| Leukaemia | 2004 | 115 | 25003 | 0.5% | 0.4% | 0.6% | 0.6% | 0.2% | 12.7% |
| Leukaemia | 2005 | 135 | 29065 | 0.5% | 0.4% | 0.5% | 0.4% | 0.3% | 0.9% |
| Leukaemia | 2006 | 151.5 | 32942.5 | 0.5% | 0.4% | 0.5% | 0.4% | 0.3% | 0.9% |
| Leukaemia | 2007 | 169.5 | 36548.5 | 0.5% | 0.4% | 0.5% | 0.4% | 0.3% | 0.8% |
| Leukaemia | 2008 | 174.5 | 39402.5 | 0.4% | 0.4% | 0.5% | 0.4% | 0.3% | 0.8% |
| Leukaemia | 2009 | 200.5 | 42274.5 | 0.5% | 0.4% | 0.5% | 0.4% | 0.3% | 0.8% |
| Leukaemia | 2010 | 221.5 | 43957.5 | 0.5% | 0.4% | 0.6% | 0.6% | 0.3% | 6.7% |
| Leukaemia | 2011 | 239.5 | 45143.5 | 0.5% | 0.5% | 0.6% | 0.6% | 0.2% | 24.5% |
| Leukaemia | 2012 | 254.5 | 45639.5 | 0.6% | 0.5% | 0.6% | 3.7% | 0.3% | 18.8% |
| Leukaemia | 2013 | 258.5 | 43644.5 | 0.6% | 0.5% | 0.7% | 0.5% | 0.4% | 12.8% |
| Leukaemia | 2014 | 233 | 38363.5 | 0.6% | 0.5% | 0.7% | 0.6% | 0.4% | 4.8% |
| Liver disease | 2000 | 16.5 | 3234 | 0.5% | 0.3% | 0.8% | 0.5% | 0.2% | 14.1% |
| Liver disease | 2001 | 51 | 9424.5 | 0.5% | 0.4% | 0.7% | 0.6% | 0.3% | 12.4% |
| Liver disease | 2002 | 86 | 15243 | 0.6% | 0.5% | 0.7% | 0.5% | 0.4% | 16.4% |
| Liver disease | 2003 | 135 | 20512 | 0.7% | 0.6% | 0.8% | 0.6% | 0.4% | 3.9% |
| Liver disease | 2004 | 170.5 | 25003 | 0.7% | 0.6% | 0.8% | 0.7% | 0.5% | 13.0% |
| Liver disease | 2005 | 209 | 29065 | 0.7% | 0.6% | 0.8% | 0.8% | 0.6% | 1.3% |
| Liver disease | 2006 | 251.5 | 32942.5 | 0.8% | 0.7% | 0.9% | 0.9% | 0.7% | 1.4% |
| Liver disease | 2007 | 294.5 | 36548.5 | 0.8% | 0.7% | 0.9% | 0.9% | 0.7% | 1.4% |
| Liver disease | 2008 | 340.5 | 39402.5 | 0.9% | 0.8% | 1.0% | 0.9% | 0.8% | 1.4% |
| Liver disease | 2009 | 418 | 42274.5 | 1.0% | 0.9% | 1.1% | 1.1% | 0.9% | 1.5% |
| Liver disease | 2010 | 484 | 43957.5 | 1.1% | 1.0% | 1.2% | 1.1% | 0.9% | 7.2% |
| Liver disease | 2011 | 533 | 45143.5 | 1.2% | 1.1% | 1.3% | 1.3% | 0.9% | 24.9% |
| Liver disease | 2012 | 574.5 | 45639.5 | 1.3% | 1.2% | 1.4% | 1.9% | 1.3% | 14.0% |
| Liver disease | 2013 | 583.5 | 43644.5 | 1.3% | 1.2% | 1.4% | 1.8% | 1.2% | 13.9% |
| Liver disease | 2014 | 549.5 | 38363.5 | 1.4% | 1.3% | 1.6% | 1.8% | 1.2% | 6.0% |
| Liver cancer | 2000 | 0.5 | 3234 | 0.0% | 0.0% | 0.1% | 0.0% | 0.0% | 13.8% |
| Liver cancer | 2001 | 2.5 | 9424.5 | 0.0% | 0.0% | 0.1% | 0.0% | 0.0% | 12.1% |
| Liver cancer | 2002 | 2.5 | 15243 | 0.0% | 0.0% | 0.1% | 0.0% | 0.0% | 16.1% |
| Liver cancer | 2003 | 1.5 | 20512 | 0.0% | 0.0% | 0.0% | 0.0% | 0.0% | 3.5% |
| Liver cancer | 2004 | 3 | 25003 | 0.0% | 0.0% | 0.0% | 0.0% | 0.0% | 12.4% |
| Liver cancer | 2005 | 4 | 29065 | 0.0% | 0.0% | 0.0% | 0.0% | 0.0% | 0.6% |
| Liver cancer | 2006 | 3 | 32942.5 | 0.0% | 0.0% | 0.0% | 0.0% | 0.0% | 0.5% |
| Liver cancer | 2007 | 4 | 36548.5 | 0.0% | 0.0% | 0.0% | 0.0% | 0.0% | 0.5% |
| Liver cancer | 2008 | 4 | 39402.5 | 0.0% | 0.0% | 0.0% | 0.0% | 0.0% | 0.5% |
| Liver cancer | 2009 | 4.5 | 42274.5 | 0.0% | 0.0% | 0.0% | 0.0% | 0.0% | 0.4% |
| Liver cancer | 2010 | 7.5 | 43957.5 | 0.0% | 0.0% | 0.0% | 0.0% | 0.0% | 6.3% |
| Liver cancer | 2011 | 8 | 45143.5 | 0.0% | 0.0% | 0.0% | 0.0% | 0.0% | 24.2% |
| Liver cancer | 2012 | 9.5 | 45639.5 | 0.0% | 0.0% | 0.0% | 0.0% | 0.0% | 12.3% |
| Liver cancer | 2013 | 13.5 | 43644.5 | 0.0% | 0.0% | 0.1% | 0.0% | 0.0% | 12.3% |
| Liver cancer | 2014 | 10.5 | 38363.5 | 0.0% | 0.0% | 0.0% | 0.0% | 0.0% | 4.3% |
| Lung cancer | 2000 | 6.5 | 3234 | 0.2% | 0.1% | 0.4% | 0.1% | 0.0% | 13.9% |
| Lung cancer | 2001 | 18.5 | 9424.5 | 0.2% | 0.1% | 0.3% | 0.1% | 0.0% | 12.1% |
| Lung cancer | 2002 | 31 | 15243 | 0.2% | 0.1% | 0.3% | 0.1% | 0.1% | 16.1% |
| Lung cancer | 2003 | 37 | 20512 | 0.2% | 0.1% | 0.2% | 0.1% | 0.1% | 3.5% |
| Lung cancer | 2004 | 43.5 | 25003 | 0.2% | 0.1% | 0.2% | 0.1% | 0.1% | 12.4% |
| Lung cancer | 2005 | 51.5 | 29065 | 0.2% | 0.1% | 0.2% | 0.1% | 0.1% | 0.6% |
| Lung cancer | 2006 | 50.5 | 32942.5 | 0.2% | 0.1% | 0.2% | 0.1% | 0.1% | 0.6% |
| Lung cancer | 2007 | 64 | 36548.5 | 0.2% | 0.1% | 0.2% | 0.1% | 0.1% | 0.5% |
| Lung cancer | 2008 | 68 | 39402.5 | 0.2% | 0.1% | 0.2% | 0.1% | 0.1% | 0.5% |
| Lung cancer | 2009 | 69 | 42274.5 | 0.2% | 0.1% | 0.2% | 0.1% | 0.1% | 0.5% |
| Lung cancer | 2010 | 81.5 | 43957.5 | 0.2% | 0.1% | 0.2% | 0.1% | 0.1% | 6.4% |
| Lung cancer | 2011 | 84.5 | 45143.5 | 0.2% | 0.2% | 0.2% | 0.1% | 0.1% | 24.2% |
| Lung cancer | 2012 | 89 | 45639.5 | 0.2% | 0.2% | 0.2% | 0.1% | 0.1% | 12.4% |
| Lung cancer | 2013 | 87 | 43644.5 | 0.2% | 0.2% | 0.2% | 0.1% | 0.1% | 12.4% |
| Lung cancer | 2014 | 81 | 38363.5 | 0.2% | 0.2% | 0.3% | 0.2% | 0.1% | 4.5% |
| Lymphoma | 2000 | 11 | 3234 | 0.3% | 0.2% | 0.6% | 0.2% | 0.1% | 13.9% |
| Lymphoma | 2001 | 37.5 | 9424.5 | 0.4% | 0.3% | 0.5% | 0.4% | 0.2% | 12.3% |
| Lymphoma | 2002 | 63 | 15243 | 0.4% | 0.3% | 0.5% | 1.4% | 0.1% | 18.3% |
| Lymphoma | 2003 | 78 | 20512 | 0.4% | 0.3% | 0.5% | 0.5% | 0.2% | 3.8% |
| Lymphoma | 2004 | 95.5 | 25003 | 0.4% | 0.3% | 0.5% | 0.4% | 0.2% | 12.7% |
| Lymphoma | 2005 | 116.5 | 29065 | 0.4% | 0.3% | 0.5% | 0.5% | 0.3% | 1.1% |
| Lymphoma | 2006 | 143 | 32942.5 | 0.4% | 0.4% | 0.5% | 0.5% | 0.3% | 1.0% |
| Lymphoma | 2007 | 153.5 | 36548.5 | 0.4% | 0.4% | 0.5% | 0.4% | 0.3% | 0.9% |
| Lymphoma | 2008 | 174 | 39402.5 | 0.4% | 0.4% | 0.5% | 0.5% | 0.3% | 1.0% |
| Lymphoma | 2009 | 199.5 | 42274.5 | 0.5% | 0.4% | 0.5% | 0.5% | 0.3% | 1.0% |
| Lymphoma | 2010 | 221 | 43957.5 | 0.5% | 0.4% | 0.6% | 0.6% | 0.3% | 6.7% |
| Lymphoma | 2011 | 226.5 | 45143.5 | 0.5% | 0.4% | 0.6% | 0.5% | 0.2% | 24.4% |
| Lymphoma | 2012 | 242.5 | 45639.5 | 0.5% | 0.5% | 0.6% | 0.5% | 0.3% | 12.7% |
| Lymphoma | 2013 | 236.5 | 43644.5 | 0.5% | 0.5% | 0.6% | 0.4% | 0.3% | 12.7% |
| Lymphoma | 2014 | 216 | 38363.5 | 0.6% | 0.5% | 0.6% | 0.4% | 0.3% | 4.6% |
| Metastatic cancer | 2000 | 20.5 | 3234 | 0.6% | 0.4% | 1.0% | 0.4% | 0.2% | 14.1% |
| Metastatic cancer | 2001 | 50.5 | 9424.5 | 0.5% | 0.4% | 0.7% | 0.3% | 0.2% | 12.2% |
| Metastatic cancer | 2002 | 81.5 | 15243 | 0.5% | 0.4% | 0.7% | 0.3% | 0.2% | 16.3% |
| Metastatic cancer | 2003 | 111 | 20512 | 0.5% | 0.4% | 0.7% | 0.4% | 0.3% | 3.7% |
| Metastatic cancer | 2004 | 128 | 25003 | 0.5% | 0.4% | 0.6% | 0.3% | 0.2% | 12.6% |
| Metastatic cancer | 2005 | 151 | 29065 | 0.5% | 0.4% | 0.6% | 0.3% | 0.3% | 0.8% |
| Metastatic cancer | 2006 | 187.5 | 32942.5 | 0.6% | 0.5% | 0.7% | 0.4% | 0.3% | 0.9% |
| Metastatic cancer | 2007 | 203 | 36548.5 | 0.6% | 0.5% | 0.6% | 0.4% | 0.3% | 0.8% |
| Metastatic cancer | 2008 | 226 | 39402.5 | 0.6% | 0.5% | 0.7% | 0.4% | 0.3% | 0.8% |
| Metastatic cancer | 2009 | 253 | 42274.5 | 0.6% | 0.5% | 0.7% | 0.4% | 0.3% | 0.8% |
| Metastatic cancer | 2010 | 279.5 | 43957.5 | 0.7% | 0.6% | 0.7% | 0.4% | 0.3% | 6.6% |
| Metastatic cancer | 2011 | 295.5 | 45143.5 | 0.7% | 0.6% | 0.7% | 0.4% | 0.3% | 24.3% |
| Metastatic cancer | 2012 | 315.5 | 45639.5 | 0.7% | 0.6% | 0.8% | 0.5% | 0.4% | 12.7% |
| Metastatic cancer | 2013 | 312.5 | 43644.5 | 0.7% | 0.6% | 0.8% | 0.5% | 0.4% | 12.6% |
| Metastatic cancer | 2014 | 290.5 | 38363.5 | 0.8% | 0.7% | 0.8% | 0.5% | 0.4% | 4.7% |
| Obesity | 2000 | 202 | 3234 | 6.3% | 5.5% | 7.1% | 7.6% | 5.8% | 21.1% |
| Obesity | 2001 | 575 | 9424.5 | 6.1% | 5.6% | 6.6% | 7.0% | 5.2% | 18.0% |
| Obesity | 2002 | 969.5 | 15243 | 6.4% | 6.0% | 6.8% | 7.1% | 5.8% | 21.6% |
| Obesity | 2003 | 1354.5 | 20512 | 6.7% | 6.3% | 7.0% | 7.7% | 6.8% | 11.1% |
| Obesity | 2004 | 1760 | 25003 | 7.1% | 6.7% | 7.4% | 8.5% | 7.4% | 19.8% |
| Obesity | 2005 | 2192.5 | 29065 | 7.6% | 7.2% | 7.9% | 9.2% | 8.3% | 10.3% |
| Obesity | 2006 | 2639.5 | 32942.5 | 8.1% | 7.7% | 8.3% | 9.8% | 8.9% | 10.9% |
| Obesity | 2007 | 3147 | 36548.5 | 8.7% | 8.3% | 8.9% | 10.5% | 9.6% | 11.6% |
| Obesity | 2008 | 3609 | 39402.5 | 9.3% | 8.9% | 9.4% | 10.5% | 9.8% | 11.5% |
| Obesity | 2009 | 4146 | 42274.5 | 10.0% | 9.5% | 10.1% | 10.9% | 10.2% | 11.8% |
| Obesity | 2010 | 4602 | 43957.5 | 10.6% | 10.2% | 10.8% | 11.2% | 10.4% | 17.0% |
| Obesity | 2011 | 4976 | 45143.5 | 11.2% | 10.7% | 11.3% | 11.9% | 10.6% | 33.4% |
| Obesity | 2012 | 5355.5 | 45639.5 | 11.9% | 11.4% | 12.0% | 12.8% | 11.7% | 24.3% |
| Obesity | 2013 | 5475.5 | 43644.5 | 12.8% | 12.2% | 12.9% | 13.2% | 11.9% | 24.3% |
| Obesity | 2014 | 5143.5 | 38363.5 | 13.6% | 13.1% | 13.8% | 14.9% | 13.4% | 19.2% |
| Oesophageal cancer | 2000 | 2 | 3234 | 0.1% | 0.0% | 0.2% | 0.0% | 0.0% | 13.8% |
| Oesophageal cancer | 2001 | 5 | 9424.5 | 0.1% | 0.0% | 0.1% | 0.0% | 0.0% | 12.1% |
| Oesophageal cancer | 2002 | 9.5 | 15243 | 0.1% | 0.0% | 0.1% | 0.0% | 0.0% | 16.1% |
| Oesophageal cancer | 2003 | 17 | 20512 | 0.1% | 0.1% | 0.1% | 0.0% | 0.0% | 3.5% |
| Oesophageal cancer | 2004 | 13.5 | 25003 | 0.1% | 0.0% | 0.1% | 0.0% | 0.0% | 12.4% |
| Oesophageal cancer | 2005 | 14.5 | 29065 | 0.0% | 0.0% | 0.1% | 0.0% | 0.0% | 0.6% |
| Oesophageal cancer | 2006 | 18 | 32942.5 | 0.1% | 0.0% | 0.1% | 0.0% | 0.0% | 0.5% |
| Oesophageal cancer | 2007 | 25.5 | 36548.5 | 0.1% | 0.0% | 0.1% | 0.0% | 0.0% | 0.5% |
| Oesophageal cancer | 2008 | 28.5 | 39402.5 | 0.1% | 0.1% | 0.1% | 0.0% | 0.0% | 0.5% |
| Oesophageal cancer | 2009 | 28 | 42274.5 | 0.1% | 0.0% | 0.1% | 0.0% | 0.0% | 0.5% |
| Oesophageal cancer | 2010 | 32 | 43957.5 | 0.1% | 0.1% | 0.1% | 0.0% | 0.0% | 6.3% |
| Oesophageal cancer | 2011 | 37 | 45143.5 | 0.1% | 0.1% | 0.1% | 0.0% | 0.0% | 24.2% |
| Oesophageal cancer | 2012 | 37 | 45639.5 | 0.1% | 0.1% | 0.1% | 0.0% | 0.0% | 12.3% |
| Oesophageal cancer | 2013 | 32.5 | 43644.5 | 0.1% | 0.1% | 0.1% | 0.0% | 0.0% | 12.3% |
| Oesophageal cancer | 2014 | 29.5 | 38363.5 | 0.1% | 0.1% | 0.1% | 0.0% | 0.0% | 4.3% |
| Other cancer | 2000 | 46 | 3234 | 1.4% | 1.1% | 1.9% | 1.4% | 0.5% | 15.4% |
| Other cancer | 2001 | 123 | 9424.5 | 1.3% | 1.1% | 1.6% | 1.1% | 0.5% | 12.8% |
| Other cancer | 2002 | 202.5 | 15243 | 1.3% | 1.2% | 1.5% | 1.4% | 0.6% | 17.0% |
| Other cancer | 2003 | 287.5 | 20512 | 1.4% | 1.2% | 1.6% | 1.2% | 0.9% | 4.7% |
| Other cancer | 2004 | 347 | 25003 | 1.4% | 1.3% | 1.5% | 1.4% | 0.9% | 13.4% |
| Other cancer | 2005 | 422.5 | 29065 | 1.5% | 1.3% | 1.6% | 1.4% | 1.1% | 2.0% |
| Other cancer | 2006 | 516.5 | 32942.5 | 1.6% | 1.4% | 1.7% | 1.5% | 1.2% | 2.0% |
| Other cancer | 2007 | 590 | 36548.5 | 1.6% | 1.5% | 1.7% | 1.5% | 1.2% | 2.0% |
| Other cancer | 2008 | 662 | 39402.5 | 1.7% | 1.6% | 1.8% | 1.5% | 1.3% | 2.1% |
| Other cancer | 2009 | 739 | 42274.5 | 1.8% | 1.6% | 1.9% | 1.6% | 1.3% | 2.1% |
| Other cancer | 2010 | 833 | 43957.5 | 1.9% | 1.8% | 2.0% | 2.0% | 1.4% | 8.0% |
| Other cancer | 2011 | 889.5 | 45143.5 | 2.0% | 1.8% | 2.1% | 2.0% | 1.3% | 25.4% |
| Other cancer | 2012 | 942.5 | 45639.5 | 2.1% | 1.9% | 2.2% | 2.0% | 1.4% | 14.0% |
| Other cancer | 2013 | 960.5 | 43644.5 | 2.2% | 2.1% | 2.3% | 2.1% | 1.6% | 14.1% |
| Other cancer | 2014 | 904 | 38363.5 | 2.4% | 2.2% | 2.5% | 2.7% | 1.8% | 7.0% |
| Other female reproductive cancer | 2000 | 29 | 6468 | 0.4% | 0.3% | 0.6% | 0.4% | 0.2% | 24.4% |
| Other female reproductive cancer | 2001 | 98 | 18849 | 0.5% | 0.4% | 0.6% | 2.1% | 0.2% | 9.5% |
| Other female reproductive cancer | 2002 | 172 | 30486 | 0.6% | 0.5% | 0.7% | 2.2% | 0.2% | 9.5% |
| Other female reproductive cancer | 2003 | 252 | 41024 | 0.6% | 0.5% | 0.7% | 0.8% | 0.4% | 6.6% |
| Other female reproductive cancer | 2004 | 328 | 50006 | 0.7% | 0.6% | 0.7% | 0.7% | 0.4% | 24.5% |
| Other female reproductive cancer | 2005 | 408 | 58130 | 0.7% | 0.6% | 0.8% | 0.7% | 0.6% | 1.1% |
| Other female reproductive cancer | 2006 | 491 | 65885 | 0.7% | 0.7% | 0.8% | 0.8% | 0.6% | 1.1% |
| Other female reproductive cancer | 2007 | 581 | 73097 | 0.8% | 0.7% | 0.9% | 0.8% | 0.7% | 1.0% |
| Other female reproductive cancer | 2008 | 653 | 78805 | 0.8% | 0.8% | 0.9% | 0.8% | 0.7% | 1.0% |
| Other female reproductive cancer | 2009 | 733 | 84549 | 0.9% | 0.8% | 0.9% | 0.8% | 0.7% | 1.0% |
| Other female reproductive cancer | 2010 | 826 | 87915 | 0.9% | 0.9% | 1.0% | 0.7% | 0.7% | 12.5% |
| Other female reproductive cancer | 2011 | 920 | 90287 | 1.0% | 1.0% | 1.1% | 0.8% | 0.7% | 12.5% |
| Other female reproductive cancer | 2012 | 980 | 91279 | 1.1% | 1.0% | 1.1% | 0.9% | 0.8% | 24.6% |
| Other female reproductive cancer | 2013 | 994 | 87289 | 1.1% | 1.1% | 1.2% | 1.0% | 0.8% | 24.7% |
| Other female reproductive cancer | 2014 | 927 | 76727 | 1.2% | 1.1% | 1.3% | 1.1% | 0.9% | 8.7% |
| Other gastrointestinal cancer | 2000 | 3 | 3234 | 0.1% | 0.0% | 0.3% | 0.0% | 0.0% | 13.8% |
| Other gastrointestinal cancer | 2001 | 6.5 | 9424.5 | 0.1% | 0.0% | 0.1% | 0.0% | 0.0% | 12.1% |
| Other gastrointestinal cancer | 2002 | 13.5 | 15243 | 0.1% | 0.1% | 0.2% | 0.0% | 0.0% | 16.1% |
| Other gastrointestinal cancer | 2003 | 14 | 20512 | 0.1% | 0.0% | 0.1% | 0.0% | 0.0% | 3.5% |
| Other gastrointestinal cancer | 2004 | 19 | 25003 | 0.1% | 0.0% | 0.1% | 0.1% | 0.0% | 12.4% |
| Other gastrointestinal cancer | 2005 | 27.5 | 29065 | 0.1% | 0.1% | 0.1% | 0.1% | 0.0% | 0.6% |
| Other gastrointestinal cancer | 2006 | 32 | 32942.5 | 0.1% | 0.1% | 0.1% | 0.1% | 0.0% | 0.6% |
| Other gastrointestinal cancer | 2007 | 38.5 | 36548.5 | 0.1% | 0.1% | 0.1% | 0.1% | 0.1% | 0.5% |
| Other gastrointestinal cancer | 2008 | 42 | 39402.5 | 0.1% | 0.1% | 0.1% | 0.1% | 0.1% | 0.5% |
| Other gastrointestinal cancer | 2009 | 48.5 | 42274.5 | 0.1% | 0.1% | 0.2% | 0.1% | 0.1% | 0.5% |
| Other gastrointestinal cancer | 2010 | 54.5 | 43957.5 | 0.1% | 0.1% | 0.2% | 0.1% | 0.1% | 6.3% |
| Other gastrointestinal cancer | 2011 | 58.5 | 45143.5 | 0.1% | 0.1% | 0.2% | 0.1% | 0.0% | 24.2% |
| Other gastrointestinal cancer | 2012 | 55.5 | 45639.5 | 0.1% | 0.1% | 0.2% | 0.1% | 0.0% | 12.4% |
| Other gastrointestinal cancer | 2013 | 61 | 43644.5 | 0.1% | 0.1% | 0.2% | 0.1% | 0.1% | 12.4% |
| Other gastrointestinal cancer | 2014 | 57.5 | 38363.5 | 0.2% | 0.1% | 0.2% | 0.1% | 0.1% | 4.4% |
| Other male reproductive cancer | 2000 | 7 | 6468 | 0.1% | 0.1% | 0.2% | 0.1% | 0.0% | 24.2% |
| Other male reproductive cancer | 2001 | 17 | 18849 | 0.1% | 0.1% | 0.1% | 0.1% | 0.0% | 6.1% |
| Other male reproductive cancer | 2002 | 24 | 30486 | 0.1% | 0.1% | 0.1% | 0.1% | 0.0% | 6.1% |
| Other male reproductive cancer | 2003 | 38 | 41024 | 0.1% | 0.1% | 0.1% | 0.1% | 0.1% | 6.1% |
| Other male reproductive cancer | 2004 | 53 | 50006 | 0.1% | 0.1% | 0.1% | 0.1% | 0.1% | 24.2% |
| Other male reproductive cancer | 2005 | 64 | 58130 | 0.1% | 0.1% | 0.1% | 0.1% | 0.1% | 0.4% |
| Other male reproductive cancer | 2006 | 75 | 65885 | 0.1% | 0.1% | 0.1% | 0.1% | 0.1% | 0.3% |
| Other male reproductive cancer | 2007 | 81 | 73097 | 0.1% | 0.1% | 0.1% | 0.1% | 0.1% | 0.3% |
| Other male reproductive cancer | 2008 | 91 | 78805 | 0.1% | 0.1% | 0.1% | 0.1% | 0.1% | 0.3% |
| Other male reproductive cancer | 2009 | 102 | 84549 | 0.1% | 0.1% | 0.1% | 0.1% | 0.1% | 0.3% |
| Other male reproductive cancer | 2010 | 113 | 87915 | 0.1% | 0.1% | 0.2% | 0.1% | 0.1% | 12.1% |
| Other male reproductive cancer | 2011 | 122 | 90287 | 0.1% | 0.1% | 0.2% | 0.1% | 0.1% | 12.1% |
| Other male reproductive cancer | 2012 | 131 | 91279 | 0.1% | 0.1% | 0.2% | 0.1% | 0.1% | 24.2% |
| Other male reproductive cancer | 2013 | 132 | 87289 | 0.2% | 0.1% | 0.2% | 0.1% | 0.1% | 24.2% |
| Other male reproductive cancer | 2014 | 121 | 76727 | 0.2% | 0.1% | 0.2% | 0.1% | 0.1% | 8.1% |
| Other respiratory cancer | 2000 | 5.5 | 3234 | 0.2% | 0.1% | 0.4% | 0.1% | 0.0% | 13.9% |
| Other respiratory cancer | 2001 | 10.5 | 9424.5 | 0.1% | 0.1% | 0.2% | 0.0% | 0.0% | 12.1% |
| Other respiratory cancer | 2002 | 15 | 15243 | 0.1% | 0.1% | 0.2% | 0.0% | 0.0% | 16.1% |
| Other respiratory cancer | 2003 | 20.5 | 20512 | 0.1% | 0.1% | 0.2% | 0.1% | 0.0% | 3.5% |
| Other respiratory cancer | 2004 | 24 | 25003 | 0.1% | 0.1% | 0.1% | 0.0% | 0.0% | 12.4% |
| Other respiratory cancer | 2005 | 27.5 | 29065 | 0.1% | 0.1% | 0.1% | 0.1% | 0.0% | 0.6% |
| Other respiratory cancer | 2006 | 34.5 | 32942.5 | 0.1% | 0.1% | 0.1% | 0.1% | 0.0% | 0.6% |
| Other respiratory cancer | 2007 | 40 | 36548.5 | 0.1% | 0.1% | 0.1% | 0.1% | 0.0% | 0.5% |
| Other respiratory cancer | 2008 | 45 | 39402.5 | 0.1% | 0.1% | 0.2% | 0.1% | 0.0% | 0.5% |
| Other respiratory cancer | 2009 | 55 | 42274.5 | 0.1% | 0.1% | 0.2% | 0.1% | 0.1% | 0.5% |
| Other respiratory cancer | 2010 | 57.5 | 43957.5 | 0.1% | 0.1% | 0.2% | 0.1% | 0.0% | 6.3% |
| Other respiratory cancer | 2011 | 60 | 45143.5 | 0.1% | 0.1% | 0.2% | 0.1% | 0.0% | 24.2% |
| Other respiratory cancer | 2012 | 62 | 45639.5 | 0.1% | 0.1% | 0.2% | 0.1% | 0.0% | 12.4% |
| Other respiratory cancer | 2013 | 62 | 43644.5 | 0.1% | 0.1% | 0.2% | 0.1% | 0.1% | 12.4% |
| Other respiratory cancer | 2014 | 57 | 38363.5 | 0.1% | 0.1% | 0.2% | 0.1% | 0.1% | 4.4% |
| Other urological cancer | 2000 | 1 | 3234 | 0.0% | 0.0% | 0.2% | 0.0% | 0.0% | 13.8% |
| Other urological cancer | 2001 | 4 | 9424.5 | 0.0% | 0.0% | 0.1% | 0.0% | 0.0% | 12.1% |
| Other urological cancer | 2002 | 7.5 | 15243 | 0.0% | 0.0% | 0.1% | 0.0% | 0.0% | 16.1% |
| Other urological cancer | 2003 | 8.5 | 20512 | 0.0% | 0.0% | 0.1% | 0.0% | 0.0% | 3.5% |
| Other urological cancer | 2004 | 13 | 25003 | 0.1% | 0.0% | 0.1% | 0.0% | 0.0% | 12.4% |
| Other urological cancer | 2005 | 18.5 | 29065 | 0.1% | 0.0% | 0.1% | 0.0% | 0.0% | 0.6% |
| Other urological cancer | 2006 | 25 | 32942.5 | 0.1% | 0.1% | 0.1% | 0.0% | 0.0% | 0.5% |
| Other urological cancer | 2007 | 31 | 36548.5 | 0.1% | 0.1% | 0.1% | 0.1% | 0.0% | 0.5% |
| Other urological cancer | 2008 | 39.5 | 39402.5 | 0.1% | 0.1% | 0.1% | 0.1% | 0.0% | 0.5% |
| Other urological cancer | 2009 | 40.5 | 42274.5 | 0.1% | 0.1% | 0.1% | 0.1% | 0.0% | 0.5% |
| Other urological cancer | 2010 | 47 | 43957.5 | 0.1% | 0.1% | 0.1% | 0.0% | 0.0% | 6.3% |
| Other urological cancer | 2011 | 46.5 | 45143.5 | 0.1% | 0.1% | 0.1% | 0.0% | 0.0% | 24.2% |
| Other urological cancer | 2012 | 49.5 | 45639.5 | 0.1% | 0.1% | 0.1% | 0.0% | 0.0% | 12.4% |
| Other urological cancer | 2013 | 56 | 43644.5 | 0.1% | 0.1% | 0.2% | 0.1% | 0.0% | 12.4% |
| Other urological cancer | 2014 | 52.5 | 38363.5 | 0.1% | 0.1% | 0.2% | 0.1% | 0.0% | 4.4% |
| Osteoporosis | 2000 | 106 | 3234 | 3.3% | 2.7% | 3.9% | 2.0% | 1.6% | 15.5% |
| Osteoporosis | 2001 | 328.5 | 9424.5 | 3.6% | 3.1% | 3.9% | 1.5% | 1.3% | 13.0% |
| Osteoporosis | 2002 | 538 | 15243 | 3.6% | 3.2% | 3.8% | 1.5% | 1.3% | 17.0% |
| Osteoporosis | 2003 | 747.5 | 20512 | 3.8% | 3.4% | 3.9% | 2.1% | 2.0% | 5.5% |
| Osteoporosis | 2004 | 932.5 | 25003 | 3.9% | 3.5% | 4.0% | 1.6% | 1.5% | 13.4% |
| Osteoporosis | 2005 | 1141 | 29065 | 4.1% | 3.7% | 4.2% | 2.4% | 2.2% | 2.9% |
| Osteoporosis | 2006 | 1353 | 32942.5 | 4.3% | 3.9% | 4.3% | 2.5% | 2.4% | 3.0% |
| Osteoporosis | 2007 | 1555.5 | 36548.5 | 4.5% | 4.1% | 4.5% | 2.8% | 2.5% | 3.3% |
| Osteoporosis | 2008 | 1746 | 39402.5 | 4.7% | 4.2% | 4.6% | 2.8% | 2.6% | 3.3% |
| Osteoporosis | 2009 | 1982 | 42274.5 | 5.0% | 4.5% | 4.9% | 3.0% | 2.8% | 3.5% |
| Osteoporosis | 2010 | 2170 | 43957.5 | 5.3% | 4.7% | 5.1% | 3.0% | 2.7% | 9.2% |
| Osteoporosis | 2011 | 2303 | 45143.5 | 5.5% | 4.9% | 5.3% | 2.2% | 1.9% | 25.5% |
| Osteoporosis | 2012 | 2444.5 | 45639.5 | 5.9% | 5.2% | 5.6% | 3.3% | 3.0% | 15.5% |
| Osteoporosis | 2013 | 2419.5 | 43644.5 | 6.1% | 5.3% | 5.8% | 2.6% | 2.3% | 14.2% |
| Osteoporosis | 2014 | 2243.5 | 38363.5 | 6.5% | 5.6% | 6.1% | 4.8% | 3.2% | 9.9% |
| Ovarian cancer | 2000 | 7 | 6468 | 0.1% | 0.1% | 0.2% | 0.1% | 0.0% | 24.2% |
| Ovarian cancer | 2001 | 18 | 18849 | 0.1% | 0.1% | 0.2% | 0.1% | 0.0% | 6.1% |
| Ovarian cancer | 2002 | 32 | 30486 | 0.1% | 0.1% | 0.1% | 0.1% | 0.0% | 6.1% |
| Ovarian cancer | 2003 | 47 | 41024 | 0.1% | 0.1% | 0.2% | 0.1% | 0.0% | 6.1% |
| Ovarian cancer | 2004 | 65 | 50006 | 0.1% | 0.1% | 0.2% | 0.1% | 0.0% | 24.2% |
| Ovarian cancer | 2005 | 76 | 58130 | 0.1% | 0.1% | 0.2% | 0.1% | 0.1% | 0.3% |
| Ovarian cancer | 2006 | 89 | 65885 | 0.1% | 0.1% | 0.2% | 0.1% | 0.1% | 0.3% |
| Ovarian cancer | 2007 | 96 | 73097 | 0.1% | 0.1% | 0.2% | 0.1% | 0.1% | 0.3% |
| Ovarian cancer | 2008 | 96 | 78805 | 0.1% | 0.1% | 0.1% | 0.1% | 0.1% | 0.3% |
| Ovarian cancer | 2009 | 102 | 84549 | 0.1% | 0.1% | 0.1% | 0.1% | 0.1% | 0.3% |
| Ovarian cancer | 2010 | 115 | 87915 | 0.1% | 0.1% | 0.2% | 0.1% | 0.0% | 12.1% |
| Ovarian cancer | 2011 | 121 | 90287 | 0.1% | 0.1% | 0.2% | 0.1% | 0.0% | 12.1% |
| Ovarian cancer | 2012 | 125 | 91279 | 0.1% | 0.1% | 0.2% | 0.1% | 0.0% | 24.2% |
| Ovarian cancer | 2013 | 116 | 87289 | 0.1% | 0.1% | 0.2% | 0.1% | 0.0% | 24.2% |
| Ovarian cancer | 2014 | 104 | 76727 | 0.1% | 0.1% | 0.2% | 0.1% | 0.0% | 8.1% |
| Peripheral arterial disease | 2000 | 218.5 | 3234 | 6.7% | 5.9% | 7.7% | 4.5% | 3.3% | 17.9% |
| Peripheral arterial disease | 2001 | 645 | 9424.5 | 6.8% | 6.4% | 7.4% | 4.1% | 2.9% | 15.3% |
| Peripheral arterial disease | 2002 | 1018 | 15243 | 6.7% | 6.3% | 7.1% | 4.0% | 3.0% | 19.1% |
| Peripheral arterial disease | 2003 | 1327.5 | 20512 | 6.4% | 6.1% | 6.8% | 4.3% | 3.6% | 7.6% |
| Peripheral arterial disease | 2004 | 1599.5 | 25003 | 6.4% | 6.1% | 6.7% | 4.3% | 3.7% | 16.1% |
| Peripheral arterial disease | 2005 | 1814.5 | 29065 | 6.2% | 6.0% | 6.5% | 4.8% | 4.3% | 5.5% |
| Peripheral arterial disease | 2006 | 2075.5 | 32942.5 | 6.3% | 6.0% | 6.6% | 4.7% | 4.3% | 5.4% |
| Peripheral arterial disease | 2007 | 2287.5 | 36548.5 | 6.2% | 6.0% | 6.5% | 4.7% | 4.2% | 5.3% |
| Peripheral arterial disease | 2008 | 2465 | 39402.5 | 6.2% | 6.0% | 6.5% | 4.8% | 4.3% | 5.4% |
| Peripheral arterial disease | 2009 | 2652 | 42274.5 | 6.2% | 6.0% | 6.5% | 5.0% | 4.5% | 5.8% |
| Peripheral arterial disease | 2010 | 2756 | 43957.5 | 6.2% | 6.0% | 6.5% | 4.4% | 3.8% | 10.3% |
| Peripheral arterial disease | 2011 | 2825.5 | 45143.5 | 6.2% | 6.0% | 6.5% | 4.0% | 3.3% | 26.7% |
| Peripheral arterial disease | 2012 | 2923 | 45639.5 | 6.3% | 6.2% | 6.6% | 4.6% | 3.9% | 16.3% |
| Peripheral arterial disease | 2013 | 2817.5 | 43644.5 | 6.4% | 6.2% | 6.7% | 4.9% | 4.2% | 16.6% |
| Peripheral arterial disease | 2014 | 2432.5 | 38363.5 | 6.2% | 6.1% | 6.6% | 4.4% | 3.7% | 8.4% |
| Pancreatic cancer | 2000 | 1.5 | 3234 | 0.0% | 0.0% | 0.2% | 0.0% | 0.0% | 13.8% |
| Pancreatic cancer | 2001 | 2.5 | 9424.5 | 0.0% | 0.0% | 0.1% | 0.0% | 0.0% | 12.1% |
| Pancreatic cancer | 2002 | 5.5 | 15243 | 0.0% | 0.0% | 0.1% | 0.0% | 0.0% | 16.1% |
| Pancreatic cancer | 2003 | 5.5 | 20512 | 0.0% | 0.0% | 0.1% | 0.0% | 0.0% | 3.5% |
| Pancreatic cancer | 2004 | 5.5 | 25003 | 0.0% | 0.0% | 0.0% | 0.0% | 0.0% | 12.4% |
| Pancreatic cancer | 2005 | 4 | 29065 | 0.0% | 0.0% | 0.0% | 0.0% | 0.0% | 0.6% |
| Pancreatic cancer | 2006 | 6 | 32942.5 | 0.0% | 0.0% | 0.0% | 0.0% | 0.0% | 0.5% |
| Pancreatic cancer | 2007 | 8 | 36548.5 | 0.0% | 0.0% | 0.0% | 0.0% | 0.0% | 0.5% |
| Pancreatic cancer | 2008 | 11.5 | 39402.5 | 0.0% | 0.0% | 0.1% | 0.0% | 0.0% | 0.5% |
| Pancreatic cancer | 2009 | 12.5 | 42274.5 | 0.0% | 0.0% | 0.1% | 0.0% | 0.0% | 0.4% |
| Pancreatic cancer | 2010 | 12.5 | 43957.5 | 0.0% | 0.0% | 0.0% | 0.0% | 0.0% | 6.3% |
| Pancreatic cancer | 2011 | 14.5 | 45143.5 | 0.0% | 0.0% | 0.1% | 0.0% | 0.0% | 24.2% |
| Pancreatic cancer | 2012 | 13.5 | 45639.5 | 0.0% | 0.0% | 0.1% | 0.0% | 0.0% | 12.3% |
| Pancreatic cancer | 2013 | 14.5 | 43644.5 | 0.0% | 0.0% | 0.1% | 0.0% | 0.0% | 12.3% |
| Pancreatic cancer | 2014 | 11 | 38363.5 | 0.0% | 0.0% | 0.1% | 0.0% | 0.0% | 4.3% |
| Prostate cancer | 2000 | 62 | 6468 | 1.0% | 0.7% | 1.2% | 0.3% | 0.2% | 24.3% |
| Prostate cancer | 2001 | 196 | 18849 | 1.0% | 0.9% | 1.2% | 0.4% | 0.3% | 6.2% |
| Prostate cancer | 2002 | 304 | 30486 | 1.0% | 0.9% | 1.1% | 0.3% | 0.3% | 6.2% |
| Prostate cancer | 2003 | 412 | 41024 | 1.0% | 0.9% | 1.1% | 0.3% | 0.3% | 6.2% |
| Prostate cancer | 2004 | 494 | 50006 | 1.0% | 0.9% | 1.1% | 0.3% | 0.3% | 24.3% |
| Prostate cancer | 2005 | 607 | 58130 | 1.0% | 1.0% | 1.1% | 0.6% | 0.5% | 0.8% |
| Prostate cancer | 2006 | 738 | 65885 | 1.1% | 1.0% | 1.2% | 0.6% | 0.6% | 0.8% |
| Prostate cancer | 2007 | 859 | 73097 | 1.2% | 1.1% | 1.3% | 0.7% | 0.6% | 0.8% |
| Prostate cancer | 2008 | 940 | 78805 | 1.2% | 1.1% | 1.3% | 0.7% | 0.6% | 0.8% |
| Prostate cancer | 2009 | 1058 | 84549 | 1.3% | 1.2% | 1.3% | 0.7% | 0.6% | 0.9% |
| Prostate cancer | 2010 | 1163 | 87915 | 1.3% | 1.2% | 1.4% | 0.4% | 0.4% | 12.3% |
| Prostate cancer | 2011 | 1240 | 90287 | 1.4% | 1.3% | 1.5% | 0.5% | 0.4% | 12.3% |
| Prostate cancer | 2012 | 1331 | 91279 | 1.5% | 1.4% | 1.5% | 0.5% | 0.4% | 24.4% |
| Prostate cancer | 2013 | 1311 | 87289 | 1.5% | 1.4% | 1.6% | 0.5% | 0.5% | 24.4% |
| Prostate cancer | 2014 | 1188 | 76727 | 1.5% | 1.5% | 1.6% | 0.5% | 0.5% | 8.3% |
| Psychoses | 2000 | 21 | 3234 | 0.7% | 0.4% | 1.0% | 0.9% | 0.3% | 14.6% |
| Psychoses | 2001 | 60.5 | 9424.5 | 0.7% | 0.5% | 0.8% | 0.5% | 0.3% | 12.4% |
| Psychoses | 2002 | 93 | 15243 | 0.6% | 0.5% | 0.7% | 0.6% | 0.4% | 16.4% |
| Psychoses | 2003 | 119.5 | 20512 | 0.6% | 0.5% | 0.7% | 0.7% | 0.5% | 4.1% |
| Psychoses | 2004 | 149.5 | 25003 | 0.6% | 0.5% | 0.7% | 0.7% | 0.5% | 12.8% |
| Psychoses | 2005 | 180.5 | 29065 | 0.6% | 0.5% | 0.7% | 0.8% | 0.5% | 1.3% |
| Psychoses | 2006 | 197 | 32942.5 | 0.6% | 0.5% | 0.7% | 0.8% | 0.6% | 1.3% |
| Psychoses | 2007 | 217 | 36548.5 | 0.6% | 0.5% | 0.7% | 0.7% | 0.5% | 1.2% |
| Psychoses | 2008 | 234 | 39402.5 | 0.6% | 0.5% | 0.7% | 0.6% | 0.5% | 1.1% |
| Psychoses | 2009 | 246 | 42274.5 | 0.6% | 0.5% | 0.7% | 0.6% | 0.5% | 1.0% |
| Psychoses | 2010 | 261 | 43957.5 | 0.6% | 0.5% | 0.7% | 0.8% | 0.5% | 6.9% |
| Psychoses | 2011 | 266.5 | 45143.5 | 0.6% | 0.5% | 0.7% | 0.9% | 0.5% | 24.7% |
| Psychoses | 2012 | 289 | 45639.5 | 0.7% | 0.6% | 0.7% | 0.8% | 0.6% | 13.0% |
| Psychoses | 2013 | 266.5 | 43644.5 | 0.6% | 0.5% | 0.7% | 4.1% | 0.5% | 19.3% |
| Psychoses | 2014 | 239.5 | 38363.5 | 0.6% | 0.6% | 0.7% | 1.0% | 0.6% | 5.2% |
| Peptic ulcer disease | 2000 | 169.5 | 3234 | 5.2% | 4.5% | 6.1% | 3.6% | 2.6% | 16.6% |
| Peptic ulcer disease | 2001 | 504.5 | 9424.5 | 5.3% | 4.9% | 5.8% | 3.2% | 2.7% | 14.3% |
| Peptic ulcer disease | 2002 | 847 | 15243 | 5.5% | 5.2% | 5.9% | 3.2% | 2.8% | 18.3% |
| Peptic ulcer disease | 2003 | 1140.5 | 20512 | 5.5% | 5.3% | 5.9% | 3.6% | 3.3% | 6.7% |
| Peptic ulcer disease | 2004 | 1384 | 25003 | 5.5% | 5.3% | 5.8% | 3.8% | 3.5% | 15.7% |
| Peptic ulcer disease | 2005 | 1594.5 | 29065 | 5.4% | 5.2% | 5.8% | 4.1% | 3.8% | 4.7% |
| Peptic ulcer disease | 2006 | 1817 | 32942.5 | 5.4% | 5.3% | 5.8% | 4.1% | 3.8% | 4.7% |
| Peptic ulcer disease | 2007 | 2016 | 36548.5 | 5.4% | 5.3% | 5.8% | 4.1% | 3.7% | 4.6% |
| Peptic ulcer disease | 2008 | 2163.5 | 39402.5 | 5.4% | 5.3% | 5.7% | 4.0% | 3.7% | 4.6% |
| Peptic ulcer disease | 2009 | 2317 | 42274.5 | 5.4% | 5.3% | 5.7% | 4.0% | 3.7% | 4.5% |
| Peptic ulcer disease | 2010 | 2396.5 | 43957.5 | 5.4% | 5.2% | 5.7% | 3.3% | 3.1% | 9.1% |
| Peptic ulcer disease | 2011 | 2435.5 | 45143.5 | 5.3% | 5.2% | 5.6% | 3.0% | 2.6% | 26.0% |
| Peptic ulcer disease | 2012 | 2452 | 45639.5 | 5.3% | 5.2% | 5.6% | 3.2% | 3.0% | 14.9% |
| Peptic ulcer disease | 2013 | 2341 | 43644.5 | 5.3% | 5.2% | 5.6% | 3.3% | 3.1% | 15.2% |
| Peptic ulcer disease | 2014 | 2050.5 | 38363.5 | 5.2% | 5.1% | 5.6% | 3.2% | 2.9% | 7.1% |
| Rectal cancer | 2000 | 14.5 | 3234 | 0.4% | 0.3% | 0.7% | 0.2% | 0.1% | 13.9% |
| Rectal cancer | 2001 | 32 | 9424.5 | 0.3% | 0.2% | 0.5% | 0.1% | 0.1% | 12.1% |
| Rectal cancer | 2002 | 59 | 15243 | 0.4% | 0.3% | 0.5% | 0.1% | 0.1% | 16.2% |
| Rectal cancer | 2003 | 75 | 20512 | 0.4% | 0.3% | 0.5% | 0.2% | 0.1% | 3.6% |
| Rectal cancer | 2004 | 96 | 25003 | 0.4% | 0.3% | 0.5% | 0.2% | 0.2% | 12.5% |
| Rectal cancer | 2005 | 115.5 | 29065 | 0.4% | 0.3% | 0.5% | 0.2% | 0.2% | 0.7% |
| Rectal cancer | 2006 | 135.5 | 32942.5 | 0.4% | 0.3% | 0.5% | 0.3% | 0.2% | 0.7% |
| Rectal cancer | 2007 | 158 | 36548.5 | 0.4% | 0.4% | 0.5% | 0.3% | 0.2% | 0.7% |
| Rectal cancer | 2008 | 169 | 39402.5 | 0.4% | 0.4% | 0.5% | 0.3% | 0.2% | 0.7% |
| Rectal cancer | 2009 | 179.5 | 42274.5 | 0.4% | 0.4% | 0.5% | 0.2% | 0.2% | 0.6% |
| Rectal cancer | 2010 | 191 | 43957.5 | 0.4% | 0.4% | 0.5% | 0.2% | 0.2% | 6.4% |
| Rectal cancer | 2011 | 198 | 45143.5 | 0.4% | 0.4% | 0.5% | 0.2% | 0.1% | 24.2% |
| Rectal cancer | 2012 | 220.5 | 45639.5 | 0.5% | 0.4% | 0.6% | 0.2% | 0.2% | 12.5% |
| Rectal cancer | 2013 | 225.5 | 43644.5 | 0.5% | 0.5% | 0.6% | 0.2% | 0.2% | 12.5% |
| Rectal cancer | 2014 | 189 | 38363.5 | 0.5% | 0.4% | 0.6% | 0.2% | 0.2% | 4.5% |
| Renal cancer | 2000 | 1.5 | 3234 | 0.0% | 0.0% | 0.2% | 0.0% | 0.0% | 13.8% |
| Renal cancer | 2001 | 7.5 | 9424.5 | 0.1% | 0.0% | 0.2% | 0.0% | 0.0% | 12.1% |
| Renal cancer | 2002 | 16.5 | 15243 | 0.1% | 0.1% | 0.2% | 0.1% | 0.0% | 16.1% |
| Renal cancer | 2003 | 23.5 | 20512 | 0.1% | 0.1% | 0.2% | 0.1% | 0.0% | 3.5% |
| Renal cancer | 2004 | 30 | 25003 | 0.1% | 0.1% | 0.2% | 0.1% | 0.0% | 12.4% |
| Renal cancer | 2005 | 36 | 29065 | 0.1% | 0.1% | 0.2% | 0.1% | 0.1% | 0.6% |
| Renal cancer | 2006 | 41 | 32942.5 | 0.1% | 0.1% | 0.2% | 0.1% | 0.1% | 0.6% |
| Renal cancer | 2007 | 53.5 | 36548.5 | 0.1% | 0.1% | 0.2% | 0.1% | 0.1% | 0.5% |
| Renal cancer | 2008 | 62 | 39402.5 | 0.2% | 0.1% | 0.2% | 0.1% | 0.1% | 0.5% |
| Renal cancer | 2009 | 68.5 | 42274.5 | 0.2% | 0.1% | 0.2% | 0.1% | 0.1% | 0.5% |
| Renal cancer | 2010 | 77 | 43957.5 | 0.2% | 0.1% | 0.2% | 0.1% | 0.1% | 6.3% |
| Renal cancer | 2011 | 81.5 | 45143.5 | 0.2% | 0.1% | 0.2% | 0.1% | 0.1% | 24.2% |
| Renal cancer | 2012 | 82.5 | 45639.5 | 0.2% | 0.1% | 0.2% | 0.1% | 0.1% | 12.4% |
| Renal cancer | 2013 | 90.5 | 43644.5 | 0.2% | 0.2% | 0.3% | 0.1% | 0.1% | 12.4% |
| Renal cancer | 2014 | 84 | 38363.5 | 0.2% | 0.2% | 0.3% | 0.1% | 0.1% | 4.4% |
| Rheumatoid arthritis | 2000 | 65 | 3234 | 2.0% | 1.6% | 2.6% | 1.4% | 1.0% | 14.9% |
| Rheumatoid arthritis | 2001 | 183.5 | 9424.5 | 2.0% | 1.7% | 2.2% | 1.2% | 0.9% | 12.8% |
| Rheumatoid arthritis | 2002 | 299.5 | 15243 | 2.0% | 1.8% | 2.2% | 1.2% | 1.0% | 16.8% |
| Rheumatoid arthritis | 2003 | 410.5 | 20512 | 2.0% | 1.8% | 2.2% | 1.4% | 1.2% | 4.7% |
| Rheumatoid arthritis | 2004 | 498.5 | 25003 | 2.0% | 1.8% | 2.2% | 1.3% | 1.1% | 13.3% |
| Rheumatoid arthritis | 2005 | 603 | 29065 | 2.1% | 1.9% | 2.2% | 1.6% | 1.4% | 2.1% |
| Rheumatoid arthritis | 2006 | 689 | 32942.5 | 2.1% | 1.9% | 2.3% | 1.6% | 1.5% | 2.1% |
| Rheumatoid arthritis | 2007 | 757.5 | 36548.5 | 2.1% | 1.9% | 2.2% | 1.6% | 1.4% | 2.0% |
| Rheumatoid arthritis | 2008 | 831 | 39402.5 | 2.2% | 2.0% | 2.3% | 1.6% | 1.5% | 2.1% |
| Rheumatoid arthritis | 2009 | 895.5 | 42274.5 | 2.2% | 2.0% | 2.3% | 1.6% | 1.5% | 2.0% |
| Rheumatoid arthritis | 2010 | 923 | 43957.5 | 2.2% | 2.0% | 2.2% | 1.6% | 1.4% | 7.7% |
| Rheumatoid arthritis | 2011 | 961 | 45143.5 | 2.2% | 2.0% | 2.3% | 1.4% | 1.2% | 25.0% |
| Rheumatoid arthritis | 2012 | 974 | 45639.5 | 2.2% | 2.0% | 2.3% | 1.6% | 1.4% | 13.8% |
| Rheumatoid arthritis | 2013 | 925 | 43644.5 | 2.2% | 2.0% | 2.3% | 1.5% | 1.3% | 13.3% |
| Rheumatoid arthritis | 2014 | 816 | 38363.5 | 2.2% | 2.0% | 2.3% | 1.5% | 1.4% | 5.7% |
| Schizophrenia | 2000 | 18.5 | 3234 | 0.6% | 0.4% | 0.9% | 0.6% | 0.2% | 14.1% |
| Schizophrenia | 2001 | 52 | 9424.5 | 0.6% | 0.4% | 0.7% | 0.5% | 0.3% | 12.4% |
| Schizophrenia | 2002 | 85.5 | 15243 | 0.6% | 0.5% | 0.7% | 0.9% | 0.4% | 16.7% |
| Schizophrenia | 2003 | 108.5 | 20512 | 0.5% | 0.4% | 0.6% | 1.0% | 0.4% | 4.5% |
| Schizophrenia | 2004 | 138.5 | 25003 | 0.6% | 0.5% | 0.7% | 0.9% | 0.4% | 12.9% |
| Schizophrenia | 2005 | 171 | 29065 | 0.6% | 0.5% | 0.7% | 0.7% | 0.5% | 1.2% |
| Schizophrenia | 2006 | 189 | 32942.5 | 0.6% | 0.5% | 0.7% | 0.8% | 0.5% | 1.3% |
| Schizophrenia | 2007 | 216.5 | 36548.5 | 0.6% | 0.5% | 0.7% | 0.7% | 0.5% | 1.2% |
| Schizophrenia | 2008 | 236 | 39402.5 | 0.6% | 0.5% | 0.7% | 0.7% | 0.6% | 1.2% |
| Schizophrenia | 2009 | 250.5 | 42274.5 | 0.6% | 0.5% | 0.7% | 0.7% | 0.6% | 1.2% |
| Schizophrenia | 2010 | 262.5 | 43957.5 | 0.6% | 0.5% | 0.7% | 0.9% | 0.6% | 7.0% |
| Schizophrenia | 2011 | 276.5 | 45143.5 | 0.6% | 0.5% | 0.7% | 1.1% | 0.6% | 24.8% |
| Schizophrenia | 2012 | 297.5 | 45639.5 | 0.7% | 0.6% | 0.7% | 0.9% | 0.7% | 13.1% |
| Schizophrenia | 2013 | 293 | 43644.5 | 0.7% | 0.6% | 0.8% | 0.9% | 0.7% | 13.1% |
| Schizophrenia | 2014 | 258 | 38363.5 | 0.7% | 0.6% | 0.8% | 0.9% | 0.7% | 5.1% |
| Skin cancer | 2000 | 53 | 3234 | 1.6% | 1.3% | 2.1% | 0.8% | 0.6% | 14.3% |
| Skin cancer | 2001 | 161.5 | 9424.5 | 1.7% | 1.5% | 2.0% | 2.5% | 0.5% | 15.7% |
| Skin cancer | 2002 | 276.5 | 15243 | 1.8% | 1.6% | 2.0% | 4.1% | 0.5% | 22.8% |
| Skin cancer | 2003 | 375.5 | 20512 | 1.8% | 1.7% | 2.0% | 1.2% | 0.9% | 4.5% |
| Skin cancer | 2004 | 474.5 | 25003 | 1.9% | 1.7% | 2.1% | 1.5% | 1.0% | 13.5% |
| Skin cancer | 2005 | 584 | 29065 | 2.0% | 1.9% | 2.2% | 1.7% | 1.4% | 2.4% |
| Skin cancer | 2006 | 695 | 32942.5 | 2.1% | 2.0% | 2.3% | 1.7% | 1.4% | 2.4% |
| Skin cancer | 2007 | 814 | 36548.5 | 2.2% | 2.1% | 2.4% | 1.9% | 1.6% | 2.5% |
| Skin cancer | 2008 | 932.5 | 39402.5 | 2.4% | 2.2% | 2.5% | 1.8% | 1.6% | 2.3% |
| Skin cancer | 2009 | 1058.5 | 42274.5 | 2.5% | 2.4% | 2.7% | 1.9% | 1.6% | 2.4% |
| Skin cancer | 2010 | 1178 | 43957.5 | 2.7% | 2.5% | 2.8% | 1.6% | 1.4% | 7.7% |
| Skin cancer | 2011 | 1274 | 45143.5 | 2.8% | 2.7% | 3.0% | 1.5% | 1.2% | 25.0% |
| Skin cancer | 2012 | 1341.5 | 45639.5 | 3.0% | 2.8% | 3.1% | 1.7% | 1.5% | 13.8% |
| Skin cancer | 2013 | 1321 | 43644.5 | 3.0% | 2.9% | 3.2% | 2.0% | 1.5% | 13.9% |
| Skin cancer | 2014 | 1196 | 38363.5 | 3.1% | 2.9% | 3.3% | 1.8% | 1.5% | 6.0% |
| Stomach cancer | 2000 | 3 | 3234 | 0.1% | 0.0% | 0.3% | 0.0% | 0.0% | 13.8% |
| Stomach cancer | 2001 | 7 | 9424.5 | 0.1% | 0.0% | 0.2% | 0.0% | 0.0% | 12.1% |
| Stomach cancer | 2002 | 10.5 | 15243 | 0.1% | 0.0% | 0.1% | 0.0% | 0.0% | 16.1% |
| Stomach cancer | 2003 | 15.5 | 20512 | 0.1% | 0.0% | 0.1% | 0.0% | 0.0% | 3.5% |
| Stomach cancer | 2004 | 16.5 | 25003 | 0.1% | 0.0% | 0.1% | 0.0% | 0.0% | 12.4% |
| Stomach cancer | 2005 | 17 | 29065 | 0.1% | 0.0% | 0.1% | 0.0% | 0.0% | 0.6% |
| Stomach cancer | 2006 | 24.5 | 32942.5 | 0.1% | 0.1% | 0.1% | 0.0% | 0.0% | 0.5% |
| Stomach cancer | 2007 | 31.5 | 36548.5 | 0.1% | 0.1% | 0.1% | 0.1% | 0.0% | 0.5% |
| Stomach cancer | 2008 | 37 | 39402.5 | 0.1% | 0.1% | 0.1% | 0.1% | 0.0% | 0.5% |
| Stomach cancer | 2009 | 40.5 | 42274.5 | 0.1% | 0.1% | 0.1% | 0.1% | 0.0% | 0.5% |
| Stomach cancer | 2010 | 41 | 43957.5 | 0.1% | 0.1% | 0.1% | 0.0% | 0.0% | 6.3% |
| Stomach cancer | 2011 | 41 | 45143.5 | 0.1% | 0.1% | 0.1% | 0.0% | 0.0% | 24.2% |
| Stomach cancer | 2012 | 41.5 | 45639.5 | 0.1% | 0.1% | 0.1% | 0.0% | 0.0% | 12.3% |
| Stomach cancer | 2013 | 38.5 | 43644.5 | 0.1% | 0.1% | 0.1% | 0.0% | 0.0% | 12.3% |
| Stomach cancer | 2014 | 36 | 38363.5 | 0.1% | 0.1% | 0.1% | 0.0% | 0.0% | 4.4% |
| Substance abuse | 2000 | 40 | 3234 | 1.2% | 0.9% | 1.7% | 2.4% | 1.1% | 16.2% |
| Substance abuse | 2001 | 135.5 | 9424.5 | 1.4% | 1.2% | 1.7% | 3.0% | 1.5% | 14.5% |
| Substance abuse | 2002 | 234.5 | 15243 | 1.5% | 1.4% | 1.7% | 3.8% | 2.2% | 19.1% |
| Substance abuse | 2003 | 334 | 20512 | 1.6% | 1.5% | 1.8% | 3.1% | 2.2% | 6.4% |
| Substance abuse | 2004 | 422 | 25003 | 1.7% | 1.5% | 1.9% | 3.3% | 2.5% | 15.5% |
| Substance abuse | 2005 | 512.5 | 29065 | 1.7% | 1.6% | 1.9% | 3.5% | 2.9% | 4.5% |
| Substance abuse | 2006 | 634.5 | 32942.5 | 1.9% | 1.8% | 2.1% | 4.0% | 3.2% | 5.0% |
| Substance abuse | 2007 | 756.5 | 36548.5 | 2.0% | 1.9% | 2.2% | 3.8% | 3.2% | 4.7% |
| Substance abuse | 2008 | 886.5 | 39402.5 | 2.2% | 2.1% | 2.4% | 4.4% | 3.7% | 5.4% |
| Substance abuse | 2009 | 1010.5 | 42274.5 | 2.3% | 2.2% | 2.5% | 4.7% | 4.0% | 5.7% |
| Substance abuse | 2010 | 1138.5 | 43957.5 | 2.5% | 2.4% | 2.7% | 6.2% | 5.0% | 12.1% |
| Substance abuse | 2011 | 1255 | 45143.5 | 2.7% | 2.6% | 2.9% | 6.6% | 5.1% | 29.0% |
| Substance abuse | 2012 | 1397 | 45639.5 | 2.9% | 2.9% | 3.2% | 6.6% | 5.3% | 17.9% |
| Substance abuse | 2013 | 1452.5 | 43644.5 | 3.2% | 3.2% | 3.5% | 6.4% | 5.4% | 18.5% |
| Substance abuse | 2014 | 1355.5 | 38363.5 | 3.4% | 3.4% | 3.7% | 7.5% | 5.9% | 11.8% |
| Unspecified cancer | 2000 | 24.5 | 3234 | 0.8% | 0.5% | 1.1% | 0.6% | 0.2% | 14.2% |
| Unspecified cancer | 2001 | 83 | 9424.5 | 0.9% | 0.7% | 1.1% | 0.4% | 0.3% | 12.3% |
| Unspecified cancer | 2002 | 135 | 15243 | 0.9% | 0.7% | 1.0% | 1.5% | 0.2% | 18.4% |
| Unspecified cancer | 2003 | 197 | 20512 | 1.0% | 0.8% | 1.1% | 0.7% | 0.4% | 4.1% |
| Unspecified cancer | 2004 | 232.5 | 25003 | 0.9% | 0.8% | 1.1% | 0.6% | 0.4% | 12.9% |
| Unspecified cancer | 2005 | 278.5 | 29065 | 1.0% | 0.9% | 1.1% | 0.7% | 0.5% | 1.3% |
| Unspecified cancer | 2006 | 322.5 | 32942.5 | 1.0% | 0.9% | 1.1% | 0.7% | 0.6% | 1.3% |
| Unspecified cancer | 2007 | 382 | 36548.5 | 1.0% | 0.9% | 1.2% | 0.8% | 0.6% | 1.2% |
| Unspecified cancer | 2008 | 434 | 39402.5 | 1.1% | 1.0% | 1.2% | 0.8% | 0.6% | 1.2% |
| Unspecified cancer | 2009 | 469.5 | 42274.5 | 1.1% | 1.0% | 1.2% | 0.8% | 0.6% | 1.2% |
| Unspecified cancer | 2010 | 483.5 | 43957.5 | 1.1% | 1.0% | 1.2% | 0.7% | 0.4% | 6.8% |
| Unspecified cancer | 2011 | 513.5 | 45143.5 | 1.1% | 1.0% | 1.2% | 0.6% | 0.4% | 24.5% |
| Unspecified cancer | 2012 | 531 | 45639.5 | 1.2% | 1.1% | 1.3% | 0.8% | 0.5% | 12.9% |
| Unspecified cancer | 2013 | 521.5 | 43644.5 | 1.2% | 1.1% | 1.3% | 0.7% | 0.6% | 12.9% |
| Unspecified cancer | 2014 | 444.5 | 38363.5 | 1.2% | 1.1% | 1.3% | 0.6% | 0.5% | 4.8% |
