# Supplementary material for: Patterns and temporal trends of comorbidity among adult patients with incident cardiovascular disease in the UK between 2000 and 2014: A population-based cohort study
Source: PLoS Med. 2018 Mar 6;15(3):e1002513. doi: 10.1371/journal.pmed.1002513 (PMC5839540; doi:10.1371/journal.pmed.1002513)
Supplement: S1 Text — (DOCX) [file pmed.1002513.s011.docx]

**Healthcare Innovation**

**and Evaluation**

Study Design and Analysis Plan

Project: HCIE-PR-012

Document code: HCIE-PR-012-SDE-VX

1. Study title

Multimorbidity in patients with incident cardiovascular disease

2. Research question

In patients with incident non-fatal cardiovascular disease (ischaemic heart disease and stroke/TIA) in the UK:

- What is the prevalence of multimorbidity?
- What is the prevalence of the most common comorbidities?
- How do they differ by year, age, sex and socioeconomic status?

3. Rationale

Multimorbidity, which is defined as the presence of two or more conditions in the same individual, is increasingly becoming a burden for patients, practitioners and the healthcare system (1-3). The number of people living with multimorbidity will continue to rise as the population ages and fewer people suffer from premature death (4, 5). In the UK, it is estimated that 23% of adults are multimorbid, when defined as two or more conditions (1).

Cardiovascular disease (CVD) is a leading cause of morbidity and mortality globally, and is associated with a high burden of multimorbidities that play a role in disease prognosis and management (6-9). Since 1990, the global burden of ischaemic heart disease has increased despite a decrease in mortality (10), and the number of disability life-years attributable to ischaemic stroke increased has also increased globally (11).

Comorbidity is a concept related to multimorbidity, and is defined as conditions(s) diagnosed in addition to an index condition (9). Several studies have examined comorbid conditions in patients with prevalent cardiovascular disease (8, 12-18). In one study, among US Medicare beneficiaries who had a diagnosis of stroke or ischaemic heart disease, the prevalence of multimorbidity (defined as 3 or more conditions) was 70% and 61% respectively (8). Another reports that in a Medicare population of people with at least one of four index cardiovascular conditions (ischaemic heart disease, heart failure, atrial fibrillation, stroke), the most common comorbid conditions included arthritis, diabetes mellitus, chronic kidney disease, cognitive impairment, and depression (18). The American College of Cardiology/American Heart Association (ACC/AHA) report on Medicare beneficiaries with cardiovascular conditions including ischaemic heart disease, heart failure and atrial fibrillation and shows that the most common comorbidities are hypertension (81-86%) and hyperlipidaemia (64-69%) across the three conditions (18). One of the largest studies on 1.4 million participants examined the prevalence of multimorbidity in 36,000 patients with stroke in a primary care adult population demographically representative of Scotland, finding 94% of patients with stroke had additional morbidities from a list of 39 conditions (15).

The current literature on multimorbidity in cardiovascular disease has focused on prevalent cardiovascular disease. To our knowledge there are no studies that use an *incident* cardiovascular disease population to investigate the trends and patterns in prevalence of comorbidities and multimorbidity. Examining a population with incident cardiovascular disease allows comparison of like with like, by addressing issues related to survival time and time after diagnosis.

The objective of this paper is to examine the burden of comorbidity and multimorbidity in patients with non-fatal incident cardiovascular disease. We will investigate how prevalence of multimorbidity and 58 comorbid chronic conditions in these patients changes over time, and how patterns vary by age group, sex and socioeconomic status.

How this study adds value:

- Large representative study on a UK population
- Examines contemporary trends over time among patients with incident CVD
- Investigates a large number of chronic conditions
- Investigates differences by age, sex and SES

4. Objectives

- To describe prevalence of multimorbidity and comorbidity of chronic conditions in people with incident non-fatal CVD (ischaemic heart disease, stroke/TIA).
- To examine crude and age-standardised prevalence and frequencies, stratified by calendar year, age, sex and socioeconomic status

5. Study type / design

- Longitudinal, and
- Multiple cross-sectional by year

6. Dataset

- The UK Clinical Practice Research Datalink (CPRD) and linked Hospital Episode Statistics (HES) 01/01/2000 to 31/12/2014

7. Population selection

- Age >16 years old
- Linkage to CPRD and HES (i.e. 2000 -2014)
- No diagnosis of CVD prior to study start date (back to the 1 January 1985 in primary care and back to 1 January 2000 in the secondary care records), or within the first 12 months of registration with their general practice
- Registered with GP for one year or more
- Diagnosed with incident non-fatal cardiovascular disease (ischaemic heart disease and/or stroke/TIA)

10. Co-variates / other features

- Age
- Sex
- Socioeconomic status

11. Outcomes / target variables

We selected 56 chronic conditions that are considered clinically significant and highly prevalent in the UK. The conditions are based on three sources: 1) Quality and Outcomes Framework (UK), an incentive scheme for general practitioners in the UK, 2) Charlson comorbidity index, the most commonly used comorbidity index originally designed to predict inpatient hospital mortality; 3) US multiple chronic conditions list, chosen by the US department of health:

| adjustment disorder |
| --- |
| affective disorder |
| anxiety |
| arthritis (including osteoarthritis) |
| asthma |
| bipolar disorder |
| bladder cancer |
| breast cancer |
| cardiac arrhythmia |
| cervical cancer |
| chronic kidney disease |
| chronic obstructive pulmonary |
| colon cancer |
| connective tissue disease |
| dementia |
| depression |
| diabetes mellitus |
| eating disorder |
| ENT cancer |
| epilepsy |
| gout |
| heart failure |
| hemiplegia |
| HIV/AIDS |
| hyperlipidaemia |
| hypertension |
| learning disability |
| leukaemia |
| liver cancer |
| liver disease |
| lung cancer |
| lymphoma |
| metastatic cancer |
| obesity |
| oesophageal cancer |
| osteoporosis |
| other cancer |
| other female reproductive cancer (excl breast, cervical, ovarian) |
| other gastrointestinal cancer (excl oesophageal, stomach, liver, pancrease, colon, rectal) |
| other male reproductive cancer (excl prostate) |
| other respiratory cancer (excl lung) |
| other urological cancer (excl bladder, renal) |
| ovarian cancer |
| pancreatic cancer |
| peptic ulcer disease |
| peripheral arterial disease |
| prostate cancer |
| psychoses |
| rectal cancer |
| renal cancer |
| rheumatoid arthritis |
| schizophrenia |
| skin cancer |
| stomach cancer |
| substance abuse |
| unspecified cancer |

Diagnosis of comorbid conditions is taken as any chronic condition that has been diagnosed before the diagnosis of CVD, recorded between the study period of 01/01/2000 to 31/12/2014 (refer to methods for details).

13. Methods

## Patient population selection

Patients are included in the study if they have a first diagnosis of incident non-fatal cardiovascular disease recorded between 1^st^ January 2000 to 31^st^ December 2014.

## Calculation of inclusion time in study

Patients are to be included in the study from the time of diagnosis of CVD, so long as this is after the start of study on 1^st^ January 2000, the patient has at least 12 months registration at the practice and the diagnosis is made after the first 12 months if their current registration period, their practice deemed to be contributing ‘up-to-standard’ data, and they are more than 16 years old.

Patients ended their time in the study at the earliest date of: 1) end of study on 31^st^ December 2014, 2) death date, 3) date of transfer out of their practice, 4) date of last collection of the practice.

## Identification of diagnoses

A list of diagnostic codes from hospital (International Classification of Diseases, 10^th^ revision; ICD-10) and primary care (READ) coding schemes was used to identify diagnoses. The validated codes were compiled from 1) codelists previously used by The George Institute for Global Health, 2) CALIBER and other online code repositories, and 3) medical dictionary keyword searches, and the top 20 most prevalent codes in each codelist were manually checked for validity as clinical diagnoses.

## Diagnosis of cardiovascular disease

Diagnosis of cardiovascular disease is taken as the patient’s first recording of a diagnostic code for incident non-fatal ischaemic heart disease or stroke/TIA, so long as conditions are met as described above. Patients were excluded if their date of diagnosis was the same day as their death date.

## Diagnosis of condition (that contributes to comorbidity/multimorbidity)

Diagnosis of a prevalent comorbid or multimorbid conditions will be defined as the patient’s first recording of a diagnostic code for the condition.

A condition diagnosis was included if it was diagnosed before the cardiovascular disease diagnosis. A comorbidity is defined as a condition diagnosed in addition to the index condition of cardiovascular disease. Multimorbidity is defined as those with one or more conditions (in addition to cardiovascular disease).

## Calculation of condition counts in multimorbidity

The prevalence of multimorbidity over time will be calculated as a count of conditions. (N.B. This count does not include the index condition of cardiovascular disease).

## Stratification by age, sex and socioeconomic status

Prevalence and incidence will be stratified by age groups (10 year age-bands according to groups defined in European Standard Population 2013: <20 ,20-29, 30-39, 40-49, 50-59, 60-69, 70-79, 80-89, >90), sex and socioeconomic status (index of multiple deprivation quintiles).

# Statistical analyses

Baseline characteristics are represented as frequencies and percentages for categorical data, medians and interquartile range for non-normal continuous data and means and standard deviations for normal continuous data, and stratified by sex and socioeconomic status quintile for all CVD patients at the time of diagnosis.

Annual incidence rates for CVD and component conditions were calculated by dividing the number incident cases by the number of patient-years at risk in the cohort for that particular year. Time at risk for each patient was specified to start at the latest of: the start of the year, the number of days alive, aged over 16, currently registered to a general practice for over 12 months and after the practice’s up-to- standard date; and specified to finish at the earliest of: the end of the year, date of death, transfer out of practice date of the practice’s last date of collection.

Prevalence rates for each comorbid condition or category of counts of conditions were calculated by dividing the number of cases by all patients diagnosed with CVD meeting the study inclusion criteria. Annual prevalence rates included all cases and CVD patients with a diagnosis recorded on the 1^st^ of July of the year of interest. Age, sex and SES specific prevalence rates were calculated using groupings as specified above.

Age and sex-standardised rates were computed by applying direct age-standardisation to the 2013 European Standard Population using 10-year age bands up to 90+ years old, and averaging the sex-specific age-standardised rates.

13b. Mock tables and figures

# Main tables and figures

*baseline_table.R, inccvd_incidence.R*

Table: Patient characteristics at time of diagnosis of cardiovascular disease

*baseline.txt*

Figure: Annual crude and age-sex-standardised incidence of cardiovascular disease 2000-2014, overall and by individual diseases, stratified by sex

*cvd_incidence.png*

*mmcount.R*

Figure: Crude and age-sex-standardised prevalence of counts of conditions in all patients with incident CVD 2000-2014

*mmcount_overall.png*

Figure: Crude and age-sex-standardised prevalence of condition count by calendar year

*mmcount_year.png*

Figure: Crude and age-standardised prevalence of condition count in all patients with incident CVD 2000-2014, stratified by sex

*mmcount_sex.png*

Figure: Crude and sex-standardised prevalence of condition count in all patients with incident CVD 2000-2014, stratified by age

*mmcount_age.png*

Figure: Crude and age-sex-standardised prevalence of condition count in all patients with incident CVD 2000-2014, stratified by socioeconomic status

*mmcount_ses.png*

*comorbidity.R*

Figure: Crude and age-sex-standardised prevalence of top 20 comorbid conditions in all patients with incident CVD 2000-2014

*comorbidity_overall.png*

Figure: Crude and age-sex-standardised prevalence of overall top 20 comorbid conditions by calendar year

*comorbidity_year.png*

Figure: Crude and age-standardised prevalence of overall top 20 comorbid conditions in all patients with incident CVD 2000-2014, stratified by sex

*comorbidity_sex.png*

Figure: Crude and sex-standardised prevalence of overall top 20 comorbid conditions in all patients with incident CVD 2000-2014, stratified by age

*comorbidity_age.png*

Figure: Crude and age-sex-standardised prevalence of overall top 20 comorbid conditions in all patients with incident CVD 2000-2014, stratified by socioeconomic status

*comorbidity_ses.png*

# Appendix

Table A1: Crude and age-standardised prevalence of comorbid conditions in all patients with incident CVD 2000-2014

14. Dissemination plans with rough timelines

| **Date** | **Task** |
| --- | --- |
| 16/8/2016 | Finalise analysis plan |
| 17/8/2016 | Extraction of target population subset |
|  | Analysis |
| 22/8/2016 | Preliminary results |
| 26/8/2016 | 1^st^ draft |
| 1/9/2016 | 2^nd^ draft |
| 6/9/2017 | Draft to Kazem and Robyn |
|  | Re-draft for submission |
| 1/10/2017 | Submission to PLOS medicine |
| 6/10/2017 | Final submission to PLOS medicine deadline |

15. References

1. Barnett K, Mercer SW, Norbury M, Watt G, Wyke S, Guthrie B. Epidemiology of multimorbidity and implications for health care, research, and medical education: a cross-sectional study. Lancet (London, England). 2012;380(9836):37-43.

2. Valderas JM, Starfield B, Sibbald B, Salisbury C, Roland M. Defining Comorbidity: Implications for Understanding Health and Health Services. Annals of family medicine. 2009;7(4):357-63.

3. Wallace E, Salisbury C, Guthrie B, Lewis C, Fahey T, Smith SM. Managing patients with multimorbidity in primary care. BMJ. 2015;350.

4. Taylor AW, Price K, Gill TK, Adams R, Pilkington R, Carrangis N, et al. Multimorbidity - not just an older person's issue. Results from an Australian biomedical study. BMC public health. 2010;10:718.

5. The Emerging Risk Factors C. ASsociation of cardiometabolic multimorbidity with mortality. JAMA. 2015;314(1):52-60.

6. Vos T, Barber RM, Bell B, Bertozzi-Villa A, Biryukov S, Bolliger I, et al. Global, regional, and national incidence, prevalence, and years lived with disability for 301 acute and chronic diseases and injuries in 188 countries, 1990&#x2013;2013: a systematic analysis for the Global Burden of Disease Study 2013. The Lancet.386(9995):743-800.

7. Vos T, Flaxman AD, Naghavi M. Years lived with disability (YLDs) for 1160 sequelae of 289 diseases and injuries 1990-2010: a systematic analysis for the global burden of disease study. Lancet (London, England). 2012;380.

8. Bell SP, Saraf AA. Epidemiology of Multimorbidity in Older Adults with Cardiovascular Disease. Clinics in Geriatric Medicine.32(2):215-26.

9. Boyd CM, Ritchie CS, Tipton EF, Studenski SA, Wieland D. From Bedside to Bench: summary from the American Geriatrics Society/National Institute on Aging Research Conference on Comorbidity and Multiple Morbidity in Older Adults. Aging Clinical and Experimental Research. 2008;20(3):181-8.

10. Moran AE, Forouzanfar MH, Roth G, Mensah GA, Ezzati M, Flaxman A, et al. The Global Burden of Ischemic Heart Disease in 1990 and 2010: The Global Burden of Disease 2010 Study. Circulation. 2014.

11. Feigin VL, Krishnamurthi RV, Parmar P, Norrving B, Mensah GA, Bennett DA, et al. Update on the Global Burden of Ischemic and Hemorrhagic Stroke in 1990-2013: The GBD 2013 Study. Neuroepidemiology. 2015;45(3):161-76.

12. Boyd CM, Leff B, Wolff JL, Yu Q, Zhou J, Rand C, et al. Informing Clinical Practice Guideline Development and Implementation: Prevalence of Coexisting Conditions Among Adults with Coronary Heart Disease. Journal of the American Geriatrics Society. 2011;59(5):797-805.

13. Chen HY, Saczynski JS, McManus DD, Lessard D, Yarzebski J, Lapane KL, et al. The impact of cardiac and noncardiac comorbidities on the short-term outcomes of patients hospitalized with acute myocardial infarction: a population-based perspective. Clin Epidemiol. 2013;5:439-48.

14. McManus DD, Nguyen HL, Saczynski JS, Tisminetzky M, Bourell P, Goldberg RJ. Multiple cardiovascular comorbidities and acute myocardial infarction: temporal trends (1990-2007) and impact on death rates at 30 days and 1 year. Clin Epidemiol. 2012;4:115-23.

15. Gallacher KI, Batty GD, McLean G, Mercer SW, Guthrie B, May CR, et al. Stroke, multimorbidity and polypharmacy in a nationally representative sample of 1,424,378 patients in Scotland: implications for treatment burden. BMC Medicine. 2014;12(1):1-9.

16. Gallacher KI, Batty GD, McLean G, Mercer SW, Guthrie B, May CR, et al. Stroke, multimorbidity and polypharmacy in a nationally representative sample of 1,424,378 patients in Scotland: implications for treatment burden. BMC Medicine. 2014;12(1):151.

17. Alfredsson J, Alexander KP. Multiple Chronic Conditions in Older Adults with Acute Coronary Syndromes. Clinics in Geriatric Medicine. 2016;32(2):291-303.

18. Arnett DK, Goodman RA, Halperin JL, Anderson JL, Parekh AK, Zoghbi WA. AHA/ACC/HHS Strategies to Enhance Application of Clinical Practice Guidelines in Patients With Cardiovascular Disease and Comorbid ConditionsFrom the American Heart Association, American College of Cardiology, and U.S. Department of Health and Human Services. Journal of the American College of Cardiology. 2014;64(17):1851-6.

History review

| Version | Author | Date | Authorizer | Date |
| --- | --- | --- | --- | --- |
| 1 | J Tran | 15/08/2017 |  |  |
| 2 | J Tran | 17/08/2017 |  |  |
| 3 | J Tran | 24/08/2017 |  |  |
| 4 | J Tran | 25/08/2017 |  |  |
| 5 | K Rahimi | 26/08/2017 |  |  |
| 6 | J Tran | 29/08/2017 |  |  |
| 7 | J Tran | 30/08/2017 |  |  |
| 8 | J Tran | 31/08/2017 |  |  |
| 9 | J Tran | 02/09/2017 |  |  |
| 10 | K Rahimi | 04/09/2017 |  |  |
| 11 | J Tran | 14/09/2017 |  |  |
| 12 | J Tran | 19/09/2017 |  |  |
| 13 | J Tran | 19/09/2017 |  |  |
